# Gut microbiota-modulated glutamic acid rejuvenates the quality of oocytes deteriorated by advanced reproductive age

**DOI:** 10.1038/s44321-026-00443-3

**Published:** 2026-05-08

**Authors:** Feixue Wang, Wenjun Zeng, Zihao Zhang, Na Li, Zhaokang Cui, Jie Bai, Jiner Yan, Yu Zhang, Yilong Miao, Ling Gu, Bo Xiong

**Affiliations:** 1 College of Animal Science and Technology, Nanjing Agricultural University, Nanjing, 210095 China; 2 College of Animal Sciences, Zhejiang University, Hangzhou, 310058 China

**Keywords:** Microbiology, Virology & Host Pathogen Interaction, Urogenital System

## Abstract

The gut microbiota plays a vital role in maintaining the physiological function of host health and the pathogenesis of various diseases. However, its relationship with maternal age-associated decline in oocyte quality remains elusive. Here, we report that establishment of gut microbiota from young donors in aged mice by fecal microbiota transplantation (FMT) is an effective method to rejuvenate the quality of maternally aged oocytes. Specifically, young gut microbiota promoted the ovulation and maturation of aged oocytes, and inhibited occurrence of cytoplasm fragmentation and spindle/chromosome abnormalities, hence enhancing the oocyte quality and female fertility. By integrating metagenome and untargeted metabolome of intestinal digesta, as well as targeted metabolome of ovaries and micro-transcriptome of oocytes, we identified that *Bacteroides_caecimuris*-modulated glutamic acid levels mediated the restorative effects of young gut microbiota on the aged oocytes through strengthening the mitochondria function. In addition, we demonstrated that in vivo supplementation of glutamic acid also enhanced the quality of aged oocytes, and the improvement of oocyte quality by glutamic acid was conserved across species. Altogether, our findings highlight the importance of gut microbiota in the oocyte aging and provide potential improvement strategies for age-related decline in oocyte quality and female fertility.

## Synopsis

A gut microbiota-metabolite axis was identified as a regulator of maternal oocyte aging, revealing that young donor microbiota and glutamic acid supplementation can restore mitochondrial function and improve fertility in aged females.

- Oocyte quality and female fertility in aged mice were significantly improved by fecal microbiota transplantation (FMT) from young donors.
- A Bacteroides caecimuris-glutamic acid axis was identified to mediate microbiota-driven rejuvenation of aged oocytes through restoration of mitochondrial function.
- Cross-species conservation of glutamic acid-mediated oocyte quality improvement was demonstrated, supporting its translational potential for age-related fertility decline.

## Abstract (web summary)

A gut microbiota-metabolite axis was identified as a regulator of maternal oocyte aging, revealing that young donor microbiota and glutamic acid supplementation can restore mitochondrial function and improve fertility in aged females.

## Graphical abstract

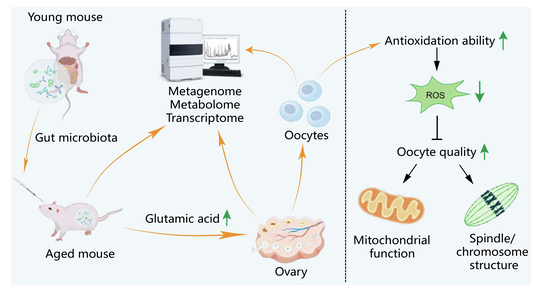

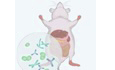

The paper explained **Problem**

Advanced maternal age is a major risk factor for female infertility and poor reproductive outcomes, largely due to the progressive decline in oocyte quality. Although age-related deterioration of oocytes has been extensively studied at the cellular and molecular levels, the contribution of systemic factors, particularly the gut microbiota, remains poorly understood. Given the emerging role of the gut microbiome in regulating host metabolism and aging, it is important to determine whether age-associated changes in gut microbiota contribute to oocyte aging and whether microbiota-targeted interventions may improve female fertility.

**Results**

We demonstrate that transplantation of gut microbiota from young donor mice into aged female mice effectively rejuvenates the quality of aged oocytes and improves fertility. Young gut microbiota significantly enhanced ovulation and oocyte maturation, while reducing cytoplasmic fragmentation and spindle/chromosome abnormalities. Multi-omics analyses integrating metagenomics, intestinal and ovarian metabolomics, and oocyte transcriptomics identified *Bacteroides caecimuris*–regulated glutamic acid metabolism as a key mediator of this effect. Mechanistically, elevated glutamic acid levels improved mitochondrial function in aged oocytes, thereby restoring developmental competence. Importantly, in vivo glutamic acid supplementation alone was sufficient to improve aged oocyte quality, and this beneficial effect was conserved across species.

**Impact**

Our study provides new mechanistic insights into the gut microbiota-ovary axis in reproductive aging by identifying microbial metabolite-mediated mitochondrial protection as a key mechanism underlying oocyte rejuvenation. These findings provide proof-of-concept evidence that modulation of the gut microbiota or targeted metabolic supplementation may represent promising therapeutic strategies to improve age-related decline in oocyte quality and female fertility, with potential translational implications for reproductive medicine and assisted reproduction in women of advanced maternal age.

## Introduction

Infertility has become a global problem affecting one in six people worldwide, as indicated by the latest report released by the World Health Organization in 2023 (World Health Organization, 2023). One of the most significant contributors to human infertility is the age, particularly after 38 years old in women. Although maternal age influences almost all aspects of female reproduction, the main causes for age-related infertility include reduced ovarian reserve and decreased oocyte competence (Cimadomo et al, 2018). Assisted reproductive technology (ART) has been used to treat the infertility for decades, but its success rate still remains low due to the limited quantity and quality of oocytes, especially for women of advanced reproductive age (Malizia et al, 2009). Thus, acquisition of high-quality oocytes is the key to improve the reproductive outcomes for both natural pregnancy and ART.

Microbiota, composed of bacteria, fungi, viruses, and protists, is a vast ecosystem containing over 100 trillion microbes that inhabit in various parts of human body, including the skin, mouth, respiratory system, gastrointestinal system, and vagina (Iliev and Leonardi, 2017; Richard and Sokol, 2019; Sarin et al, 2019; Sender et al, 2016; Shkoporov and Hill, 2019). Notably, in the intestinal tract, gut microbiota has developed a symbiotic relationship with the human body through millions of years of coevolution, and thus plays vital roles in maintaining the physiological health of the human body by contributing to the metabolic protection, structural and histological functions, and other essential processes (Michaudel and Sokol, 2020; Prakash et al, 2011; Velagapudi et al, 2010). It has been demonstrated that gut microbiota participates in the regulation of host metabolic homeostasis via the synthesis of vitamin, amino acids, short-chain free fatty acids (SCFAs), and conjugated linoleic acid, the biotransformation of bile acids, as well as the synthesis and detoxification of ammonia (Altuntas and Batman, 2017; Bodogai et al, 2018; Human Microbiome Project, 2012; Thevaranjan et al, 2017; Vaiserman et al, 2017). However, the composition of gut microbiota is not static and could be influenced by many factors such as age, dietary habits, lifestyle, and genetic predisposition (Ottman et al, 2012). A growing body of evidence has indicated that the dysbiosis of gut microbiota is highly correlated with a variety of diseases, including diabetes, cardiovascular disease, cancer, Parkinson’s disease, inflammatory bowel disease, allergies, asthma, and lupus (Altuntas and Batman, 2017; Fallucca et al, 2014). In particular, age-related changes in gut microbiota have been widely observed in animals, from insects to mammals, hence the age-related dysbiosis may be a significant contributor to the increased incidence of many age-related diseases (Han et al, 2017; Langille et al, 2014).

Fecal microbiota transplantation (FMT) is a valuable tool for uncovering the function of gut microbiota in the pathological processes, an effective method for modifying the composition of gut microbiota to overcome dysbiosis, and a promising treatment option for several diseases in recent years (Qu et al, 2022). Especially, FMT has been applied for the study of the relationship between gut microbiota and reproduction. It has been reported that FMT from an excessive-energy diet-induced metabolic syndrome sheep impairs the spermatogenesis in mice (Zhang et al, 2022). On the contrary, FMT from alginate oligosaccharide-dosed mice improves the sperm quality and spermatogenesis in busulfan-treated mice (Zhang et al, 2021a). In addition, FMT from women with polycystic ovary syndrome (PCOS) leads to an increase in ovarian dysfunction in recipient mice (Qi et al, 2019). Whereas FMT from healthy rats recovers the estrus cycle, hormonal levels and ovarian morphology in rats with PCOS (Guo et al, 2016). These observations indicate that gut microbiota is a critical regulator in both male and female reproduction. However, whether FMT could be used for improving the quality of maternally aged oocytes has remained unclear.

In the present study, we demonstrated the favorable effects of gut microbiota from young donors on the quality of aged oocytes by FMT. Taking advantage of metagenomic, metabolomic and transcriptomic analyses, we further discovered that young gut microbiota elevated the glutamic acid level to recover the redox homeostasis and mitochondrial function in aged oocytes, which is negatively correlated with the abundance of *Bacteroides_caecimuris* in the gut. We further validated that supplementation of glutamic acid alleviated the defects in oocytes induced by maternal aging.

## Results

### Metagenome sequencing reveals the remodeling of gut microbiota in aged mice by FMT from young donors

**Figure 1:**
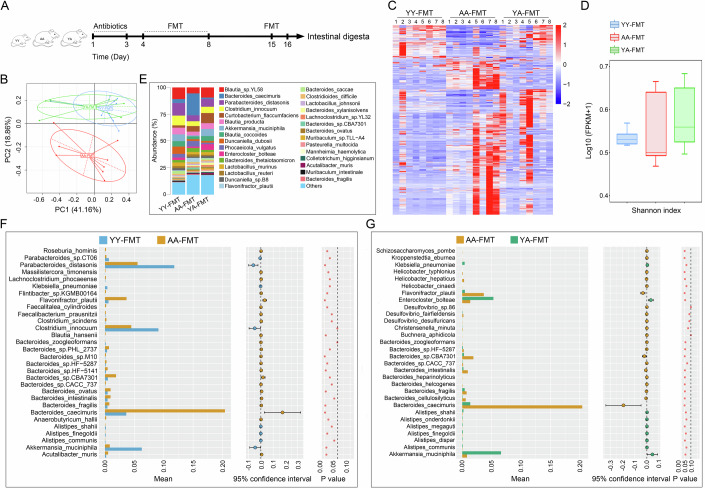
Gut microbiota composition after FMT in mice as analyzed by metagenome. (**A**) A timeline scheme for antibiotics treatment, FMT, and subsequent analyses. (**B**) PCA plot showing the samples from YY-FMT, AA-FMT, and YA-FMT mice. Blue circles represented samples from YY-FMT group, red circles represented samples from AA-FMT group, and green circles represented samples from YA-FMT group. (**C**) Heatmap illustration displayed the differential abundance of microbiota at species level in YY-FMT, AA-FMT, and YA-FMT mice. (**D**) Shannon index of gut microbiota in YY-FMT (*n* = 8), AA-FMT (*n* = 8), and YA-FMT (*n* = 8) mice was shown as box plots. The center line represents the median (50th percentile). The lower and upper bounds of the box represent the first quartile (25th percentile) and third quartile (75th percentile), respectively. The whiskers extend to the minimum and maximum values. (**E**) Bacterial taxonomic profiling at species level of intestinal bacteria in YY-FMT, AA-FMT, and YA-FMT mice. (**F**) Stamp plot showed the differential microbiota between YY-FMT and AA-FMT mice at species level. (**G**) Stamp plot showed the differential microbiota between YA-FMT and AA-FMT mice at species level. Statistical significance was determined by the two-sided Wilcoxon rank-sum test (**F**, **G**). Source data are available in Dataset EV1.

**Figure EV1:**
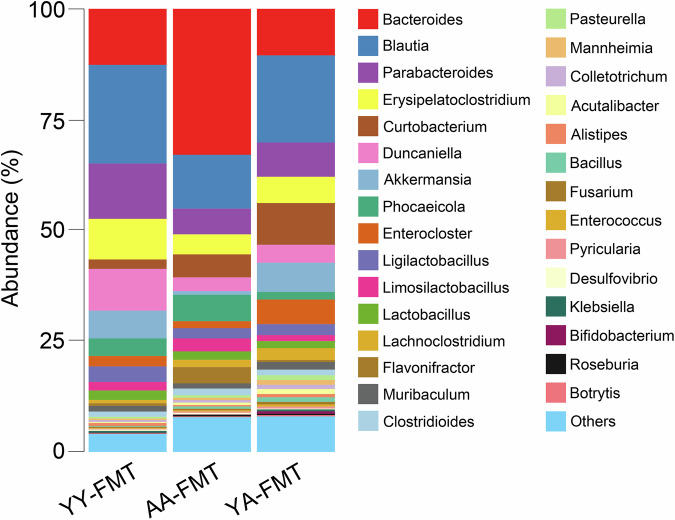
Effects of FMT on the bacterial abundance in mice. The degree of bacterial taxonomic similarity of gut microbiota was analyzed in YY-FMT, AA-FMT, and YA-FMT groups at genus level.

**Figure EV2:**
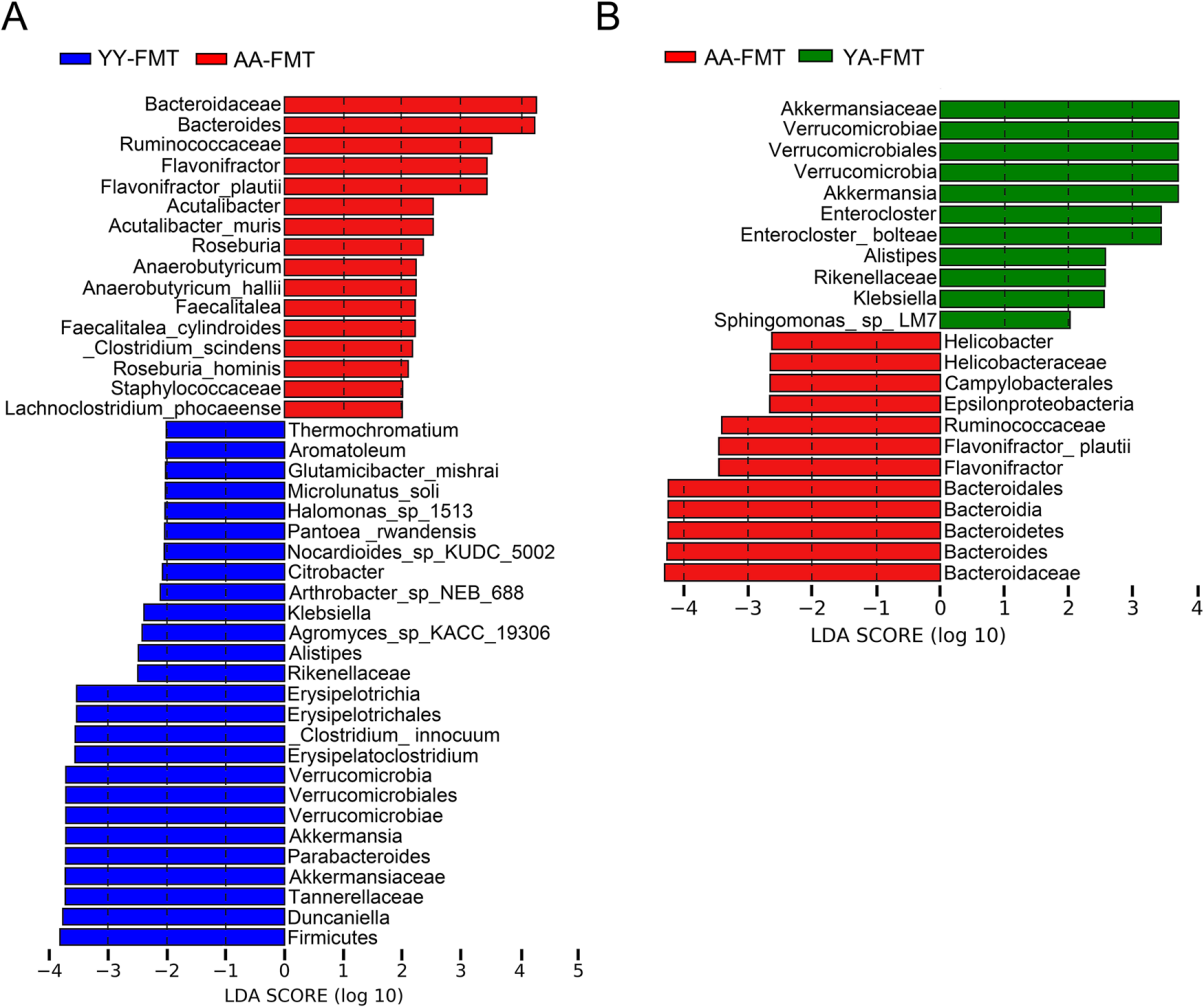
Effects of FMT on the bacterial taxa in mice. (**A**) LEfSe showed dominant bacterial taxa in AA-FMT group compared to YY-FMT group. Blue bar represented YY-FMT, and red bar represented AA-FMT. (**B**) LEfSe showed dominant bacterial taxa in YA-FMT group compared to AA-FMT group. Red bar represented AA-FMT, and green bar represented YA-FMT.

To establish the gut microbiota from young donors in aged mice, we applied antibiotics treatment to remove the original gut microbiota from recipient mice and then transplanted the gut microbiota from young donor mice into aged recipient mice (YA-FMT) (Fig. 1A). Metagenome sequencing of intestinal digesta was carried out to evaluate the changes of gut microbiota after FMT. The principal component analysis (PCA) showed that eight biological replicates clustered relatively together in each group, and the samples in YA-FMT group were close to those in young controls (YY-FMT, FMT from young donors to young recipients) but separated far from those in AA-FMT (FMT from aged donors to aged recipients) group (Fig. 1B). Moreover, the clustered heatmap at species level revealed that YA-FMT mice had a similar gut microbiota composition to that of young controls, which was remarkably different from the one in AA-FMT mice (Fig. 1C). Also, the α-diversity as assessed by Shannon index indicated that bacterial richness had a decreased trend in AA-FMT mice compared to YY-FMT mice, while elevated in YA-FMT mice (Fig. 1D). We further analyzed the degree of bacterial taxonomic similarity of gut microbiota in each group at genus level, and observed that in comparison with young controls, AA-FMT mice exhibited decreased abundance of *Blautia*, *Parabacteroides* and *Akkermansia*, and increased abundance of *Bacteroides*, *Phocaeicola* and *Flavonifractor*, which were restored in YA-FMT mice (Fig. EV1A). This observation was also confirmed by the linear discriminant analysis effect size (LEfSe), showing the dominant bacterial taxa in AA-FMT group compared to YY-FMT group and YA-FMT group compared to AA-FMT group, respectively (Fig. EV2A,B). Lastly, we examined the degree of bacterial taxonomic similarity as well as statistical analysis of taxonomic and functional profiles (STAMP) of gut microbiota at species level, and discovered that the abundance of *Bacteroides_caecimuris*, *Flavonifractor_plautii* and *Bacteroides_sp.CBA7301* was increased in AA-FMT mice compared to young controls and YA-FMT mice (Fig. 1E–G). In the meantime, the abundance of *Akkermansia_muciniphila* was reduced in AA-FMT mice compared to young controls and YA-FMT mice (Fig. 1E–G). Collectively, all above findings demonstrate that FMT from young donors effectively remodels the gut microbiota in recipient aged mice.

### Young gut microbiota enhances the oocyte quality and fertility of aged mice

**Figure 2:**
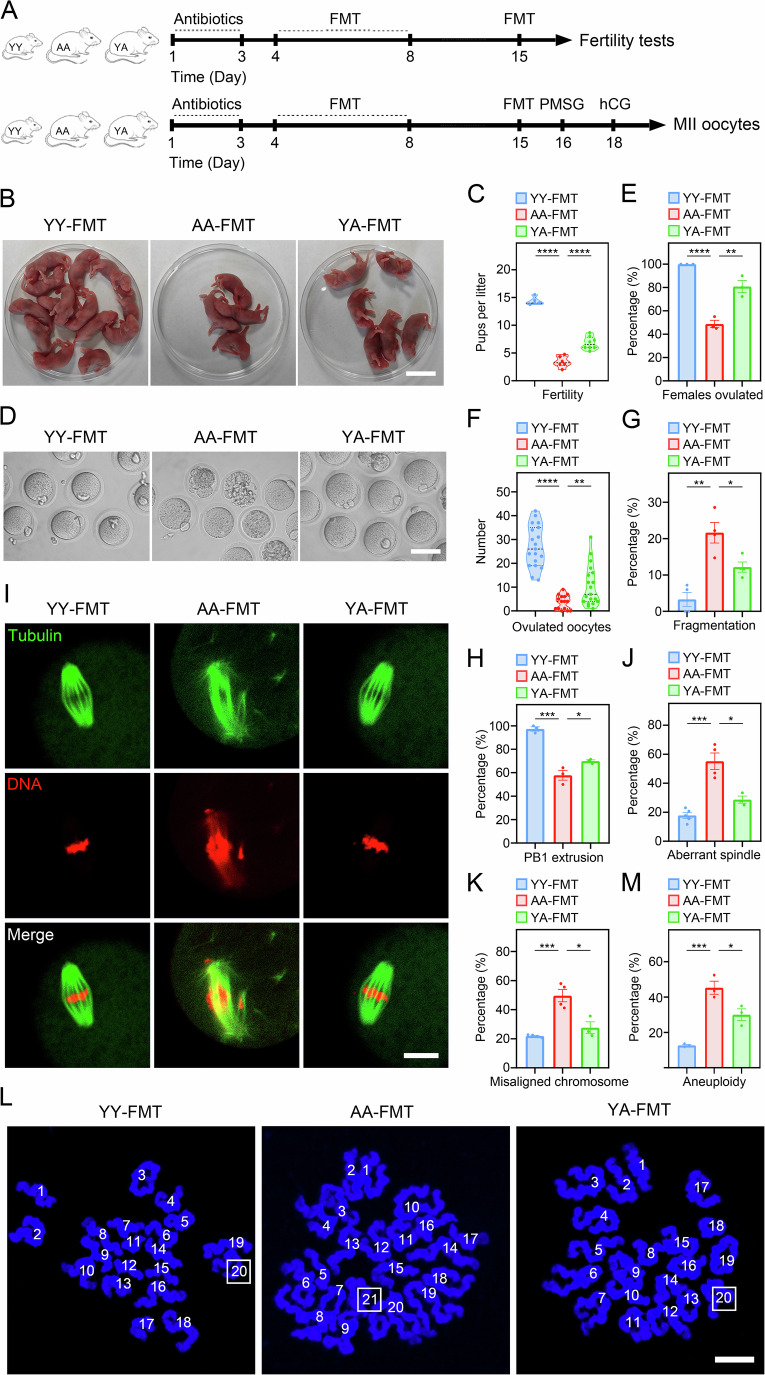
Effects of young gut microbiota on the quality of aged oocytes. (**A**) A timeline scheme for antibiotics treatment, FMT, hormone injection and subsequent analyses. (**B**) Representative images of pups delivered by YY-FMT, AA-FMT, and YA-FMT female mice. Scale bar, 2 cm. (**C**) The average pups per litter were counted in YY-FMT (*n* = 5), AA-FMT (*n* = 6), and YA-FMT (*n* = 8) groups. ****P < 0.0001, ****P < 0.0001. (**D**) Representative images of ovulated oocytes collected from YY-FMT, AA-FMT, and YA-FMT mice. Scale bar, 60 μm. (**E**) The percentage of female mice that were ovulated in YY-FMT (*n* = 35), AA-FMT (*n* = 53), and YA-FMT (*n* = 43) group. ****P < 0.0001, **P = 0.0061. (**F**) The number of ovulated oocytes was counted in YY-FMT (*n* = 19), AA-FMT (*n* = 15), and YA-FMT (*n* = 19) mice. ****P < 0.0001, **P = 0.0055. (**G**) The proportion of fragmented oocytes was quantified in YY-FMT (*n* = 462), AA-FMT (*n* = 97), and YA-FMT (*n* = 128) mice. **P = 0.0017, *P = 0.0243. (**H**) The rate of PB1 extrusion was quantified in YY-FMT (*n* = 171), AA-FMT (*n* = 52), and YA-FMT (*n* = 72) oocytes. ***P = 0.0010, *P = 0.0492. (**I**) Representative images of the spindle morphology and chromosome alignment in YY-FMT, AA-FMT, and YA-FMT oocytes at MII stage. Scale bar, 20 μm. (**J**) The rate of aberrant spindles was quantified in YY-FMT (*n* = 332), AA-FMT (*n* = 61), and YA-FMT (*n* = 67) oocytes at MII stage. ***P = 0.0003, *P = 0.0134. (**K**) The rate of misaligned chromosomes was quantified in YY-FMT (*n* = 306), AA-FMT (*n* = 61), and YA-FMT oocytes (*n* = 67) at MII stage. ***P = 0.0006, *P = 0.0145. (**L**) Representative images of chromosome spreads in MII oocytes from YY-FMT, AA-FMT, and YA-FMT mice. Scale bar, 5 μm. (**M**) The rate of aneuploidy was quantified in YY-FMT (*n*  =  71), AA-FMT (*n*  =  74), and YA-FMT (*n*  =  57) oocytes at MII stage. ***P = 0.0009, *P = 0.0366. Data in (**C**), (**E**), (**F**), (**G**), (**H**), (**J**), (**K**), and (**M**) were presented as mean ± SEM or SD of at least three independent experiments. *P < 0.05; **P < 0.01; ***P < 0.001; ****P < 0.0001. Statistical significance was determined by two-tailed unpaired t-test. Source data are available online for this figure.

**Figure EV3:**
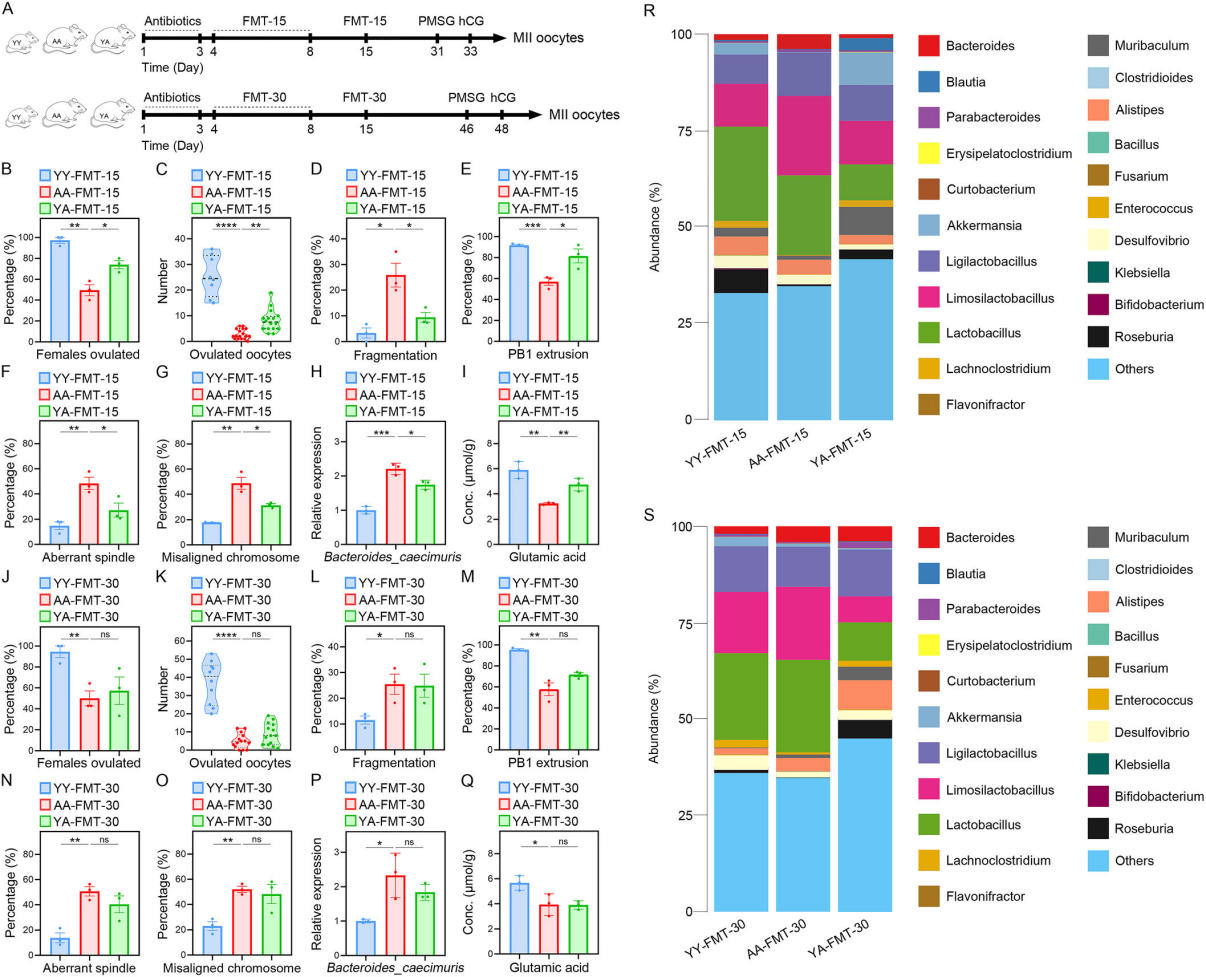
Effects of young gut microbiota on the quality of aged oocytes on days 15 and 30 after FMT. (**A**) A timeline scheme for antibiotics treatment, FMT, hormone injection and subsequent analyses. (**B**) The percentage of female mice that were ovulated in YY-FMT-15 (*n* = 30), AA-FMT-15 (*n* = 36), and YA-FMT-15 (*n* = 38) group. **P = 0.0013, *P = 0.0205. (**C**) The number of ovulated oocytes was counted in YY-FMT-15 (*n* = 8), AA-FMT-15 (*n* = 17), and YA-FMT-15 (*n* = 16) mice. ****P < 0.0001, **P = 0.0032. (**D**) The proportion of fragmented oocytes was quantified in YY-FMT-15 (*n* = 112), AA-FMT-15 (*n* = 67), and YA-FMT-15 (*n* = 96) mice. *P = 0.0109, *P = 0.0303. (**E**) The rate of PB1 extrusion was quantified in YY-FMT-15 (*n* = 112), AA-FMT-15 (*n* = 67), and YA-FMT-15 (*n* = 96) oocytes. **P = 0.0007, *P = 0.0312. (**F**) The rate of aberrant spindles was quantified in YY-FMT-15 (*n* = 73), AA-FMT-15 (*n* = 110), and YA-FMT-15 (*n* = 67) oocytes at MII stage. **P = 0.0044, *P = 0.0445. (**G**) The rate of misaligned chromosomes was quantified in YY-FMT-15 (*n* = 73), AA-FMT-15 (*n* = 110), and YA-FMT-15 oocytes (*n* = 67) at M II stage. **P = 0.0029, *P = 0.0241. (**H**) The abundance of *Bacteroides_caecimuris* was determined by qPCR in YY-FMT-15, AA-FMT-15, and YA-FMT-15 mice. ***P = 0.0005, *P = 0.0186. (**I**) Glutamic acid levels were quantified in YY-FMT-15 (*n* = 21), AA-FMT-15 (*n* = 16), and YA-FMT-15 (*n* = 15) ovaries. **P = 0.0073, **P = 0.0024. (**J**) The percentage of female mice that were ovulated in YY-FMT-30 (*n* = 32), AA-FMT-30 (*n* = 42), and YA-FMT-30 (*n* = 36) group. **P = 0.0080, *P* = 0.6508. (**K**) The number of ovulated oocytes was counted in YY-FMT-30 (*n* = 10), AA-FMT-30 (*n* = 14), and YA-FMT-30 (*n* = 14) mice. ****P < 0.0001, *P* = 0.0604. (**L**) The proportion of fragmented oocytes was quantified in YY-FMT-30 (*n* = 492), AA-FMT-30 (*n* = 217), and YA-FMT-30 (*n* = 151) mice. *P = 0.0299, *P* = 0.9346. (**M**) The rate of PB1 extrusion was quantified in YY-FMT-30 (*n* = 492), AA-FMT-30 (*n* = 217), and YA-FMT-30 (*n* = 151) oocytes. **P = 0.0035, *P* = 0.0931. (**N**) The rate of aberrant spindles was quantified in YY-FMT-30 (*n* = 98), AA-FMT-30 (*n* = 114), and YA-FMT-30 (*n* = 107) oocytes at M II stage. **P = 0.0024, *P* = 0.2500. (**O**) The rate of misaligned chromosomes was quantified in YY-FMT-30 (*n* = 98), AA-FMT-30 (*n* = 114), and YA-FMT-30 oocytes (*n* = 107) at MII stage. **P = 0.0023, *P* = 0.6616. (**P**) The abundance of *Bacteroides_caecimuris* was determined by qPCR in YY-FMT-30, AA-FMT-30, and YA-FMT-30 mice. *P = 0.0235, *P* = 0.2804. (**Q**) Glutamic acid levels were quantified in YY-FMT-30 (*n* = 22), AA-FMT-30 (*n* = 16), and YA-FMT-30 (*n* = 16) ovaries. **P = 0.0453, *P* = 0.9562. (**R**) The degree of bacterial taxonomic similarity of gut microbiota was analyzed in YY-FMT-15, AA-FMT-15, and YA-FMT-15 groups at genus level. (**S**) The degree of bacterial taxonomic similarity of gut microbiota was analyzed in YY-FMT-30, AA-FMT-30, and YA-FMT-30 groups at genus level. Data in (**B**–**Q**) were presented as mean ± SEM or SD of at least three independent experiments. *P < 0.05; **P < 0.01; ***P < 0.001; ****P < 0.0001; ns, no significance. Statistical significance was determined by two-tailed unpaired t-test.

To determine whether the remodeling of gut microbiota to a young-like composition would influence the female reproduction of aged animals, fertility tests were performed to count the number of pups (Fig. 2A). The data revealed that young gut microbiota significantly elevated the average number of pups per litter in aged group compared to the aged gut microbiota (Fig. 2B,C), indicative of the direct relationship between gut microbiota composition and female fertility. In addition, ovulated oocytes were retrieved from the oviduct after gonadotrophin priming in female mice (Fig. 2A), and it was found that all of young mice were able to ovulate a number of oocytes, whereas approximately half of aged mice did not ovulate any oocyte, which could be partially restored after transplantation of young gut microbiota (Fig. 2D,E). Quantitatively, the average number of ovulated oocytes was substantially reduced in aged mice compared to that in young ones, which was increased in YA-FMT group (Fig. 2F). Moreover, we observed a decreased rate of polar body extrusion (PBE) with a high occurrence of fragmentation in aged oocytes in comparison with the young ones (Fig. 2D,G,H). Young gut microbiota also rescued these developmental defects of oocytes caused by maternal aging (Fig. 2D,G,H). Given that the spindle/chromosome structure is a critical indicator for evaluating the oocyte quality, we stained the ovulated oocytes with α-tubulin antibody. The immunofluorescent images displayed a normal barrel-shaped spindle with well-aligned chromosomes was present in the young oocytes, however, a variety of disorganized spindles with misaligned chromosomes was observed in aged oocytes (Fig. 2I–K). Notably, young gut microbiota reduced the proportion of aberrant spindle and misaligned chromosomes in oocytes induced by aging (Fig. 2I–K), and thereby lowering the occurrence of aneuploidy in aged oocytes (Fig. 2L,M). To further ascertain how long the beneficial effects of young gut microbiota could last, we examined the quality of aged oocytes on days 15 and 30 after FMT. The data manifested that on day 15 post-FMT, young gut microbiota still increased the number of ovulated oocytes, promoted oocyte maturation, suppressed the oocyte fragmentation, and mitigated the spindle/chromosome abnormalities by maintaining the reduced abundance of *Bacteroides_caecimuris* and increased levels of glutamic acid in aged mice (Fig. EV3A–I). However, on day 30 post-FMT, young gut microbiota almost had no restorative impacts on the quality of aged oocytes (Fig. EV3J–Q). Consistently, analysis of the 16S rRNA sequencing data showed that the composition of *Bacteroides* in YA mice on day 30 after FMT increased to a level comparable to that observed in AA mice, whereas this similarity was not evident at day 15 (Fig. EV3R,S). These findings suggest that FMT effects last no more than 30 days. Altogether, our observations indicate that transplantation of gut microbiota from young donors is an effective method to improve the fertility and oocyte quality of aged animals.

### Young gut microbiota alters the metabolome profiling of aged mice

**Figure EV4:**
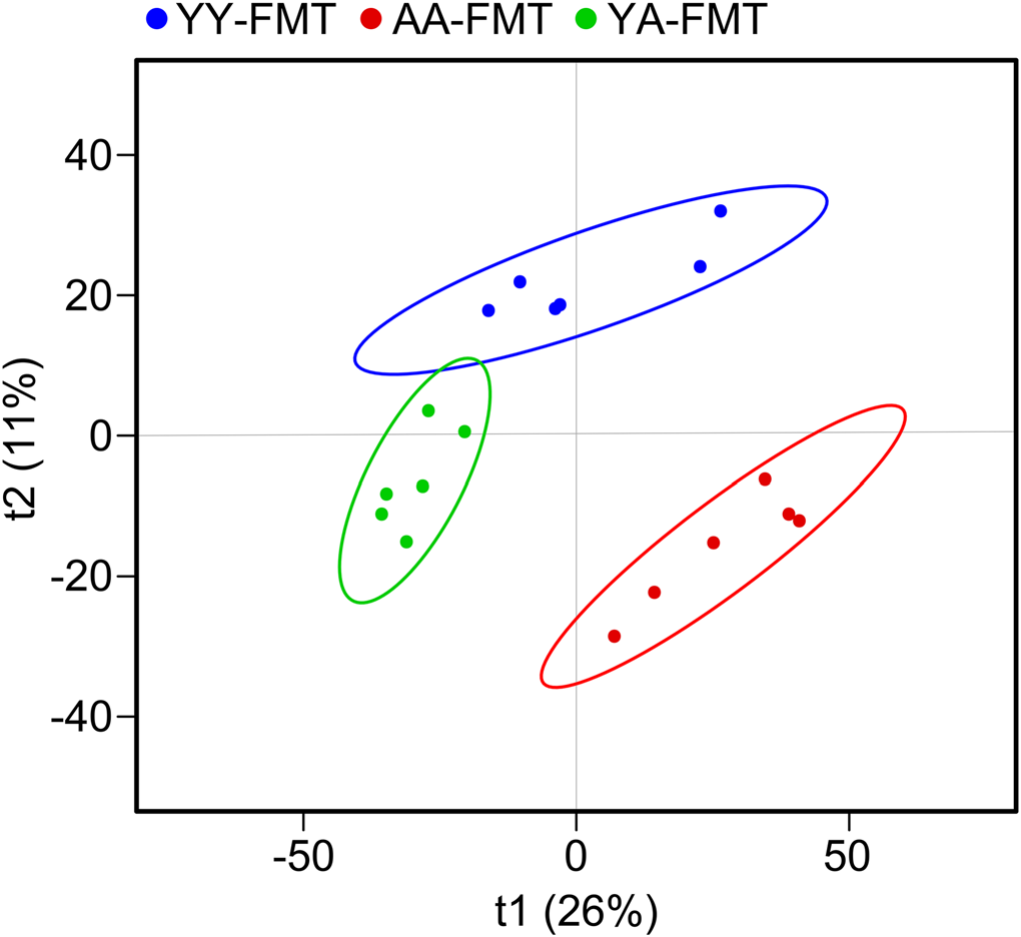
PLS-DA plot showing the metabolome samples from FMT mice. Blue circles represented samples from YY-FMT group, red circles represented samples from AA-FMT group, and green circles represented samples from YA-FMT group.

**Figure 3:**
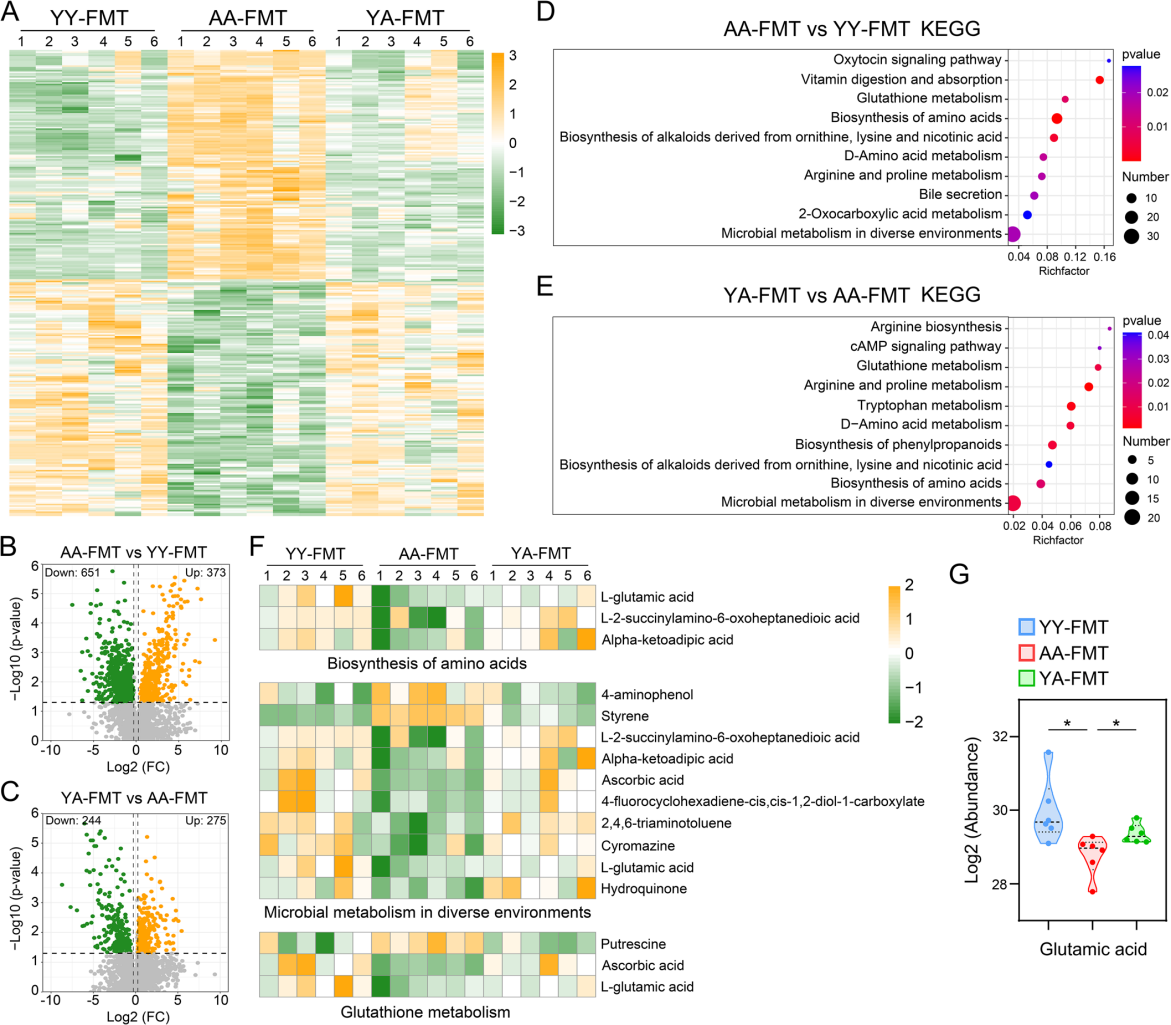
Effects of young gut microbiota on the metabolome profiling of intestinal digesta in aged mice. (**A**) Differential metabolites were clustered by heatmap in YY-FMT, AA-FMT, and YA-FMT mice. (**B**) Volcano plot showed the number of differential metabolites (downregulated, green; upregulated, yellow) in AA-FMT (*n* = 6) group compared to YY-FMT (*n* = 6) group. (**C**) Volcano plot showed the number of differential metabolites (downregulated, green; upregulated, yellow) in YA-FMT (*n* = 6) group compared to AA-FMT (*n* = 6) group. (**D**) Pathways in which differential metabolites were enriched as analyzed by KEGG in AA-FMT group compared to YY-FMT group. (**E**) Pathways in which differential metabolites were enriched as analyzed by KEGG in YA-FMT group compared to AA-FMT group. (**F**) Heatmap illustration displayed differential metabolites in the biosynthesis of amino acids, microbial metabolism in diverse environments, and glutathione metabolism pathways in YY-FMT, AA-FMT, and YA-FMT groups. (**G**) The levels of glutamic acid from metabolome data were showed in YY-FMT, AA-FMT, and YA-FMT groups. *P = 0.0177, *P = 0.0377. Data in (**G**) were presented as mean ± SD. *P < 0.05. Statistical significance was determined by the hypergeometric test (**D**, **E**) and two-tailed unpaired t-test (**B**, **C**, **G**). Source data are available online for this figure.

To explore how young gut microbiota rejuvenates the oocytes in aged mice, we next mapped the metabolite profiling of intestinal digesta by untargeted metabolomics. By partial least squares discriminant analysis (PLS-DA), we found that six sample replicates clustered together in each group, but separated from each other among groups (Fig. EV4). In particular, young gut microbiota changed the clustering of metabolites in aged group to a pattern more similar to the young control. Hierarchically-clustered heatmap of differential metabolites also displayed that the metabolome profile of intestinal digesta in aged mice was prominently distinct with the young controls, and partially recovered by young gut microbiota (Fig. 3A). Volcano plot further revealed the number of upregulated and downregulated metabolites in aged group compared to the young control as well as YA-FMT group compared to aged counterpart (Fig. 3B,C). By Kyoto Encyclopedia of Genes and Genomes (KEGG) enrichment analysis, we discovered that young gut microbiota restored the metabolite level in aged mice involved in various biological pathways (Fig. 3D,E). Among them, we noticed that glutamic acid participates in numerous pathways including microbial metabolism in diverse environments, biosynthesis of amino acids, and glutathione metabolism (Fig. 3F), and its level was significantly reduced in aged mice but elevated by young gut microbiota as shown in both heatmap and graph plotted from metabolome data (Fig. 3F,G). Therefore, our results suggest that glutamic acid might be a key metabolite that mediates the effects of gut microbiota from young donors on the aged mice.

### Integrated analysis of metagenome and metabolome uncovers the negative correlation between *Bacteroides_caecimuris* and glutamic acid

**Figure 4:**
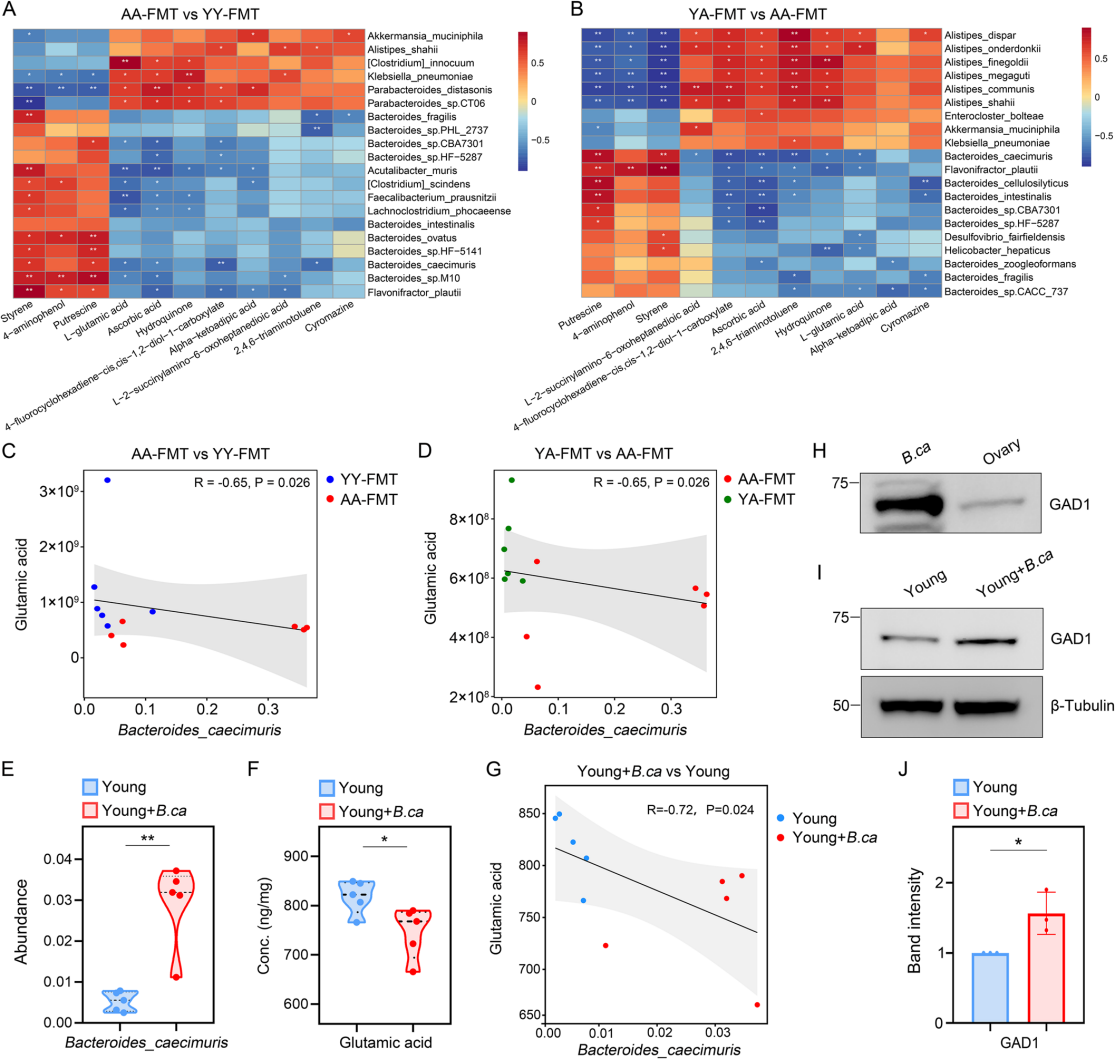
Integrated analysis of metagenome and metabolome data. (**A**) Heatmap illustration of Spearman’s rank correlation between metabolites from microbial metabolism in diverse environments, biosynthesis of amino acids, and glutathione metabolism pathways and top 20 abundant differential species-level bacteria in AA-FMT group compared to YY-FMT group. (**B**) Heatmap illustration of the Spearman’s rank correlation between the metabolites from microbial metabolism in diverse environments, biosynthesis of amino acids, and glutathione metabolism pathways and top 20 abundant differential species-level bacteria in YA-FMT group compared to AA-FMT group. (**C**) Spearman’s correlation analysis of glutamic acid with *Bacteroides_caecimuris* in AA-FMT group compared to YY-FMT group. (**D**) Spearman’s correlation analysis of glutamic acid with *Bacteroides_caecimuris* in YA-FMT group compared to AA-FMT group. (**E**) The abundance of *Bacteroides_caecimuris* from metagenome data was shown in young (*n* = 5) and young+*B.ca* (gavaged with *Bacteroides_caecimuris*, *n* = 5) mice. **P = 0.0010. (**F**) The levels of glutamic acid in the ovary from targeted metabolome data were shown in young (*n* = 5) and young+*B.ca* mice (*n* = 5). *P = 0.0324. (**G**) Spearman’s correlation analysis of glutamic acid level with *Bacteroides_caecimuris* abundance in young+*B.ca* mice compared to young mice. (**H**) Protein expression of GAD1 in *Bacteroides_caecimuris* and mouse ovary as assessed by immunoblotting. (**I**) Protein levels of GAD1 in ovaries from young and young+*B.ca* mice as assessed by immunoblotting. β-Tubulin was used as an internal control. (**J**) The band intensities of GAD1 were quantified in young (*n* = 3) and young+*B.ca* (*n* = 3) group, and normalized to β-Tubulin. *P = 0.0310. Data in (**E**), (**F**), and (**J**) were presented as mean ± SEM or SD. *P < 0.05; **P < 0.01. Statistical significance was determined by two-tailed unpaired t-test. Source data are available online for this figure.

We further performed the Spearman’s rank correlation analysis to investigate the functional relationship between gut microbiota and metabolites. The metabolites from microbial metabolism in diverse environments, biosynthesis of amino acids, and glutathione metabolism pathways were analyzed with the top 20 abundant differential species-level bacteria. Eighty-two statistically significant pairs were found between metabolites and bacteria in AA-FMT vs YY-FMT group, and ninety-seven statistically significant pairs were shown in YA-FMT vs AA-FMT group, as clustered and visualized using heatmap (Fig. 4A,B). Notably, glutamic acid was negatively correlated with *Bacteroides_caecimuris* in both AA-FMT vs YY-FMT and YA-FMT vs AA-FMT groups, which was further shown by Spearman’s correlation analysis (Fig. 4C,D). These data suggest that increments of *Bacteroides_caecimuris* in aged mice might reduce the glutamic acid level, and transplantation of young gut microbiota could alleviate this defect.

### Increased abundance of *Bacteroides_caecimuris* in young mice reduces the glutamic acid levels in ovary and compromise the oocyte quality

To determine the direct relationship between the abundance of *Bacteroides_caecimuris* and the level of glutamic acid, *Bacteroides_caecimuris* was supplemented to young mice by gavage. Metagenome sequencing confirmed the elevated abundance of *Bacteroides_caecimuris* after gavage compared to the controls (Fig. 4E). Importantly, targeted metabolome data showed that glutamic acid levels in the ovary were accordingly reduced in *Bacteroides_caecimuris*-supplemented young mice (Fig. 4F). The negative correlation between them was further verified by Spearman’s correlation analysis (Fig. 4G). Moreover, genomic analysis by EnsemblBacteria revealed that *Bacteroides_caecimuris* encodes glutamate decarboxylase (GAD), a key enzyme that catalyzes the conversion of glutamic acid to γ-aminobutyric acid (GABA). Immunoblotting data displayed that GAD1 was expressed in both *Bacteroides_caecimuris* and mouse ovary (Fig. 4H). In particular, GAD1 protein levels in mouse ovaries were markedly increased following *Bacteroides_caecimuris* administration (Fig. 4I,J), suggesting that *Bacteroides_caecimuris* might promote glutamic acid consumption through GAD1-mediated metabolism, thereby contributing to the reduction of ovarian glutamic acid levels.

**Figure EV5:**
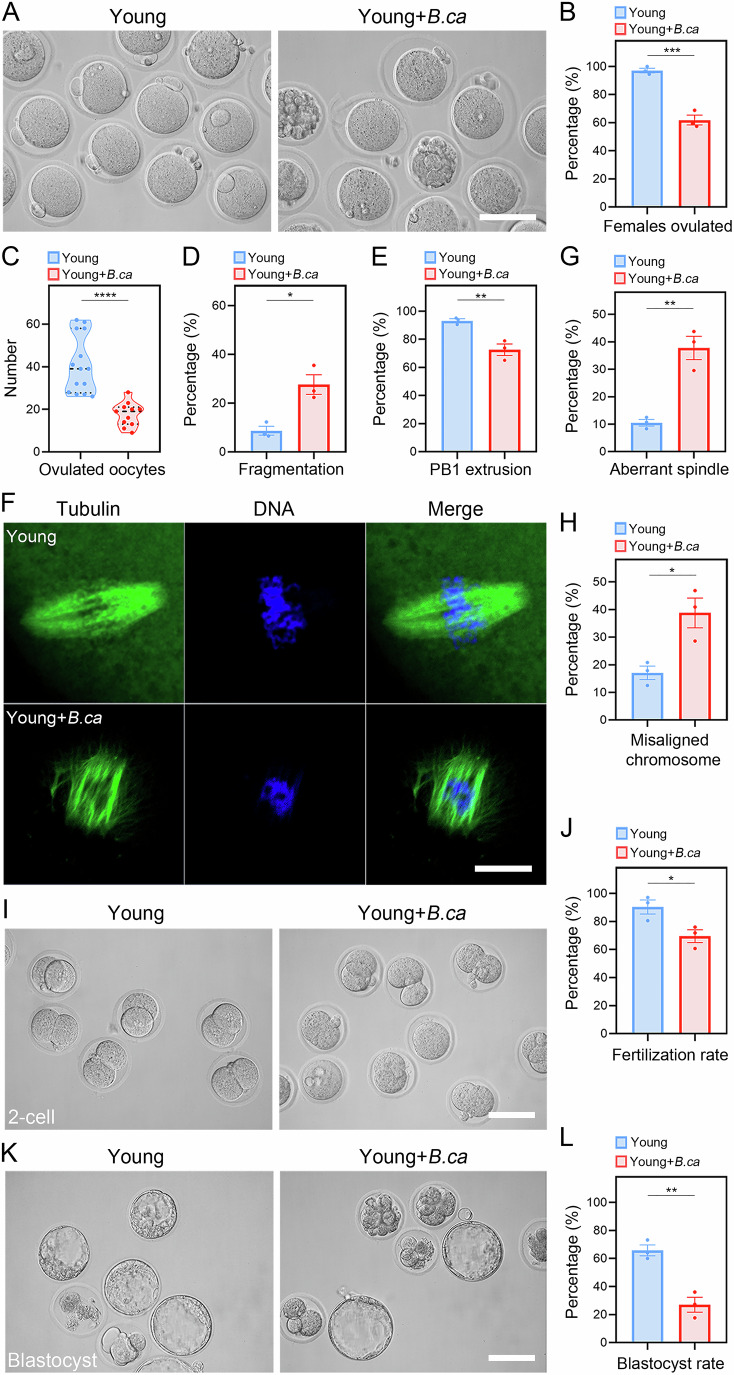
Effects of *Bacteroides_caecimuris* supplementation in young mice on the oocyte quality. (**A**) Representative images of ovulated oocytes collected from young and young+*B.ca* mice. Scale bar, 80 μm. (**B**) The percentage of female mice that were ovulated in young (*n* = 86) and young+*B.ca* (*n* = 118) group. ***P = 0.0008. (**C**) The number of ovulated oocytes was counted in young (*n* = 14) and young+*B.ca* (*n* = 11) mice. ****P < 0.0001. (**D**) The proportion of fragmented oocytes was quantified in young (*n* = 575) and young+*B.ca* (*n* = 194) mice. *P = 0.0126. (**E**) The rate of PB1 extrusion was quantified in young (*n* = 575) and young+*B.ca* (*n* = 194) oocytes. **P = 0.0087. (**F**) Representative images of the spindle morphology and chromosome alignment in young and young+*B.ca* oocytes at M II stage. Scale bar, 20 μm. (**G**) The rate of aberrant spindles was quantified in young (*n* = 76) and young+*B.ca* (*n* = 111) oocytes at M II stage. **P = 0.0035. (**H**) The rate of misaligned chromosomes was quantified in young (*n* = 76) and young+*B.ca* (*n* = 111) at M II stage. *P = 0.0213. (**I**) Representative images of 2-cell embryos in young and young+*B.ca* groups. Scale bar, 80 μm. (**J**) The fertilization rate was quantified in young (*n* = 101) and young+*B.ca* (*n* = 85) groups. *P = 0.0373. (**K**) Representative images of blastocysts in young and young+*B.ca* groups. Scale bar, 80 μm. (**L**) The blastocyst formation rate was quantified in young (*n* = 115) and young+*B.ca* (*n* = 84) groups. **P = 0.0041. Data in (**B**), (**C**), (**D**), (**E**), (**G**), (**H**), (**J**), and (**L**) were presented as mean ± SEM or SD of at least three independent experiments. *P < 0.05; **P < 0.01; ***P < 0.001; ****P < 0.0001. Statistical significance was determined by two-tailed unpaired t-test.

We then assessed the oocyte quality in young mice supplemented with *Bacteroides_caecimuris*. The observations revealed that supplementation of *Bacteroides_caecimuris* perturbed the ovulation ability of female mice, decreased the number of ovulated oocytes, promoted the oocyte fragmentation, and hindered the oocyte maturation, accompanied by the impaired spindle assembly and chromosome alignment (Fig. EV5A–H). Also, supplementation of *Bacteroides_caecimuris* in young mice weakened the fertilization ability of oocytes, and subsequent embryonic development to blastocysts (Fig. EV5I–L). Collectively, our data indicate that *Bacteroides_caecimuris* negatively regulates glutamic acid levels in the ovary, and adversely affects the oocyte quality.

### Young gut microbiota changes the transcriptome profiling of aged oocytes

**Figure EV6:**
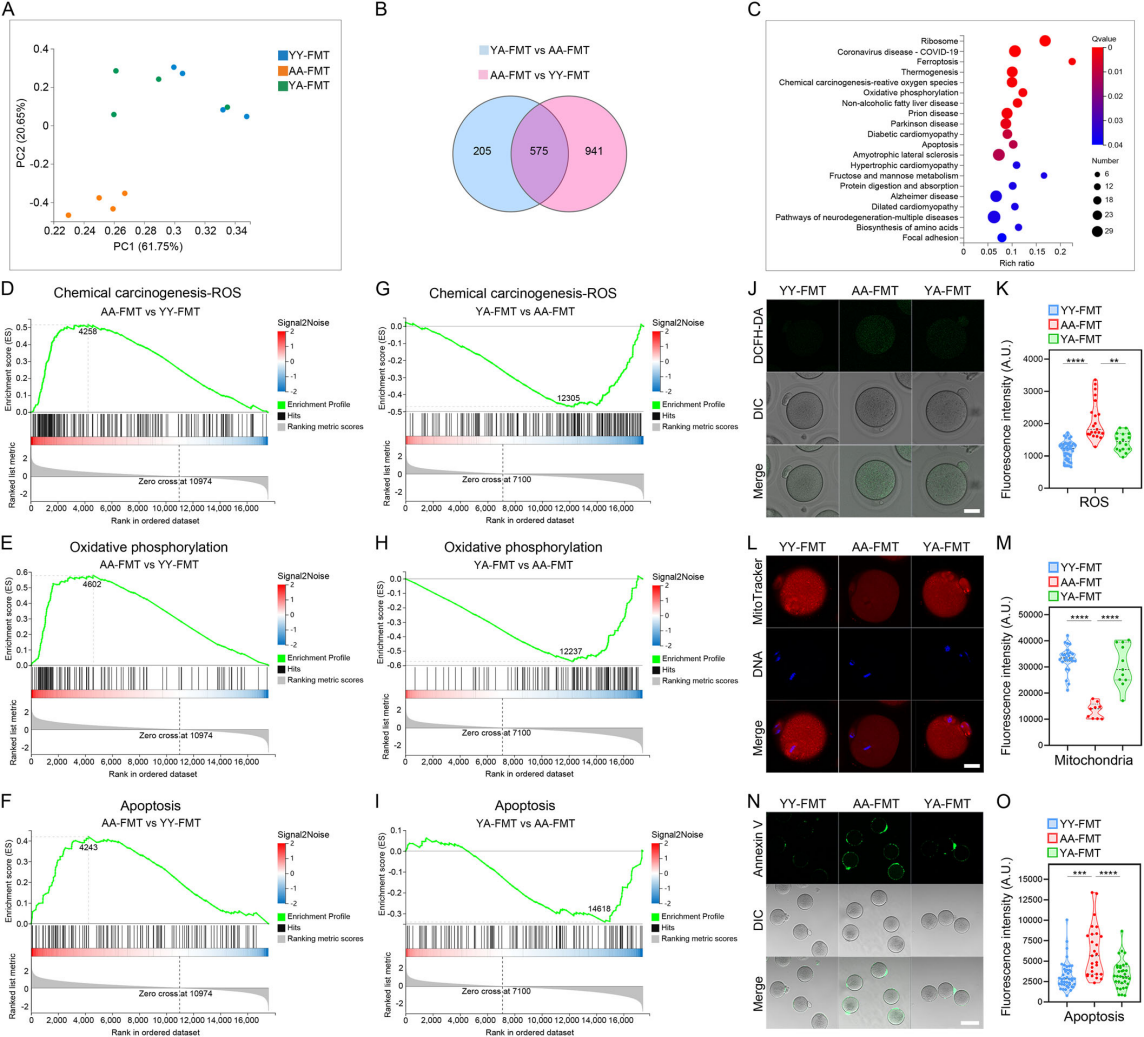
Supplementary transcriptome data related to Fig. 5. (**A**) PCA plot displayed the oocyte samples from YY-FMT, AA-FMT, and YA-FMT mice. Blue circles represented samples from YY-FMT group, orange circles represented samples from AA-FMT group, and green circles represented samples from YA-FMT group. (**B**) Venn diagram showed the number of overlapping differential transcripts between AA-FMT compared to YY-FMT group and YA-FMT compared to AA-FMT group. (**C**) KEGG enrichment analysis of overlapping differential transcripts between AA-FMT compared to YY-FMT group and YA-FMT compared to AA-FMT group. (**D**) GSEA plot showed genes involved in the ROS pathway (AA-FMT positively correlated; YY-FMT negatively correlated). (**E**) GSEA plot showed genes involved in the oxidative phosphorylation pathway (AA-FMT positively correlated; YY-FMT negatively correlated). (**F**) GSEA plot showed genes involved in the apoptosis pathway (AA-FMT positively correlated; YY-FMT negatively correlated). (**G**) GSEA plot showed genes involved in the ROS pathway (YA-FMT positively correlated; AA-FMT negatively correlated). (**H**) GSEA plot showed genes involved in the oxidative phosphorylation pathway (YA-FMT positively correlated; AA-FMT negatively correlated). (**I**) GSEA plot showed genes involved in the apoptosis pathway (YA-FMT positively correlated; AA-FMT negatively correlated). (**J**) Representative images of ROS levels as detected by DCFH-DA staining in YY-FMT, AA-FMT, and YA-FMT oocytes. Scale bar, 20 μm. (**K**) The fluorescence intensity of ROS signals was quantified in YY-FMT (*n* = 45), AA-FMT (*n* = 21), and YA-FMT (*n* = 16) oocytes. ****P < 0.0001, **P = 0.0034. (**L**) Representative images of mitochondria as stained with MitoTracker Red in YY-FMT, AA-FMT, and YA-FMT oocytes. Scale bar, 20 μm. (**M**) The fluorescence intensity of mitochondrial signals was measured in YY-FMT (*n* = 26), AA-FMT (*n* = 9), and YA-FMT (*n* = 11) oocytes. ****P < 0.0001, ****P < 0.0001. (**N**) Representative images of apoptotic oocytes as stained with Annexin-V in YY-FMT, AA-FMT, and YA-FMT groups. Scale bar, 80 μm. (**O**) The fluorescence intensity of Annexin-V signals on the membrane was quantified in YY-FMT (*n* = 42), AA-FMT (*n* = 27), and YA-FMT (*n* = 33) oocytes. ***P = 0.0003, ****P < 0.0001. Data in (**K**), (**M**), and (**O**) were presented as mean ± SD of at least three independent experiments. **P < 0.01; ***P < 0.001; ****P < 0.0001. Statistical significance was determined by two-tailed unpaired t-test.

**Figure 5:**
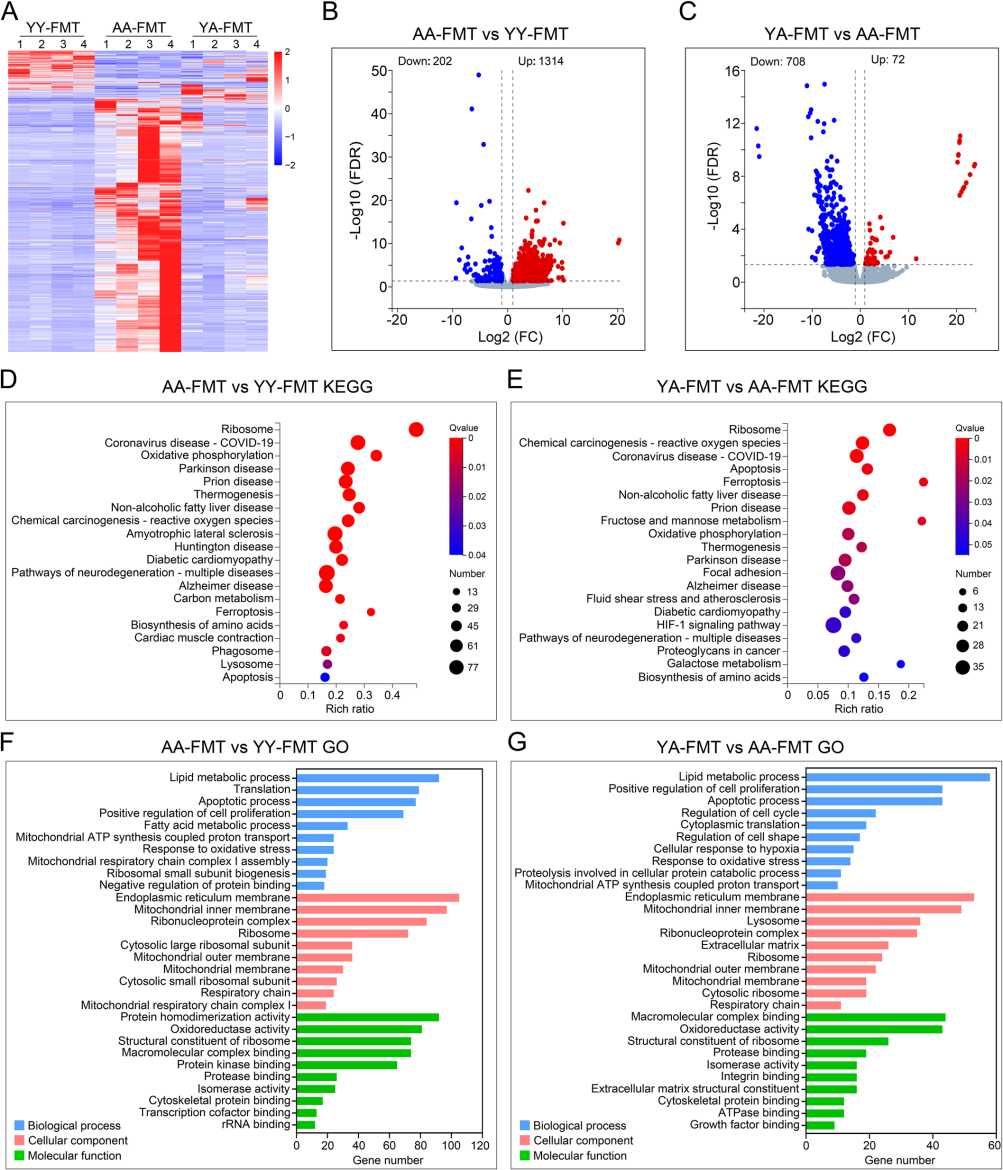
Effects of young gut microbiota on the transcriptome profiling of oocytes in aged mice. (**A**) Total differential transcripts were presented by heatmap illustration in YY-FMT, AA-FMT, and YA-FMT oocytes. (**B**) Upregulated and downregulated differential transcripts were shown by volcano plot in AA-FMT (*n* = 4, replicates) oocytes compared to YY-FMT (*n* = 4, replicates) ones. (**C**) Upregulated and downregulated differential transcripts were shown by volcano plot in YA-FMT (*n* = 4, replicates) oocytes compared to AA-FMT (*n* = 4, replicates) ones. (**D**) KEGG enrichment analysis of differential transcripts in AA-FMT oocytes compared to YY-FMT ones. (**E**) KEGG enrichment analysis of differential transcripts in YA-FMT oocytes compared to AA-FMT ones. (**F**) GO enrichment analysis of differential transcripts in AA-FMT oocytes compared to YY-FMT ones. (**G**) GO enrichment analysis of differential transcripts in YA-FMT oocytes compared to AA-FMT ones. Statistical significance was determined by two-tailed unpaired t-test (**B**, **C**) and the hypergeometric test (**D**, **E**). Source data are available in Dataset EV5.

To gain insights into the molecular mechanisms underlying the improvements of aged oocytes by young gut microbiota, micro-transcriptome was performed by SMART-seq2 to show the changes of transcript levels. PCA plot revealed that oocyte samples from YY-FMT mice clustered closely with those from YA-FMT mice, but far from those from AA-FMT mice (Fig. EV6A). Hierarchically-clustered heatmap analysis of differential transcripts presented the substantial distinct transcriptomic profiles between young and aged oocytes, but partially recovered in YA-FMT oocytes (Fig. 5A). In addition, volcano plots showed 202 downregulated and 1314 upregulated transcripts in aged oocytes compared to the young controls (Fig. 5B), as well as 708 downregulated and 72 upregulated transcripts in YA-FMT oocytes compared to the aged ones (Fig. 5C). Among, 575 differential transcripts were overlapped between AA-FMT vs YY-FMT and YA-FMT vs AA-FMT groups as shown by venn diagram (Fig. EV6B). Furthermore, KEGG analyses indicated that differential transcripts were enriched in the pathways such as reactive oxygen species (ROS), oxidative phosphorylation, and apoptosis in aged oocytes compared to the young ones, which was restored by young gut microbiota (Figs. 5D,E and EV6C). Also, gene set enrichment analysis (GSEA) manifested that the core gene sets enriched in these three pathways were upregulated in AA-FMT group but downregulated in YA-FMT (Fig. EV6D–I). Moreover, gene ontology (GO) analyses in both aged oocytes compared to young ones and YA-FMT oocytes compared to aged ones revealed that differential transcripts were enriched in the biological processes including apoptosis, oxidative stress, and mitochondrial organization (Fig. 5F,G). By fluorescence staining, we further confirmed that ROS levels were elevated in AA-FMT oocytes, accompanied by the damaged mitochondria and occurrence of apoptosis (Fig. EV6J–O). While FMT from young donors eliminated excessive ROS, promoted the mitochondrial integrity, and suppressed the apoptosis in aged oocytes (Fig. EV6J–O). Taken together, from the transcriptome and cellular data, we propose that young gut microbiota might strengthen the mitochondrial function and reduce the ROS levels in aged oocytes to ameliorate their quality.

### Targeted metabolome validates the reduction of glutamic acid levels in ovaries from aged mice

**Figure 6:**
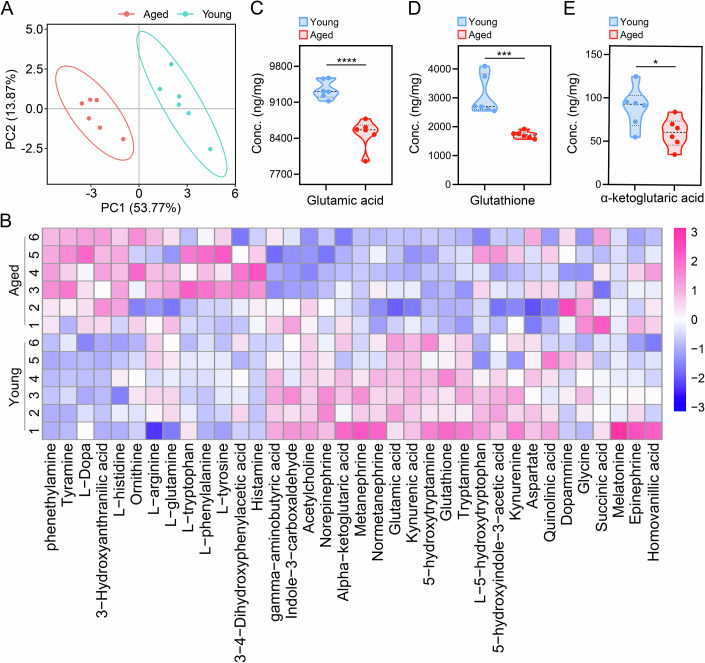
Targeted metabolome for neurotransmitters in mouse ovaries. (**A**) PLS-DA plot showing the samples from young and aged ovaries. Blue circles represented samples from young group, and red circles represented samples from aged group. (**B**) Heatmap illustration displayed the differential abundance of 36 neurotransmitter metabolites in young and aged ovaries. (**C**) The levels of glutamic acid as detected by targeted metabolome in young and aged ovaries. ****P < 0.0001. (**D**) The levels of glutathione as detected by targeted metabolome in young and aged ovaries. ***P = 0.0007. (**E**) The levels of α-ketoglutaric acid as detected by targeted metabolome in young and aged ovaries. *P = 0.0365. Data in (**C**), (**D**), and (**E**) were presented as mean ± SD. *P < 0.05, ***P < 0.0001. Statistical significance was determined by two-tailed unpaired t-test. Source data are available online for this figure.

**Figure EV7:**
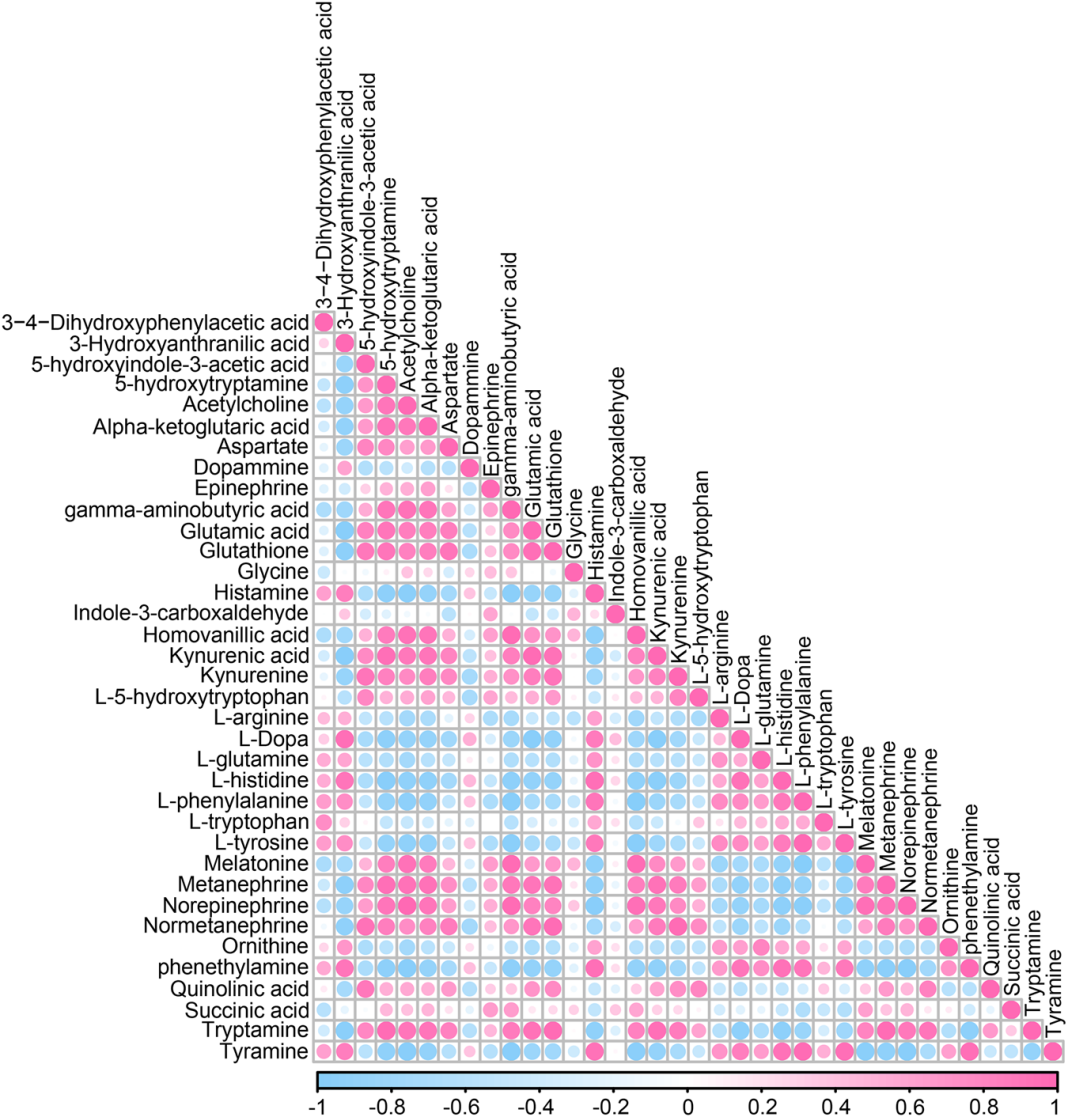
Spearman’s correlation analysis of neurotransmitter metabolites. Red represented positive correlation, and blue represented negative correlation. The darker the color, the stronger the correlation.

To ask whether glutamic acid levels were also changed in ovaries from aged mice, we carried out targeted metabolome for neurotransmitters. PLS-DA analysis showed that sample replicates clustered together in each group but separated between groups, indicating that detected metabolites were dramatically altered in aged ovaries (Fig. 6A). This was further confirmed by the clustered heatmap analysis displaying that a total of 36 metabolites were changed in varying degrees in aged ovaries compared with young controls (Fig. 6B). Of note, the level of glutamic acid was significantly decreased in aged ovaries as shown by the bar graphs derived from metabolome data (Fig. 6C). In addition, combined with the correlation analysis among metabolites (Fig. EV7), we found that the levels of glutathione and α-ketoglutaric acid, two glutamic acid highly-correlated metabolites, were also substantially reduced in aged ovaries (Fig. 6D,E). These observations reflect that maternal aging reduces the levels of some powerful antioxidants such as glutathione and α-ketoglutaric acid, as well as their synthetic precursor glutamic acid in ovaries.

### Supplementation of glutamic acid elevates the quantity and quality of oocytes from aged mice

**Figure 7:**
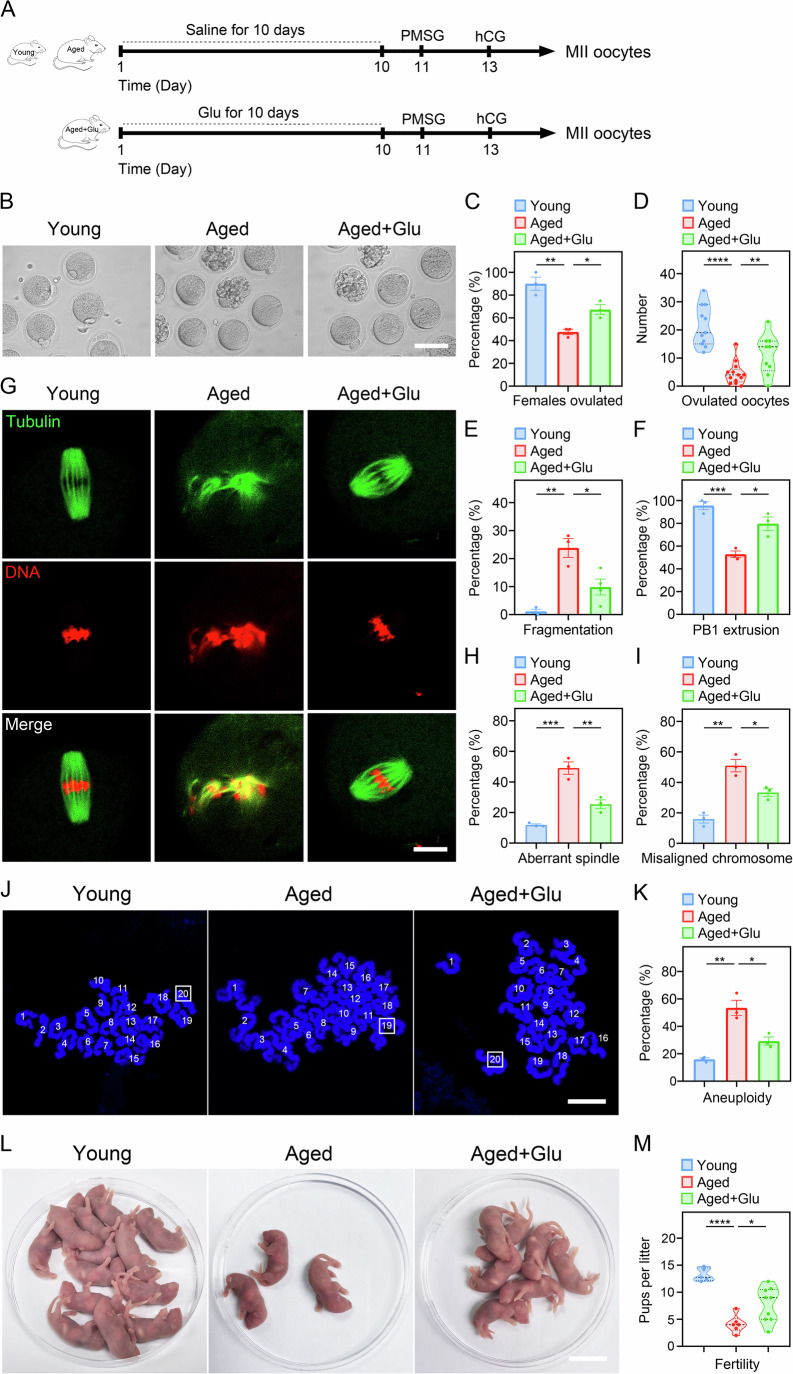
Effects of glutamic acid supplementation on the quality of aged oocytes. (**A**) A timeline scheme for glutamic acid supplementation, hormone injection and subsequent analyses. Aged mice were orally gavaged with 300 μl glutamic acid (Sigma-Aldrich; 100 mg/kg body weight per day, dissolved in saline) or the equivalent volume of saline for 10 consecutive days, followed by hormone injection and oocyte collection. Young mice were orally gavaged with the saline as the control. (**B**) Representative images of ovulated oocytes collected from young, aged, and aged+Glu mice. Scale bar, 60 μm. (**C**) The percentage of female mice that were ovulated in young (*n* = 25), aged (*n* = 32), and aged+Glu (*n* = 23) group. **P = 0.0025, *P = 0.0167. (**D**) The number of ovulated oocytes was counted in young (*n* = 11), aged (*n* = 13), and aged+Glu (*n* = 9) mice. ****P < 0.0001, **P = 0.0096. (**E**) The proportion of fragmented oocytes was quantified in young (*n* = 310), aged (*n* = 123), and aged+Glu (*n* = 123) mice. **P = 0.0027, *P = 0.0243. (**F**) The rate of PB1 extrusion was quantified in young (*n* = 285), aged (*n* = 90), and aged+Glu (*n* = 109) oocytes. ***P = 0.0008, *P = 0.0151. (**G**) Representative images of the spindle morphology and chromosome alignment in young, aged, and aged+Glu oocytes at MII stage. Scale bar, 20 μm. (**H**) The rate of aberrant spindles was quantified in young (*n* = 51), aged (*n* = 49), and aged+Glu (*n* = 118) oocytes at MII stage. ***P = 0.0008, **P = 0.0089. (**I**) The rate of misaligned chromosomes was quantified in young (*n* = 51), aged (*n* = 49), and aged+Glu (*n* = 118) oocytes at MII stage. **P = 0.0019, *P = 0.0204. (**J**) Representative images of chromosome spreads in MII oocytes from young, aged, and aged+Glu mice. Scale bar, 5 μm. (**K**) The rate of aneuploidy was quantified in young (*n*  =  82), aged (*n*  =  45), and aged+Glu (*n*  =  62) oocytes at MII stage. **P = 0.0026, *P = 0.0181. (**L**) Representative images of pups delivered by young, aged, and aged+Glu female mice. Scale bar, 2 cm. (**M**) The average pups per litter were counted in in young (*n* = 5), aged (*n* = 6), and aged+Glu (*n* = 9) groups. ****P < 0.0001, *P = 0.0246. Data in (**C**), (**D**), (**E**), (**F**), (**H**), (**I**), (**K**), and (**M**) were presented as mean ± SEM or SD of at least three independent experiments. *P < 0.05; **P < 0.01; ***P < 0.001; ****P < 0.0001. Statistical significance was determined by two-tailed unpaired t-test. Source data are available online for this figure.

**Figure EV8:**
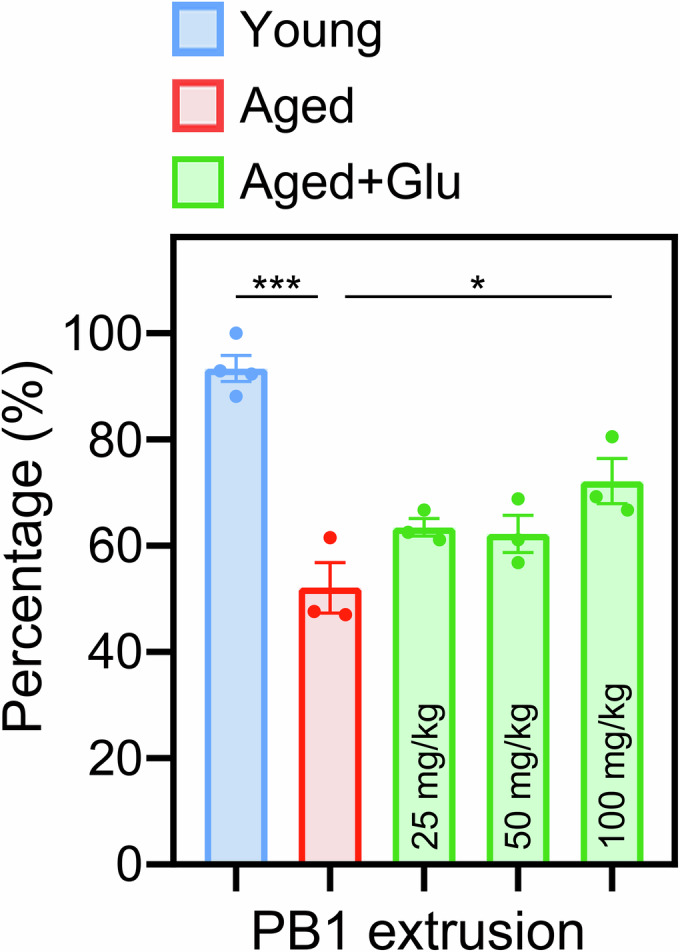
Effects of different concentrations of glutamic acid on the maturation of aged oocytes. The percentage of PB1 extrusion was quantified in young (*n* = 222), aged (*n* = 51), and aged+Glu (25 mg/kg: *n* = 38, 50 mg/kg: *n* = 71, 100 mg/kg: *n* = 88) oocytes. ***P = 0.0004, *P = 0.0344. Data were presented as mean ± SEM of at least three independent experiments. *P < 0.05; ***P < 0.001. Statistical significance was determined by two-tailed unpaired t-test.

**Figure EV9:**
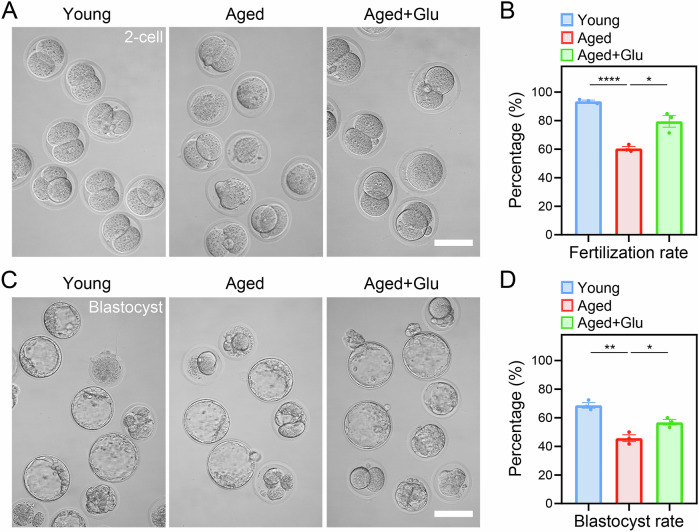
Effects of glutamic acid supplementation on the fertilization and early embryonic development in aged mice. (**A**) Representative images of 2-cell embryos in young, aged, and aged+Glu groups. Scale bar, 80 μm. (**B**) The fertilization rate was quantified in young (*n* = 128), aged (*n* = 96), and aged+Glu (*n* = 78) groups. ****P < 0.0001, *P = 0.0120. (**C**) Representative images of blastocysts in young, aged, and aged+Glu groups. Scale bar, 80 μm. (**D**) The blastocyst formation rate was quantified in young (*n* = 143), aged (*n* = 58), and aged+Glu (*n* = 48) groups. **P = 0.0018, *P = 0.0227. Data in (**B**) and (**D**) were presented as mean ± SEM of at least three independent experiments. *P < 0.05; **P < 0.01; ****P < 0.0001. Statistical significance was determined by two-tailed unpaired t-test.

Given that above multi-omics data have indicated that glutamic acid might mediate the restorative effects of young gut microbiota on the aged oocyte quality, we then tested whether direct supplementation of glutamic acid would rejuvenate the oocytes from aged mice. Oocytes were retrieved to count the number and assess the morphology after hormone priming (Fig. 7A). We observed that the number of females ovulated, the number of ovulated oocytes, and the oocyte maturational rate as judged by PBE were all remarkably reduced in aged mice compared to the young ones, but the occurrence of fragmentation was considerably increased (Figs. 7B–F and EV8). Whereas supplementation of glutamic acid enhanced the quantity and quality of aged oocytes (Figs. 7B–F and EV8). In addition, the spindle/chromosome structure was further examined in aged oocytes supplemented with glutamic acid. By immunostaining and confocal imaging, we found that a large number of morphology-aberrant spindles with misaligned chromosomes were present in aged oocytes compared to the young controls, but partially rescued by glutamic acid supplementation (Fig. 7G–I). Consistently, the high incidence of aneuploidy occurring in aged oocytes was also lowered by supplementation of glutamic acid (Fig. 7J,K). We subsequently observed the fertilization and early embryonic development, and demonstrated that glutamic acid supplementation largely recovered the compromised fertilization rate and blastocyst formation rate caused by aging (Fig. EV9). Accordingly, the fertility of aged female mice was improved by glutamic acid supplementation as well, as evaluated by counting the average number of pups produced per litter (Fig. 7L,M). To sum up, these findings reveal that supplementation of glutamic acid can ameliorate the oocyte quality and fertility of aged female mice.

### The impact of glutamic acid on the aged oocytes depends on the mitochondrial function-mediated redox homeostasis

**Figure 8:**
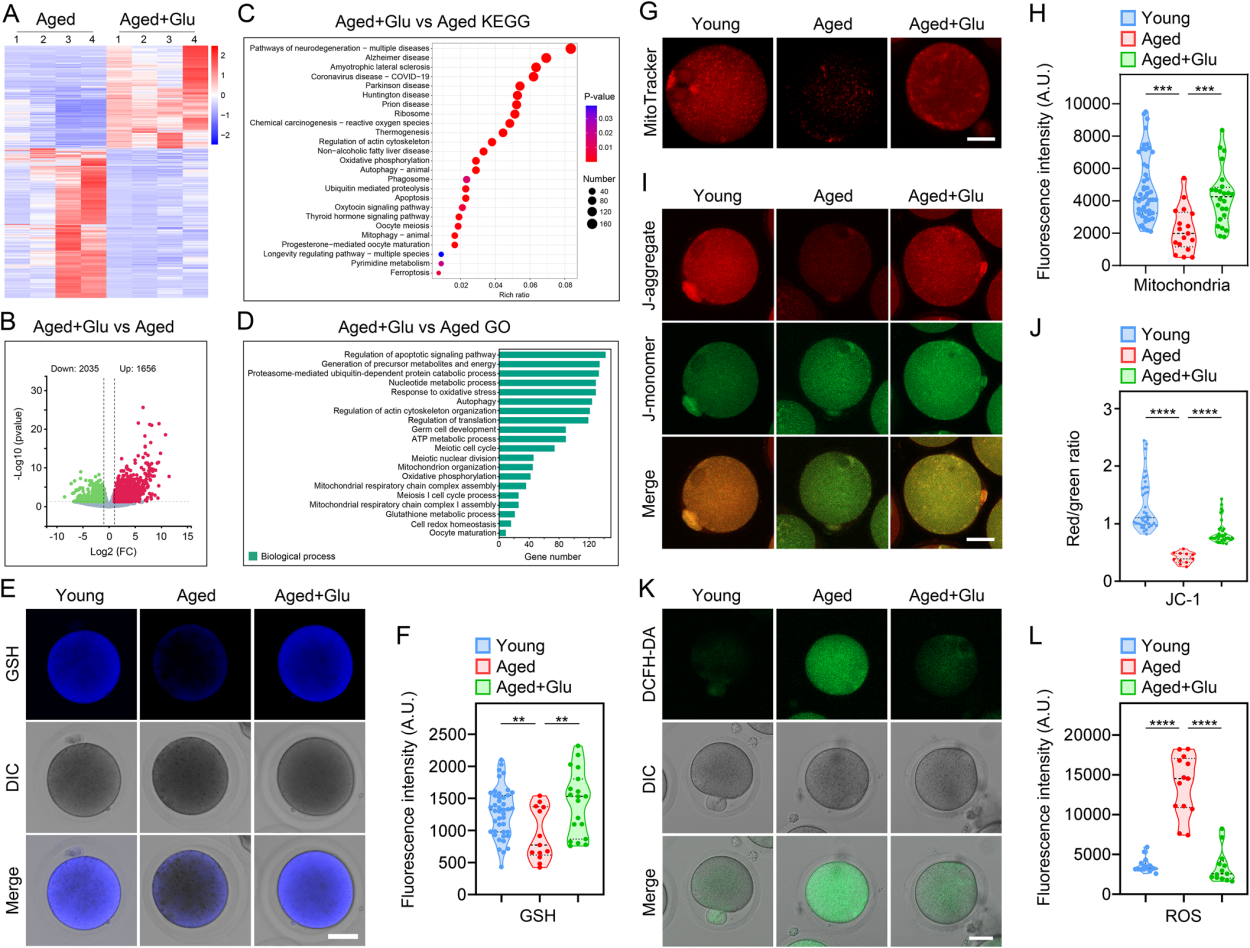
Effects of glutamic acid supplementation on the mitochondrial dynamics and ROS levels in aged oocytes. (**A**) Total differential transcripts were presented by heatmap illustration in aged and aged+Glu oocytes. (**B**) Upregulated and downregulated differential transcripts were shown by volcano plot in aged+Glu (*n* = 4, replicates) oocytes compared to aged (*n* = 4, replicates) ones. (**C**) KEGG enrichment analysis of differential transcripts in aged+Glu oocytes compared to aged ones. (**D**) GO enrichment analysis of differential transcripts in aged+Glu oocytes compared to aged ones. (**E**) Representative images of GSH levels as detected by mBBr staining in young, aged, and aged+Glu oocytes. Scale bar, 20 μm. (**F**) The fluorescence intensity of GSH signals was measured in young (*n* = 43), aged (*n* = 13), and aged+Glu (*n* = 19). **P < 0.0063, **P = 0.0067. (**G**) Representative images of mitochondria as stained with MitoTracker Red in young, aged, and aged+Glu oocytes. Scale bar, 20 μm. (**H**) The fluorescence intensity of mitochondrial signals was measured in young (*n* = 48), aged (*n* = 17), and aged+Glu (*n* = 24) oocytes. ***P = 0.0002, ***P = 0.0002. (**I**) Representative images of mitochondrial membrane potential as detected by JC-1 staining in young, aged, and aged+Glu oocytes. Scale bar, 20 μm. (**J**) The ratio of J-aggregate (red) to J-monomer (green) was quantified in young (*n* = 40), aged (*n* = 11), and aged+Glu (*n* = 38) oocytes. ****P < 0.0001, ***P < 0.0001. (**K**) Representative images of ROS levels as detected by DCFH-DA staining in young, aged, and aged+Glu oocytes. Scale bar, 20 μm. (**L**) The fluorescence intensity of ROS signals was quantified in young (*n* = 15), aged (*n* = 13), and aged+Glu (*n* = 13) oocytes. ****P < 0.0001, ***P < 0.0001. Data in (**F**), (**H**), (**J**), and (**L**) were presented as mean ± SD of at least three independent experiments. **P < 0.01; ***P < 0.001; ****P < 0.0001. Statistical significance was determined by two-tailed unpaired t-test (**B**, **F**, **H**, **J**, **L**). Source data are available online for this figure.

**Figure EV10:**
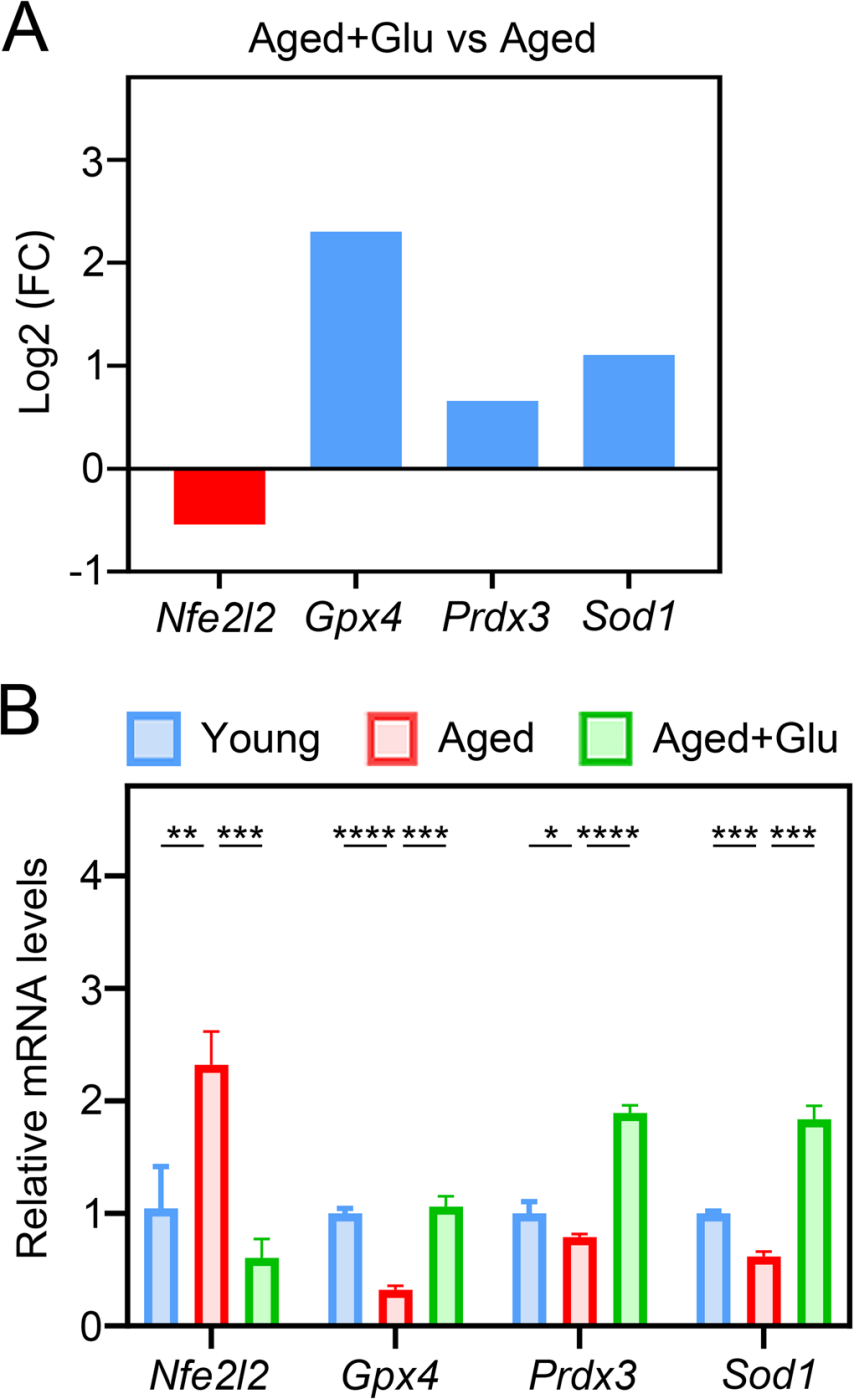
Effects of glutamic acid supplementation on the transcript levels of some antioxidant genes. (**A**) RNA-seq results of selected antioxidant genes in aged+Glu oocytes compared to aged ones. (**B**) Validation of RNA-seq data by quantitative RT-PCR in young (*n* = 60), aged (*n* = 60), and aged+Glu (*n* = 60) oocytes. **P = 0.0065, ***P = 0.0009, ****P < 0.0001, ***P = 0.0002, *P = 0.0131, ****P < 0.0001, ***P = 0.0002, ***P = 0.0002. Data in (**B**) were presented as mean ± SEM of at least three independent experiments. *P < 0.05; **P < 0.01; ***P < 0.001; ****P < 0.0001. Statistical significance was determined by multiple t-test.

To elucidate how glutamic acid rejuvenates the aged oocytes, micro-transcriptome was carried out to compare the differential transcripts between aged and glutamic acid-supplemented oocytes. Hierarchically-clustered heatmap analysis of differential transcripts displayed that glutamic acids considerably changed the transcript profiles of aged oocytes, leading to 2035 downregulated and 1656 upregulated transcripts in glutamic acid-supplemented oocytes compared to the aged ones as shown in volcano plot (Fig. 8A,B). KEGG and GO analyses revealed that pathways or biological processed related to oocyte meiosis, cell cycle process, cytoskeleton organization, nuclear division, and longevity regulation were affected by the glutamic acid supplementation (Fig. 8C,D), which is consistent with the above results that supplementation of glutamic acid alleviated the meiotic maturational arrest, spindle/chromosome abnormalities, and aneuploidy observed in aged oocytes. In addition, differential transcripts were also enriched in the pathways or biological processes associated with ROS, response to oxidative stress, cell redox homeostasis, glutathione metabolic process, oxidative phosphorylation, mitophagy, and mitochondrial organization (Fig. 8C,D), indicating that glutamic acid supplementation is involved in the regulation of antioxidant capacity and mitochondrial function in aged oocytes. Accordingly, RNA-seq analysis followed by quantitative reverse transcription (qRT)-PCR validation presented that glutamic acid supplementation restored the transcript levels of several key antioxidant genes in aged oocytes (Fig. EV10). Monobromobimane (mBBr) staining further demonstrated a significant increase in intracellular glutathione levels after treatment (Fig. 8E,F). Meanwhile, Mitotracker and JC-1 staining showed that glutamic acid supplementation restored the abundance of mitochondria and strengthened their function (Fig. 8G–J). Furthermore, elevated levels of ROS present in aged oocytes were markedly reduced following glutamic acid supplementation, as evaluated by DCFH-DA staining (Fig. 8K,L). Collectively, our data validate the favorable impacts of glutamic acid on the aged oocytes *via* maintaining the mitochondrial function-mediated redox homeostasis.

### The enhancement of oocyte quality by glutamic acid is conserved in pigs

**Figure EV11:**
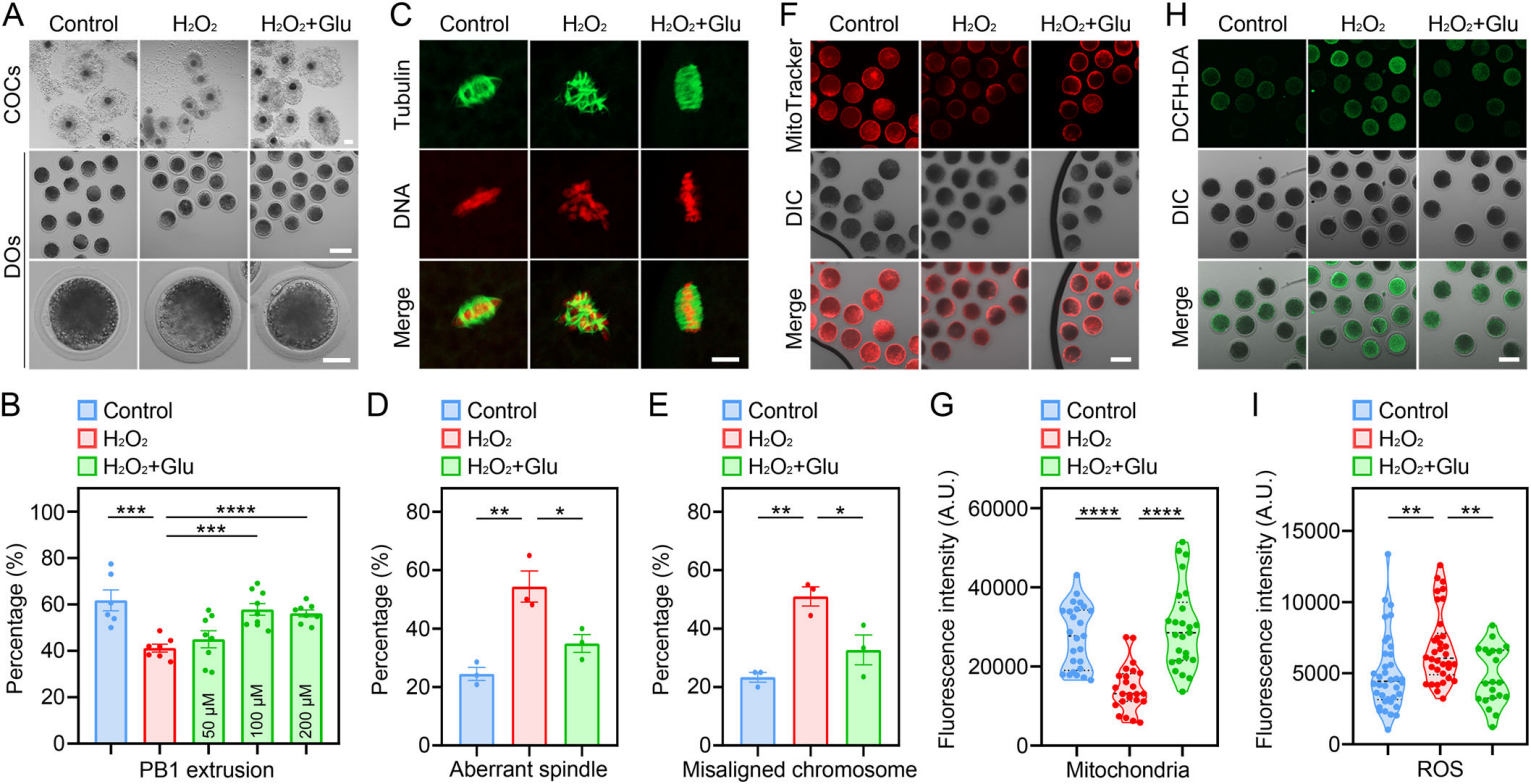
Effects of glutamic acid supplementation on the quality of porcine oocytes treated with H_2_O_2_. (**A**) Representative images of in vitro matured porcine oocytes in control, H_2_O_2_-treated, and Glu-supplemented groups. Scale bars, 120 μm, 120 μm, 30 μm. GV oocytes were treated with 100 μM H_2_O_2_ for 45 min and then cultured in the fresh medium with or without glutamic acid for in vitro maturation. (**B**) The rate of PB1 extrusion was recorded in control (*n* = 216), H_2_O_2_-treated (*n* = 351), and different concentrations of Glu-supplemented groups (50 μM: *n* = 365, 100 μM: *n* = 339; 200 μM: *n* = 302) after in vitro culture for 44 h. 200 μM Glu was used for subsequent analyses. ***P = 0.0009, ***P = 0.0002, ****P < 0.0001. (**C**) Representative images of spindle morphology and chromosome alignment in control, H_2_O_2_-treated, and Glu-supplemented oocytes. Scale bar, 5 μm. (**D**) The rate of abnormal spindles was measured in control (*n* = 77), H_2_O_2_-treated (*n* = 75), and Glu-supplemented (*n* = 49) oocytes. **P = 0.0067, *P = 0.0340. (**E**) The rate of misaligned chromosomes was measured in control (*n* = 77), H_2_O_2_-treated (*n* = 75), and Glu-supplemented (*n* = 49) oocytes. **P = 0.0017, *P = 0.0395. (**F**) Representative images of mitochondrial signals as shown by MitoTracker staining in control, H_2_O_2_-treated, and Glu-supplemented oocytes. Scale bar, 120 μm. (**G**) The fluorescence intensity of mitochondrial signals was measured in control (*n* = 25), H_2_O_2_-treated (*n* = 24), and Glu-supplemented (*n* = 25) oocytes. ****P < 0.0001, ****P < 0.0001. (**H**) Representative images of ROS levels as detected by DCFH-DA staining in control, H_2_O_2_-treated, and Glu-supplemented oocytes. Scale bar, 120 μm. (**I**) The fluorescence intensity of ROS signals was measured in control (*n* = 38), H_2_O_2_-treated (*n* = 34), and Glu-supplemented (*n* = 21) oocytes. **P = 0.0033, **P = 0.0034. Data in (**B**), (**D**), (**E**), (**G**), and (**I**) were presented as mean ± SEM or SD of at least three independent experiments. *P < 0.05; **P < 0.01; ***P < 0.001; ****P < 0.0001. Statistical significance was determined by two-tailed unpaired t-test.

Porcine oocytes were further applied to test the beneficial influences of glutamic acid on the oocyte quality in another species. The in vitro maturation of porcine oocytes showed that PBE rate was significantly lower in oxidative stress-induced oocytes treated with H_2_O_2_ than that in controls, but increased in glutamic acid-supplemented ones (Fig. EV11A,B). In addition, we also observed that glutamic acid supplementation restored the spindle/chromosome structure impaired by H_2_O_2_ treatment in porcine oocytes (Fig. EV11C–E). In the meantime, glutamic acid supplementation relieved the mitochondrial defects in H_2_O_2_-treated oocytes (Fig. EV11F,G), and hence reduced the high levels of ROS (Fig. EV11H,I). Thus, we document that the action mechanism of glutamic acid on the oocyte quality is conserved across species.

## Discussion

Gut microbiota has been identified as a complex ecosystem to play vital roles in both health and disease. It forms multidirectional connecting axis with other organs, including the brain, heart, lung, liver, kidney, bone, muscle and reproduction etc., for host-microbe interactions (Afzaal et al, 2022). Therefore, emerging evidence in animal models and humans indicates that gut microbiota dysbiosis is related to a variety of diseases, such as obesity, diabetes, anxiety, depression, hypertension, cardiovascular diseases, inflammatory bowel disease, reproductive disease and cancer (Durack and Lynch, 2019). Notably, recent studies demonstrate that the microbiota is not merely correlated with reproductive dysfunction but can actively regulate female reproductive longevity by shaping follicle dynamics, ovarian fibrosis, and gene expression patterns during critical developmental windows. Microbial colonization and microbiota-derived metabolites have been shown to preserve ovarian reserve and fertility capacity, highlighting the functional importance of the gut-ovary axis (Munyoki et al, 2025). In line with these findings, it has been reported that alterations of gut microbiota composition influence both male and female reproductive system (Xu et al, 2022; Zhang et al, 2022), and our findings further illustrate that reshaping of gut microbiota from young donors by FMT could enhance the quality of female germ cells in aged animals.

Taking advantage of metagenomics, we first of all manifested that transplantation of gut microbiota from young donors to aged recipient mice by FMT remodeled the gut microbiota composition to a young-like pattern, which is consistent with previous reports(Thevaranjan et al, 2017). Subsequent analysis of reproductive phenotypes further illustrated that young gut microbiota enhanced the quality of aged oocytes by promoting ovulation and maturation, as well as suppressing fragmentation of oocytes, thus increasing the female fertility. Interestingly, a recent study documented that FMT from young mice increased the litter size of aged mice *via* improving the immune microenvironment in ovaries (Xu et al, 2022). However, how the remodeling of aging-induced change of gut microbiota by FMT influences the oocyte quality and female fertility is still unknown.

The most important finding in our work is that we elucidated this action mechanism from the metabolic perspective. It was found that glutamic acid level was remarkably decreased in both intestinal digesta and ovaries from aged mice as analyzed by untargeted and targeted metabolomics, and this reduction was highly correlated with the increased abundance of *Bacteroides_caecimuris*. Previous studies have evidenced that enrichment of *Bacteroides_caecimuris* was found in a mouse model of retinitis pigmentosa (Kutsyr et al, 2021), and in humans exposed to pollutant O3 (Fouladi et al, 2020), suggesting a possible adverse impact of this bacteria species on the organism. In addition, a high *Bacteroides* dominance was shown to be associated with the decreased survival in humans over 80 years (Wilmanski et al, 2021), indicative of its abundance with aging. Our results also attested that supplementation of *Bacteroides_caecimuris* in young mice reduced the glutamic acid levels in the ovary and impaired the oocyte quality. This effect may be explained, at least in part, by GAD-mediated glutamic acid metabolism driven by *Bacteroides_caecimuris*, leading to decreased ovarian glutamic acid availability. Glutamic acid is a non-essential amino acid that plays a critical role in biosynthesis of amino acids, nucleic acids, metabolites, neurotransmitters, and antioxidants (Yelamanchi et al, 2016). Thus, as a major metabolic hub in many organisms, glutamic acid metabolism exerts diverse functions in various biological processes (Brosnan and Brosnan, 2013). Especially, glutamic acid can act as a synthetic precursor of glutathione that reduces oxidative stress and maintains redox balance in cells (Asantewaa and Harris, 2021). Glutamic acid is also a precursor of α-ketoglutaric acid, which has shown an improvement in oocyte quality in vitro and in vivo due to its antioxidant properties (Chen et al, 2022; Zhang et al, 2021b). Notably, our targeted metabolome data revealed that both glutathione and α-ketoglutaric acid levels were reduced in aged ovaries. Furthermore, it has been reported that glutamic acid addition extends yeast chronological lifespan (Wu et al, 2013), and recovers the protein synthesis rhythm in hepatocytes from old rats to the level of young rats (Brodsky et al, 2017). Our transcriptomic and cellular data demonstrated that supplementation of glutamic acid in aged mice improved oocyte maturation and reduced the incidence of oocyte fragmentation and spindle/chromosome abnormalities by maintaining the redox homeostasis and mitochondrial integrity.

Despite these findings, several limitations of this study should be noted. Our findings are mainly based on mouse models, and further studies are needed to determine their relevance in other animals and humans. Although we propose that *Bacteroides_caecimuris* regulates ovarian glutamic acid levels through GAD-mediated metabolism, the detailed mechanisms linking gut microbial metabolism to ovarian metabolite homeostasis remain unclear. Moreover, additional microbiota-derived metabolites may also participate in the regulation of oocyte quality and female reproductive aging.

**Figure EV12:**
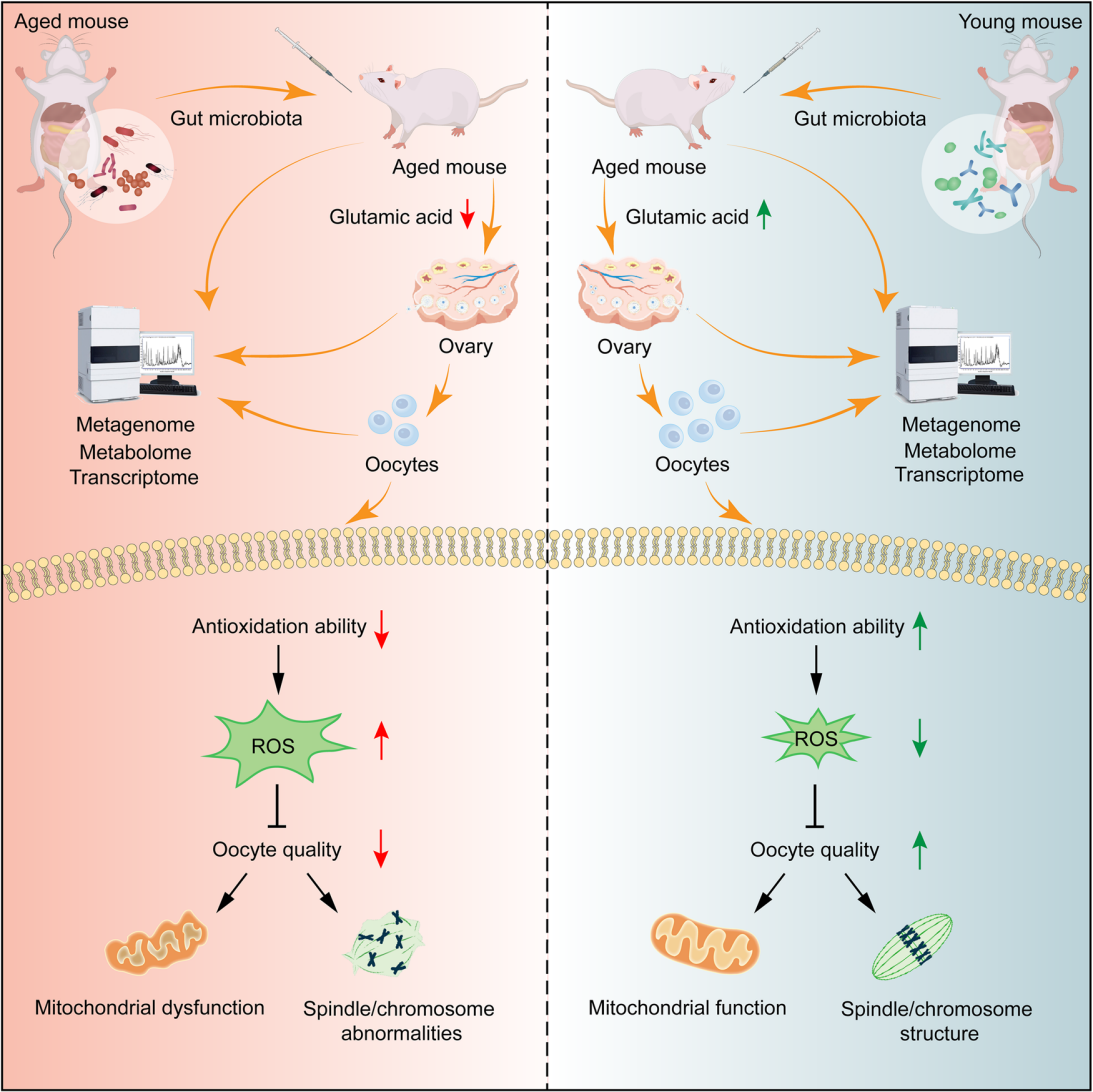
Working model regarding how young gut microbiota rejuvenate the quality of maternally aged oocytes.

In summary, we provided a body of evidence documenting that rebuilding a gut microbiota in aged mice by FMT from young donors is an effective approach to enhance the quality of oocytes and animal fertility deteriorated by maternal aging. In addition, on the basis of the joint analysis of metagenome and metabolome, we further uncovered that glutamic acid is the key metabolite to mediate the effects of FMT, and supplementation of glutamic acid can recover the mitochondrial function to rejuvenate the oocytes from aged animals (Fig. EV12). Collectively, our study develops the potential strategies to improve the reproductive outcome of women of advanced reproductive age.

## Methods

**Table:** Reagents and tools table

Reagent/Resource	Reference or Source	Identifier or Catalog Number
**Experimental models**		
ICR(*M.musculus*)	Yangzhou University	N/A
ICR(*M.musculus*)	Hangzhou Medical College	N/A
Porcine ovaries	Local abattoir	
**Recombinant DNA**		
N/A	N/A	N/A
**Antibodies**		
Anti-α-Tubulin−FITC	Sigma-Aldrich	F2168
Anti-rabbit IgG	Cell Signaling Technology	7076S
Anti-mouse IgG	Cell Signaling Technology	7074S
GAD1 Polyclonal antibody	Proteintech	10408-1-AP
Anti-β-Tubulin Mouse	TransGen Biotech	HC101-01
**Oligonucleotides and other sequence-based reagents**		
PCR primers	This study	Table EV1
**Chemicals, Enzymes and other reagents**		
MitoTracker Red CMXRos	ThermoFisher Scientific	M7512
Bacitracin	Solarbio	B8181
Streptomycin Sulfate	Solarbio	S8290
Neomycin Sulfate	Solarbio	N8090
PBS	Solarbio	P1020
Glutamic acid	Sigma-Aldrich	G8415
Hyaluronidase	Sigma-Aldrich	H3506
PMSG	Ningbo Second Hormone Factory	N/A
hCG	Ningbo Second Hormone Factory	N/A
KSOM Medium	Nanjing Aibei Biotechnology	M1435
M2 Medium	Nanjing Aibei Biotechnology	M1250
MitoProbe JC-1 Assay Kit	ThermoFisher Scientific	M34152
Reactive Oxygen Species Assay Kit	Beyotime	S0033S
mBBr	ATT bioqueset	633
Tyrode’s Solution	Sigma-Aldrich	T1788
LDS Sample Buffer	ThermoFisher Scientific	NP0007
Sample Reducing Agent	ThermoFisher Scientific	NP0009
Triton X-100	Sigma-Aldrich	V900502
TWEEN 20	Sigma-Aldrich	V900548
16% Formaldehyde	Cell Signaling Technology	12606S
Hoechst 33342	ThermoFisher Scientific	H3570
TBST	Solarbio	T1082
Chemistar ECL Western Blotting Substrate	Tanon	180-501
QIAamp PowerFecal Pro DNA Kit	QIAGEN	51804
ChamQ Universal SYBR qPCR Master Mix	Vazyme	Q711
Dynabead mRNA DIREC Micro kit	ThermoFisher Scientific	61011
Glutamate Content Assay Kit	Solarbio	BC5215
HiScript II RT SuperMix for qPCR	Vazyme	R323
Protease Inhibitor Cocktail	Cwbio	CW2200S
Annexin V-FITC Apoptosis Detection Kit	Beyotime	C1062S
TCM199 Medium	ThermoFisher Scientific	11150059
Epidermal Growth Factor	Sigma-Aldrich	E9644
Insulin	Beyotime	P3375
Penicillin-Streptomycin	ThermoFisher Scientific	15140122
SurePAGE™ Bis-Tris 10%	GenScript	M00665
eBlot L2 Transfer Concentrate Kit mini	GenScript	L01015C-75
Primary Antibody Dilution Buffer	Beyotime	P0256
Secondary Antibody Dilution Buffer	Beyotime	P0258
Blocking Buffer	Beyotime	P0252
Universal Total Protein Extraction Kit	Beyotime	P0013WS
Sodium pyruvate ReagentPlus	Sigma-Aldrich	P2256
L-Cysteine anhydrous	Sigma-Aldrich	C7477
**Software**		
GraphPad Prism 8	https://www.graphpad.com	
ImageJ	https://imagej.net	
Photoshop 2021	https://www.adobe.com	
ZEN 2012	https://www.zeiss.com.cn	
Olyvia	https://www.olympuschina.com	
**Other**		
*Bacteroides_caecimuris*	GemPharmatech	GPTB0082
Confocal Laser Scanning Microscope	ZEISS	LSM 900
Confocal Laser Scanning Microscope	OLYMPUS	FV3000

### Animals

ICR mice were obtained from Yangzhou University and Hangzhou Medical College. All mouse protocols and experimental procedures (NJAU.No.20220307033) were approved by the Animal Research Institute Committee of Nanjing Agricultural University, China. The young (6–8 week old) and aged (50–58 week old) ICR female mice were housed in a 12 h light-dark cycle with constant temperature and free access to food and water.

### Antibiotic treatment and FMT procedure

For antibiotic treatment, mice were gavaged daily with the antibiotic mix containing bacitracin, neomycin, and streptomycin (200 mg/kg body weight for each antibiotic) for 3 days (Kennedy et al, 2018; Sayin et al, 2013). For FMT, mice were randomized into the following groups: YY-FMT (FMT from young donors to young recipients), AA-FMT (FMT from aged donors to aged recipients), and YA-FMT (FMT from young donors to aged recipients). Fresh fecal materials were collected from young and aged mice every day, and FMT was carried out *via* oral gavage with a fecal suspension (100 mg/ml) in a volume of 200 μl on days 4–8 and 15 from the beginning of antibiotic regime, with reference to a previously published protocol (D’Amato et al, 2020).

### Metagenomic sequencing

The metagenomic sequencing was conducted using the DNBSEQ platform at Beijing Genomics Institute (BGI) (Shenzhen, China). In brief, 8 replicates of samples for each group (100 mg intestinal digesta per replicate) were collected for DNA extraction. Genomic DNA was randomly fragmented by Covaris and selected by magnet beads to an average size of 200–400 bp. Fragments were then end repaired, 3’ adenylated, and ligated by adapters, followed by PCR amplification. Double-stranded PCR products were heat denatured and circularized by the splint oligo sequence, and the single strand circle DNA were formatted as the final library. The library was amplified with phi29 to make DNA nanoball (DNB) which have more than 300 copies of one molecular. The DNBs were load into the patterned nanoarray, and pair end 100/150 bases reads were generated in the way of combinatorial Probe-Anchor Synthesis (cPAS). After the sequencing is completed, raw data were processed using the short oligonucleotide alignment program SOAP to obtain the effective data (clean data), and further aligned with the host sequence using Bowtie2 to remove the reads derived from the host.

### Untargeted metabolomics

The non-targeted metabolomic analysis was performed at the BGI on a platform consisting of an independent ultra-high-performance liquid chromatography–tandem mass spectrometry (UPLC–MS/MS) instrument. Briefly, 6 replicates of samples for each group (100 mg intestinal digesta per replicate) were collected for metabolite extraction. The supernatants were then analyzed using Waters UPLC I-Class Plus (Waters, USA) tandom Q Exactive high resolution mass spectrometer (ThermoFisher Scientific, Waltham, MA, USA) for separation and detection of metabolites. After importing the off-line data of mass spectrometry into Compound Discoverer 3.3 (ThermoFisher Scientific) software and analyzing the mass spectrometry data in combination with bmdb (BGI metabolome database), mzcloud database and chemspider online database, a data matrix containing information such as metabolite peak area and identification results were obtained. The result file from Compound Discoverer was then input to MetaX for data preprocessing and further analysis to screen differential metabolites and enrich metabolic pathways.

### *Bacteroides _caecimuris* treatment

Live *Bacteroides_caecimuris* (NMDC20057801) was obtained from GemPharmatech, China (GPTB0082). The young mice were gavaged with 200 μl *Bacteroides_caecimuris* resuspended in sterile PBS at a dose of 1 × 10^8^ CFU/ml for 14 days. Young mice were gavaged with the equivalent volume of PBS as the control.

### Glutamic acid supplementation

Aged mice were gavaged with 300 μl glutamic acid (Sigma-Aldrich; 100 mg/kg body weight per day, dissolved in saline) or the equivalent volume of saline for 10 consecutive days, followed by hormone injection and oocyte collection. Young mice were gavaged with saline as the control.

### Mouse oocyte collection

Female mice were injected with 10 IU pregnant mare serum gonadotropin (PMSG) followed by 10 IU human chorionic gonadotropin (hCG) 48 h later. Oocytes were harvested from oviductal ampullae after 13–14 h of hCG injection, and briefly exposed to 1 mg/ml hyaluronidase to remove cumulus cells.

### Mouse embryo collection and in vitro culture

To collect zygotes, female mice after hormone priming were mated with males overnight, and euthanized by cervical dislocation at 14 h post-hCG if having the vaginal plug. The zygotes were retrieved from oviductal ampullae in M2 medium containing 1 mg/ml hyaluronidase to remove cumulus cells. All zygotes were then cultured in KSOM medium at 37 °C in an atmosphere of 5% CO_2_ to the specific developmental stage (2-cell embryo: 24 h; blastocyst: 96 h) for further analysis. Presence of 2-cell embryos was considered as the successful fertilization.

### Fertility tests

After FMT or glutamic acid supplementation, female mice were naturally mated with males at ratio of 2:1 for five months, and the number of pups was recorded for each female mouse.

### Fluorescence staining and quantification

Antibody staining, MitoTracker staining, JC-1 staining, DCFH-DA staining, and fluorescence intensity measurement were carried out according to the protocols as we previously described (Miao et al, 2020). The used primary antibodies and dyes were listed as below: α-tubulin-FITC antibody (Sigma-Aldrich, St. Louis, MO, USA; F2168; 1:200), mBBr (ATT bioqueset, Pleasanton, CA, USA; 633; 20 μM), MitoTracker Red CMXRos (ThermoFisher Scientific; M7512; 1:2000), MitoProbe JC-1 Assay Kit (ThermoFisher Scientific; M34152; 1:200), and Reactive Oxygen Species Assay Kit (Beyotime, Shanghai, China; S0033S; 1:800).

### Chromosome spreading

Zona pellucida (ZP) were removed from oocytes by exposure to Tyrode’s buffer (pH 2.5) at 37 °C for 30 s. ZP-free oocytes were then fixed in a solution containing 1% paraformaldehyde and 0.15% Triton X-100 on a glass slide and air dried, followed by staining with Hoechst 33342 for 10 min and imaged by confocal microscope.

### Immunoblotting

As previously described (Zhang et al, 2025), the lysates for mouse ovaries or *Bacteroides_caecimuris* were added to 4 × LDS sample buffer (ThermoFisher Scientific) containing protease inhibitor, and then separated on 10% Bis-Tris precast gels and transferred onto PVDF membranes. The blots were blocked in TBST containing 5% low fat dry milk for 1 h at room temperature and then incubated with GAD1 (Proteintech, Rosemont, IL, USA; 10408-1-AP; 1:5000) or β-Tubulin (TransGen Biotech, Beijing, China; HC101-01; 1:2000) antibodies overnight at 4 °C. After three times of wash in TBST, the blots were incubated with HRP (horse radish peroxidase) conjugated secondary antibodies for 1 h at room temperature. Chemiluminescence signals were detected with Chemistar High-sig ECL Western Blotting Substrate (Tanon, Shanghai, China; 180-501) and protein bands were acquired by Touch Imager Chemiluminescence Imaging System (e-BLOT, Shanghai, China). Band intensities were quantified using ImageJ software and normalized to loading controls.

### DNA extraction and quantitative PCR (qPCR)

Genomic DNA was extracted from mouse intestinal digesta samples using a QIAamp PowerFecal Pro DNA Kit (QIAGEN, Hilden, Germany) according to the manufacturer’s instructions. QPCR was performed using species-specific primers targeting *Bacteroides_caecimuris*. Reactions were conducted using ChamQ Universal SYBR qPCR Master Mix (Vazyme Biotech, Nanjing, China) on a CFX96 Touch Real-Time PCR Detection System (Bio-Rad, Hercules, CA, USA). The relative abundance of *Bacteroides_caecimuris* was normalized to total bacteria quantified using universal bacterial primers and calculated using the 2^−ΔΔCt^ method. The used primers were listed in Table EV1.

### RNA isolation and qRT-PCR

Total mRNA was isolated from 30 oocytes using Dynabeads™ mRNA DIRECT™ Micro kit (ThermoFisher Scientific) according to the manufacturer’s protocol. Following genomic DNA removal, first-strand cDNA synthesis was performed using HiScript II RT SuperMix for qPCR (Vazyme Biotech). Quantitative PCR analysis was conducted on a CFX96 Touch Real-Time PCR Detection System (Bio-Rad) with ChamQ Universal SYBR qPCR Master Mix (Vazyme Biotech). Target gene expression levels were normalized to endogenous reference genes and quantified using the comparative CT method. The used primers for each gene were listed in Table EV1.

### Measurement of glutamic acid content

Amino acids were extracted from 0.03 g of fresh mouse ovaries. Glutamic acid contents were measured using Glutamate Content Assay Kit (Solarbio, Beijing, China; BC5215). The glutamic acid content was determined by comparing the absorbance value with the calibration plot for standard solutions. The absorbance value was measured at 450 nm.

### 16S rRNA gene sequencing

The genomic DNA of the mouse intestinal digesta was extracted using TGuide S96 Magnetic Soil/Stool DNA Kit (Tiangen Biotech, Beijing, China) according to manufacturer’s instructions. The V1-V9 hypervariable regions of the 16S rRNA gene were amplified using primers (27F: AGRGTTTGATYNTGGCTCAG; 1492R: TASGGHTACCTTGTTASGACTT). The amplicons were quantified, after which the normalized equimolar concentrations of amplicons were pooled and sequenced on the PacBio Sequel II platform (Beijing Biomarker Tech, Beijing, China). The qualified sequences with more than 97% similarity thresholds were allocated to one operational taxonomic unit (OTU) using VSEARCH (version 2.4.3).

### Transcriptomic sequencing

Transcriptomic analysis of ovulated oocytes was carried out using a protocol for SMART-seq2 at the BGI. In brief, 4 sets of samples were collected for each group (5 oocytes per sample) in lysis buffer. The procedures for library preparation were conducted based on our previous study (Miao et al, 2020).

### Targeted metabolomics

The targeted metabolomic analysis for 36 neurotransmitter metabolites was performed by high performance liquid chromatography tandem mass spectrometer (LC-MS/MS) using the multi-reaction monitoring (MRM) mode at the BGI. Briefly, 6 replicates of samples for each group (50 mg ovarian tissues per replicate) were collected for metabolite detection with Waters Iclass-AB Sciex 6500 liquid-mass tandem mass spectrometry system, using the waters BEH C18 column (model: 1.7 μm*2.1*100 mm). Mobile phase A: water + 0.1% formic acid, mobile phase B: methanol + 0.1% formic acid; flow rate: 0.35 ml/min; gradient settings: 0–2 min, 2% B, 2.5–15 min, 20–80% B. In Analyst software (SCIEX, USA), the default parameters were used for automatic identification and integration of each MRM transition (ion pair), and manual inspection was assisted. The content of neurotransmitters (ng/ml) = (C*V)/M; where C was the concentration value obtained by substituting the integrated peak area of the target index in the sample into the standard curve (ng/ml), and V was the volume of the extracted solution (μl), M was the actual sample weighing mass (mg).

### Porcine oocyte collection and in vitro maturation

Abattoir-derived porcine ovaries were transported to the laboratory within 2 h in a physiological saline containing penicillin G/streptomycin sulfate. The procedures for porcine oocyte collection and in vitro maturation were performed as we described previously (Miao et al, 2022).

### Statistical analysis

No statistical methods were used to pre-determine sample sizes but our sample sizes are similar to those reported in previous publications (Miao et al, 2020; Zhang et al, 2023). Data collection and analysis were not performed blinded to the conditions of the experiments, and no data were excluded from the analyses. All statistical data from at least three independent experiments were presented as mean ± SEM or SD unless otherwise stated, and the number of samples used in each group was labeled in parentheses as (*n*). Data were analyzed by two-tailed unpaired t-test, which is provided by GraphPad Prism 8 statistical software. *P* < 0.05 was considered as statistical significance.

## Supplementary information

Table EV1

Peer Review File

Dataset EV1

Dataset EV2

Dataset EV3

Dataset EV4

Dataset EV5

Dataset EV6

Dataset EV7

Dataset EV8

Source data Fig. 2

Source data Fig. 3

Source data Fig. 4

Source data Fig. 6

Source data Fig. 7

Source data Fig. 8

Expanded View Figures

## Data Availability

The datasets produced in this study are available in the following databases: Metagenome data and RNA-Seq data: Gene Expression Omnibus GSE325162; Metabolomics data: MetaboLights MTBLS14062. The source data of this paper are collected in the following database record: biostudies:S-SCDT-10_1038-S44321-026-00443-3.
