# Author Correction: Gut microbiota-modulated glutamic acid rejuvenates the quality of oocytes deteriorated by advanced reproductive age

**DOI:** 10.1038/s44321-026-00457-x

**Published:** 2026-05-28

**Authors:** Feixue Wang, Wenjun Zeng, Zihao Zhang, Na Li, Zhaokang Cui, Jie Bai, Jiner Yan, Yu Zhang, Yilong Miao, Ling Gu, Bo Xiong

**Affiliations:** 1 College of Animal Science and Technology, Nanjing Agricultural University, Nanjing, 210095 China; 2 College of Animal Sciences, Zhejiang University, Hangzhou, 310058 China

**Correction to:** *EMBO Molecular Medicine* 2026. 10.1038/s44321-026-00443-3 | Published online 08 May 2026

In the published version of this manuscript, several figure annotations were inadvertently omitted during production. The missing labels have now been restored in the online version of the article.

**The following figures are corrected: Figures 2, 3, 6, 7, EV3, EV5, EV6, EV9, EV10, and EV11**.

The following annotations were affected:

- “YY-FMT” in Figure 2C,E,F,G,H,J,K,M; Figure 3G; and Figure EV6J–O.
- “Young” in Figure 6C,D,E; Figure 7C,D,E,F,H,I,K,M; Figure EV5B, C,D,E,G,H,J,L; and Figure EV9B,D.
- “YY-FMT-15” in Figure EV3B–I.
- “YY-FMT-30” in Figure EV3J–Q.
- “Aged+Glu” in Figure EV10B.
- “Control” and “H2O2” in Figure EV11B,D,E,G,I.
- “PB1 extrusion” in Figure EV5E.

These corrections do not affect the results or conclusions of the study.

