# Supplementary material for: Gut microbiota-modulated glutamic acid rejuvenates the quality of oocytes deteriorated by advanced reproductive age
Source: EMBO Mol Med. 2026 May 8;18(6):2404–35. doi: 10.1038/s44321-026-00443-3 (PMC13270145; doi:10.1038/s44321-026-00443-3)
Supplement: Supplementary file 1 — Table EV1 [file 44321_2026_443_MOESM1_ESM.doc]

**Table EV1. Primer sequences for quantitative PCR.**

| **Name** | **Primer sequence** | **NCBI reference sequence** |
| --- | --- | --- |
| *Total bacteria*  *Bacteroides_caecimuris*  *Nfe2l2*  *Gpx4*  *Sod1*  *Prdx3*  *Gapdh* | F: CGGTGAATACGTTCCCGG  R: TACGGCTACCTTGTTACGACTT  F: GCATGCGCTATGGTATGTGG  R: CAGGAAGCTCCACTTCCGTC  F: TCTTGGAGTAAGTCGAGAAGTGT  R: GTTGAAACTGAGCGAAAAAGGC  F: AGTACAGGGGTTTCGTGTGC  R: CATGCAGATCGACTAGCTGAG  F: GGAACCATCCACTTCGAGCA  R: CCCATGCTGGCCTTCAGTTA  F: GGTTGCTCGTCATGCAAGTG  R: CCACAGTATGTCTGTCAAACAGG  F: AGGTCGGTGTGAACGGATTTG  R: TGTAGACCATGTAGTTGAGGTCA | NM_010902.5  NM_001037741.4  NM_011434.2  NM_007452.2  NM_001411840.1 |
