# Supplementary figures and images for: Gut microbiota-modulated glutamic acid rejuvenates the quality of oocytes deteriorated by advanced reproductive age
Source: EMBO Mol Med. 2026 May 8;18(6):2404–35. doi: 10.1038/s44321-026-00443-3 (PMC13270145; doi:10.1038/s44321-026-00443-3)

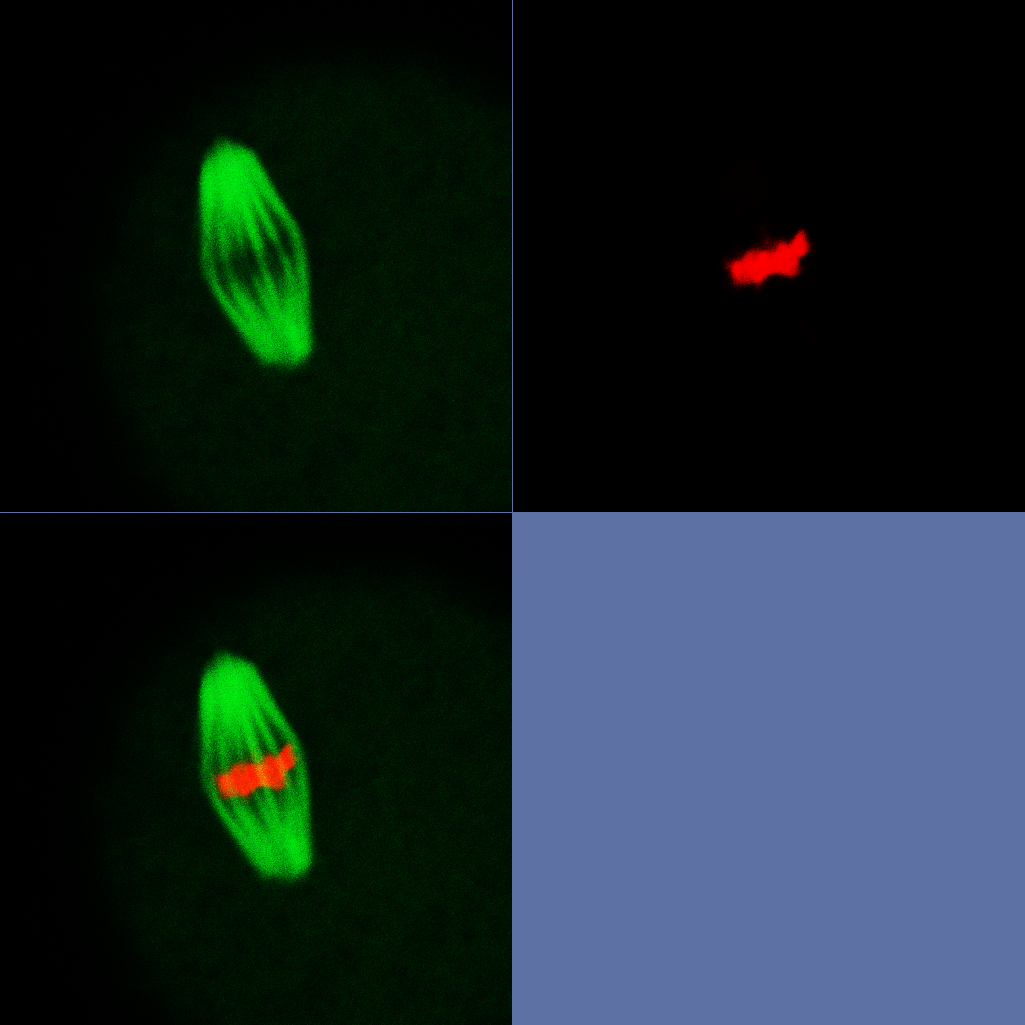

Supplement: Supplementary file 11 — Source data Fig. 2 [file 44321_2026_443_MOESM11_ESM.zip › Figure 2/2I/╬▒-tubulin YY-FMT.tif]

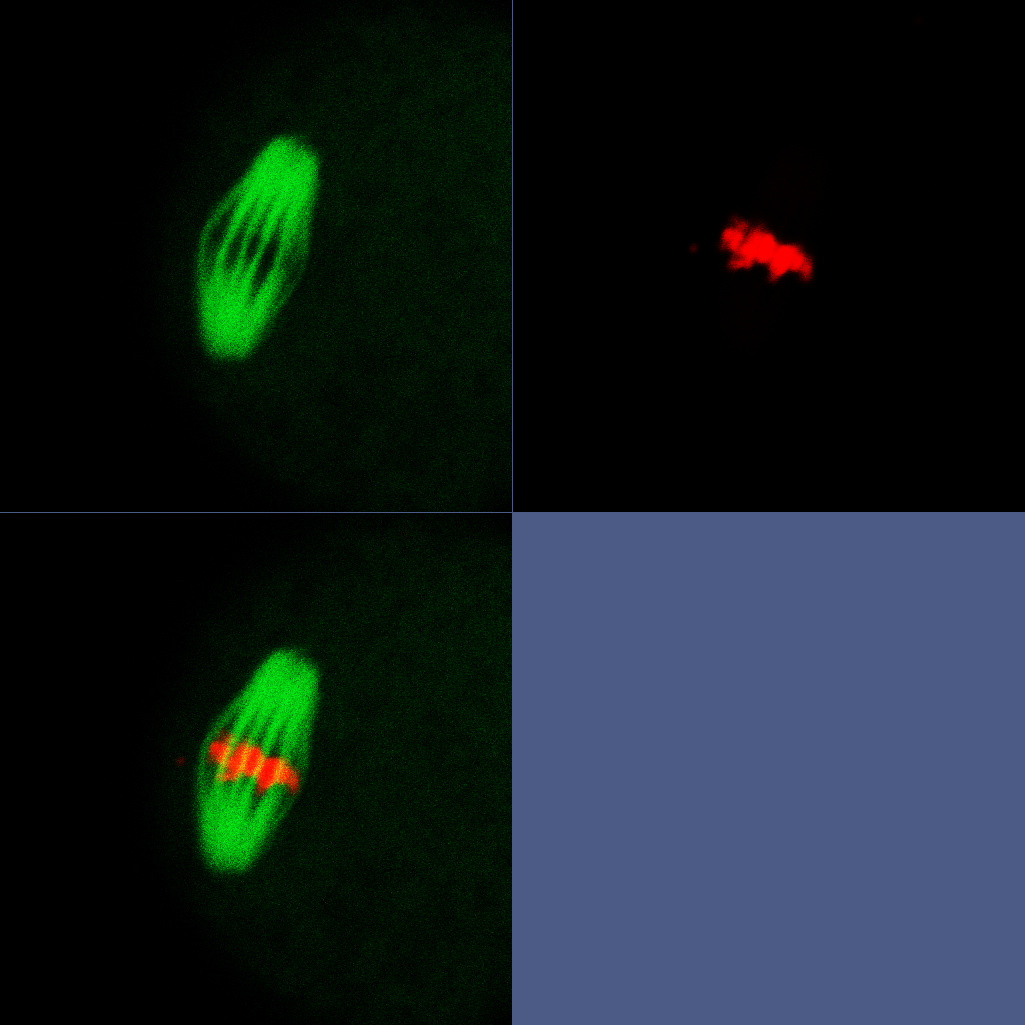

Supplement: Supplementary file 11 — Source data Fig. 2 [file 44321_2026_443_MOESM11_ESM.zip › Figure 2/2I/╬▒-tubulin YA-FMT.tif]

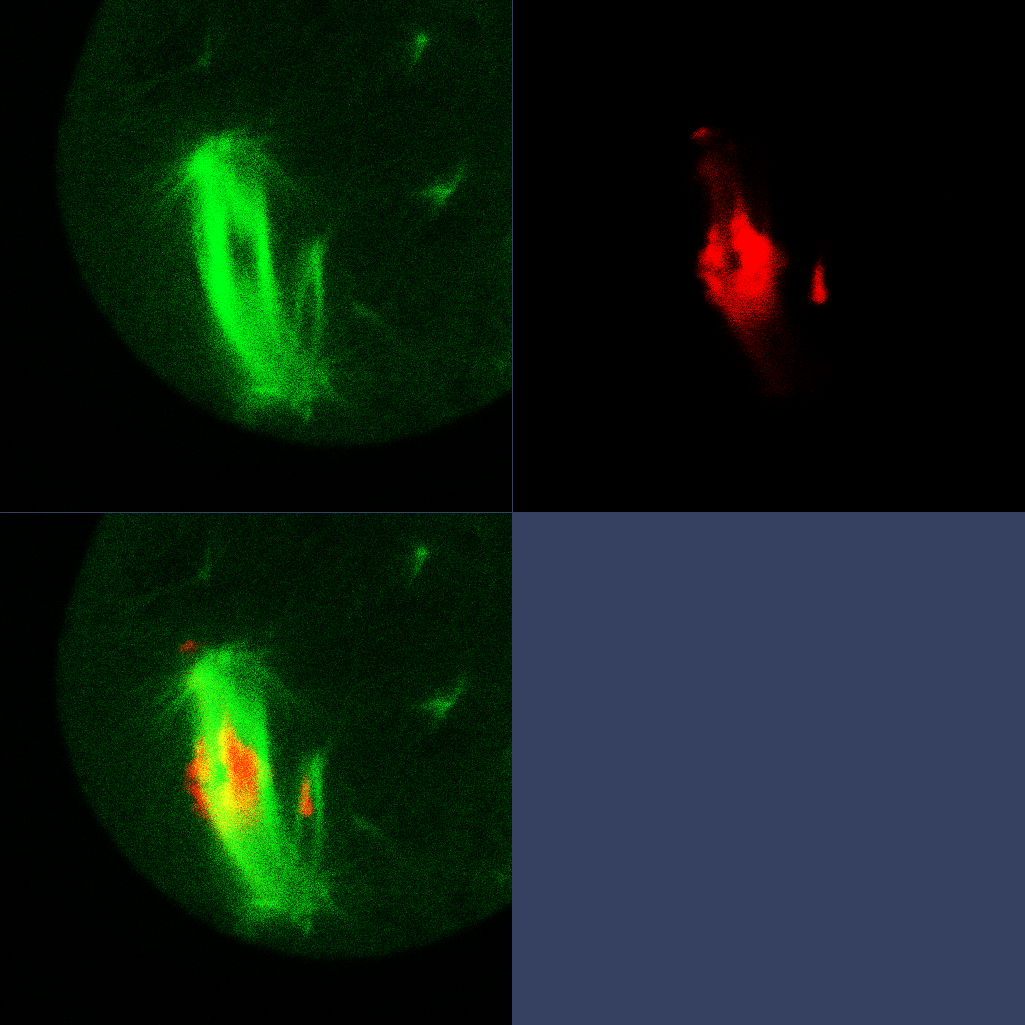

Supplement: Supplementary file 11 — Source data Fig. 2 [file 44321_2026_443_MOESM11_ESM.zip › Figure 2/2I/╬▒-tubulin AA-FMT.tif]

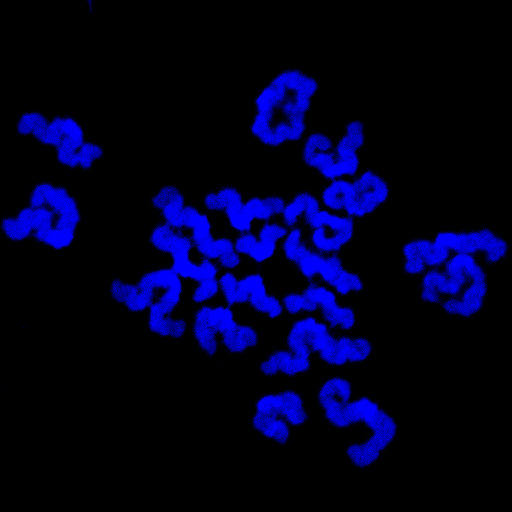

Supplement: Supplementary file 11 — Source data Fig. 2 [file 44321_2026_443_MOESM11_ESM.zip › Figure 2/2L/Chromosome spreading YY-FMT.tif]

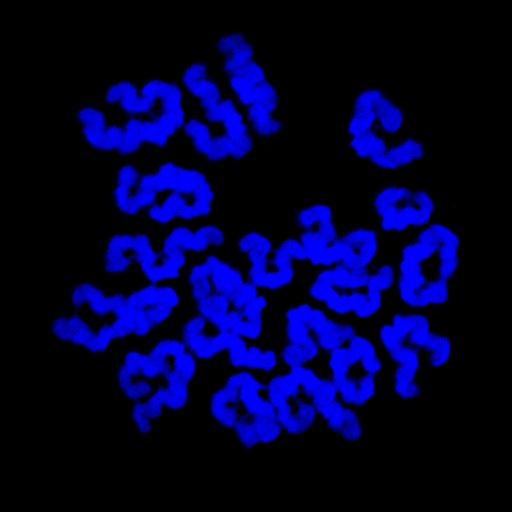

Supplement: Supplementary file 11 — Source data Fig. 2 [file 44321_2026_443_MOESM11_ESM.zip › Figure 2/2L/Chromosome spreading YA-FMT.tif]

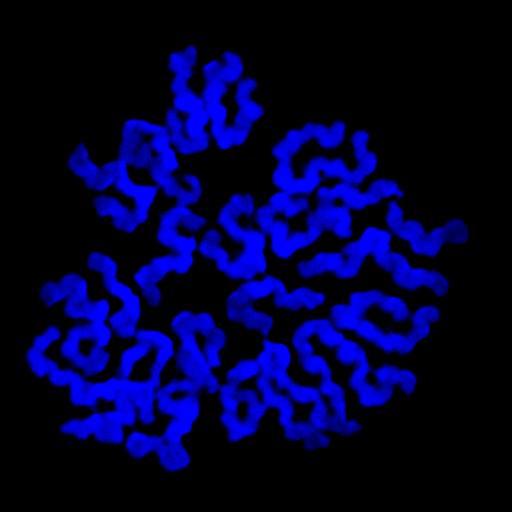

Supplement: Supplementary file 11 — Source data Fig. 2 [file 44321_2026_443_MOESM11_ESM.zip › Figure 2/2L/Chromosome spreading AA-FMT.tif]

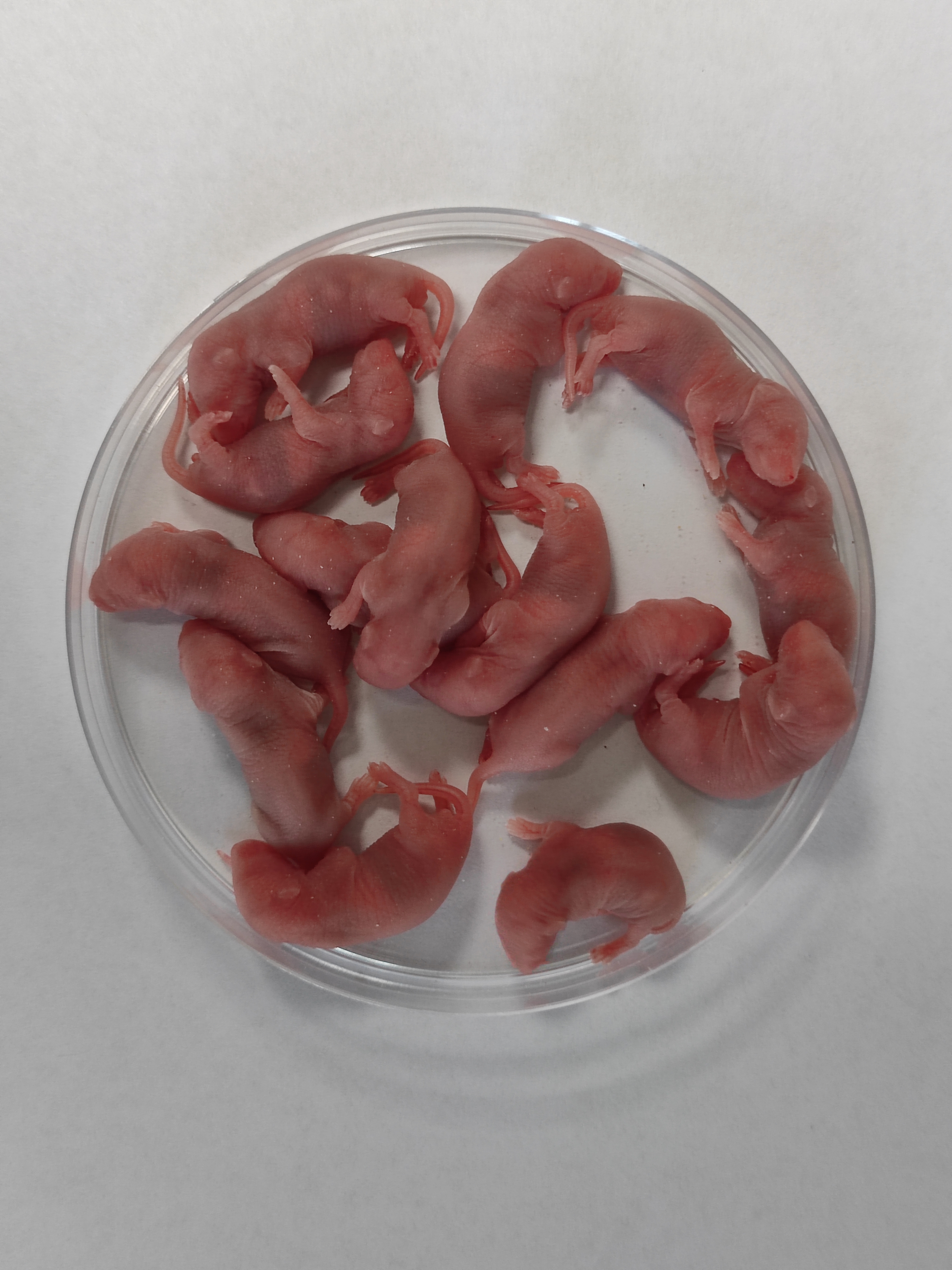

Supplement: Supplementary file 11 — Source data Fig. 2 [file 44321_2026_443_MOESM11_ESM.zip › Figure 2/2B/Fertility YY-FMT.jpg]

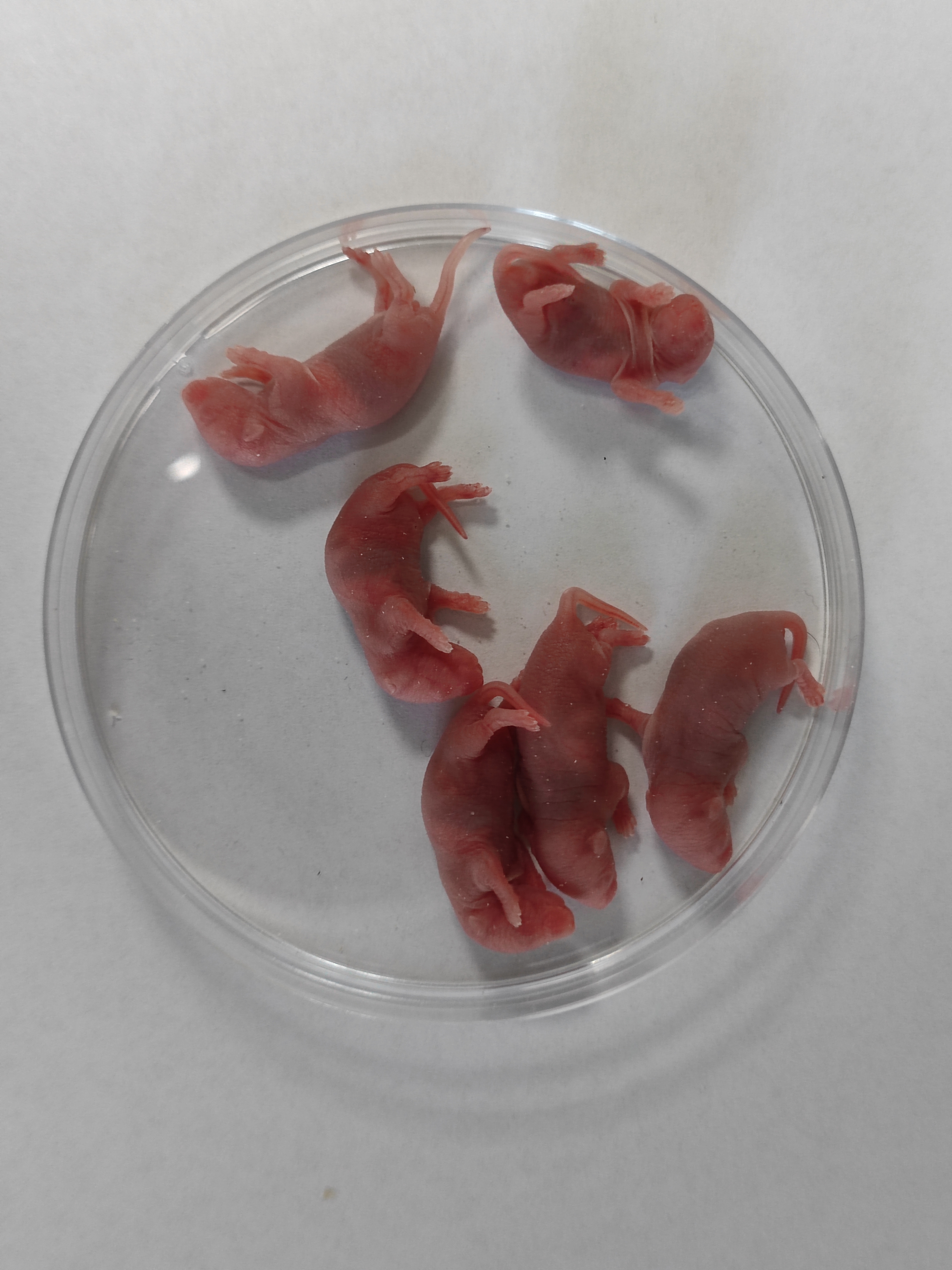

Supplement: Supplementary file 11 — Source data Fig. 2 [file 44321_2026_443_MOESM11_ESM.zip › Figure 2/2B/Fertility YA-FMT.jpg]

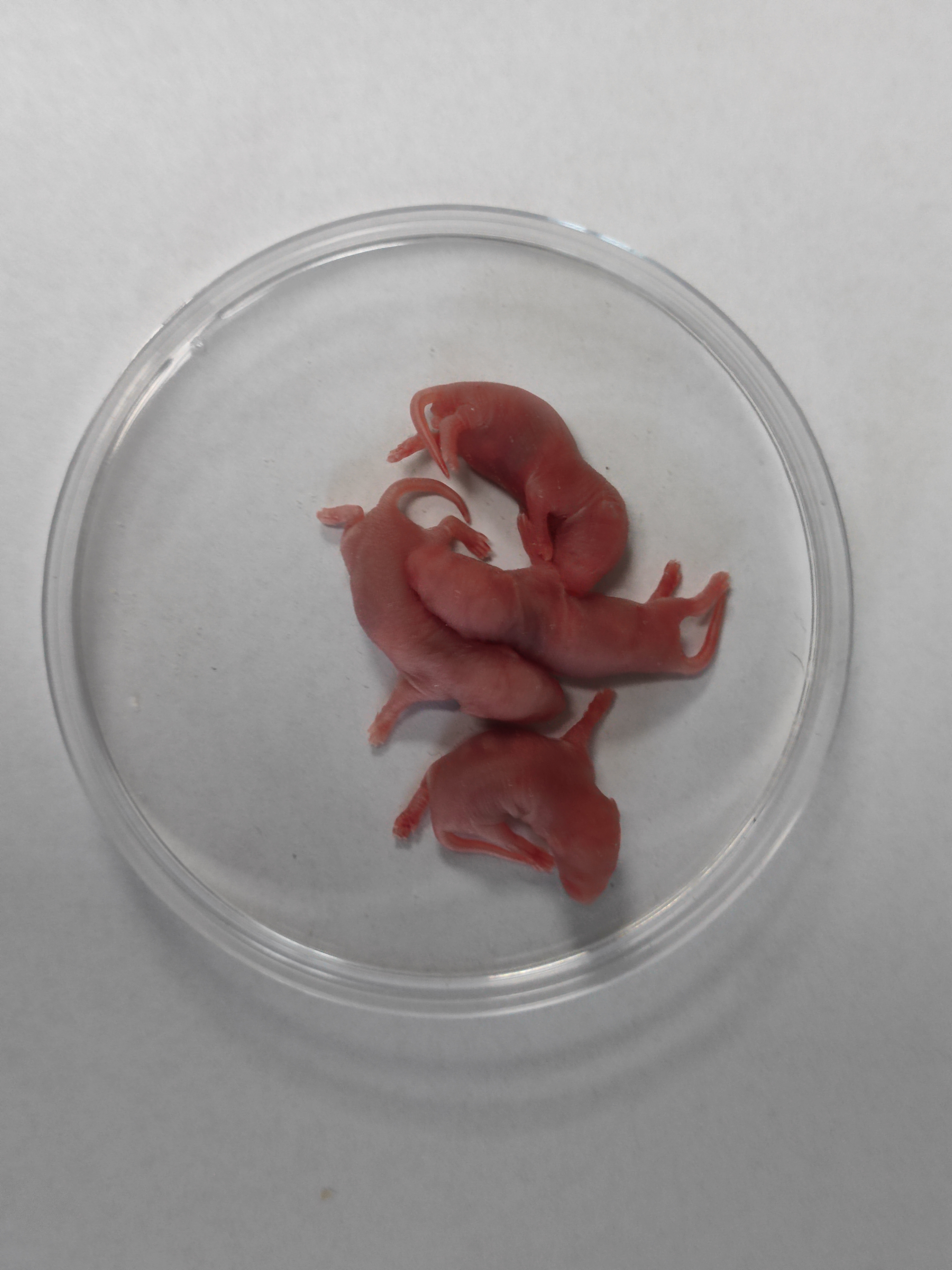

Supplement: Supplementary file 11 — Source data Fig. 2 [file 44321_2026_443_MOESM11_ESM.zip › Figure 2/2B/Fertility AA-FMT.jpg]

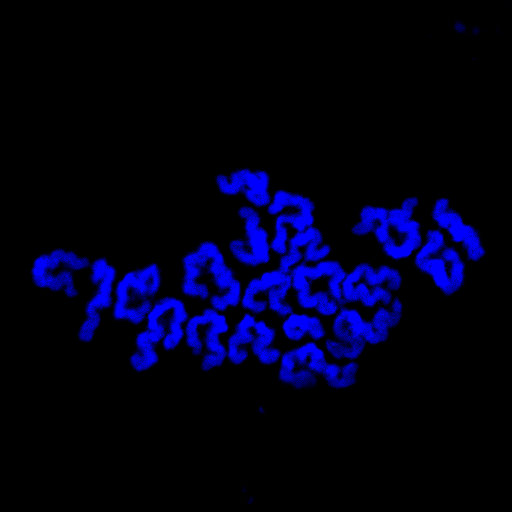

Supplement: Supplementary file 15 — Source data Fig. 7 [file 44321_2026_443_MOESM15_ESM.zip › Figure 7/7J/Chromosome spreading Young.tif]

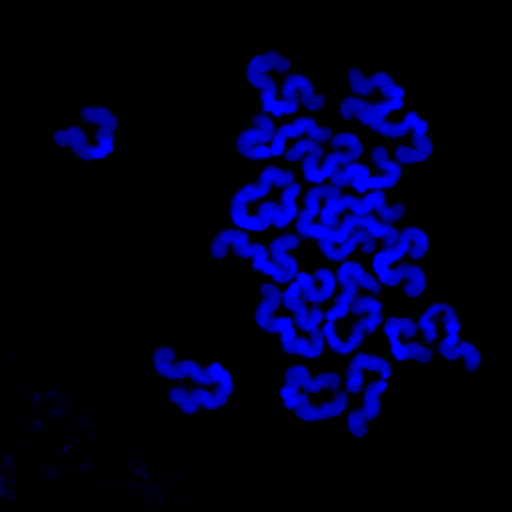

Supplement: Supplementary file 15 — Source data Fig. 7 [file 44321_2026_443_MOESM15_ESM.zip › Figure 7/7J/Chromosome spreading Aged+Glu.tif]

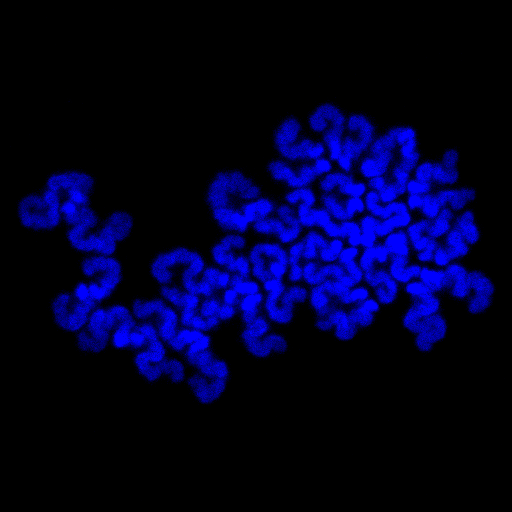

Supplement: Supplementary file 15 — Source data Fig. 7 [file 44321_2026_443_MOESM15_ESM.zip › Figure 7/7J/Chromosome spreading Aged.tif]

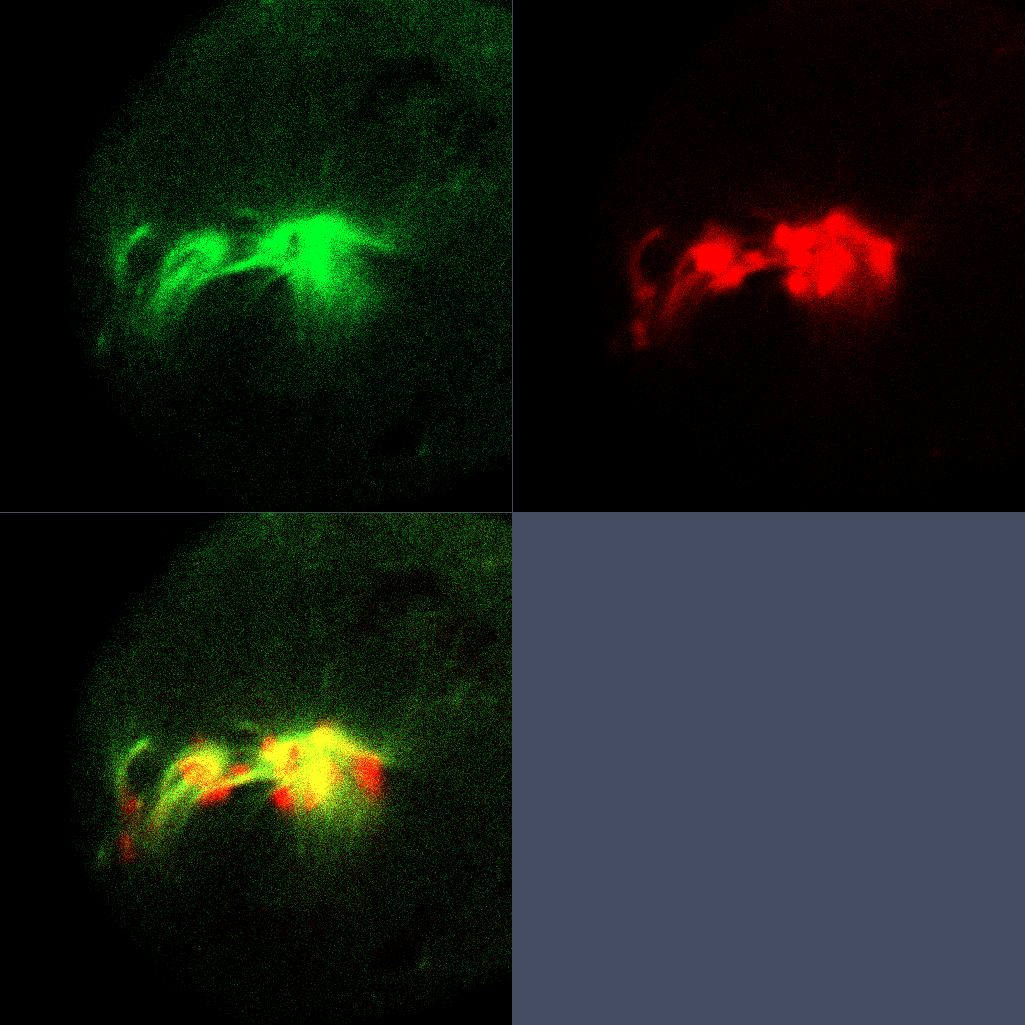

Supplement: Supplementary file 15 — Source data Fig. 7 [file 44321_2026_443_MOESM15_ESM.zip › Figure 7/7G/Tubulin Aged.tif]

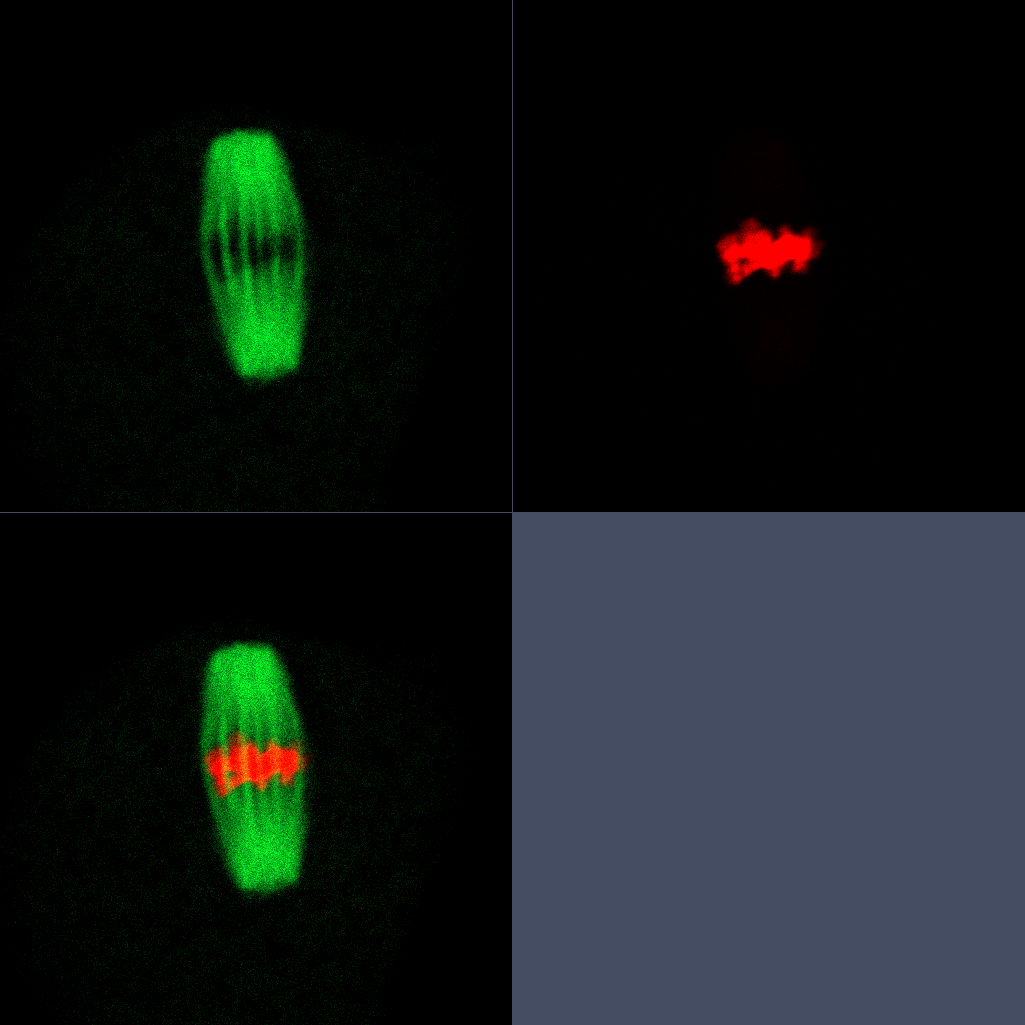

Supplement: Supplementary file 15 — Source data Fig. 7 [file 44321_2026_443_MOESM15_ESM.zip › Figure 7/7G/Tubulin Young.tif]

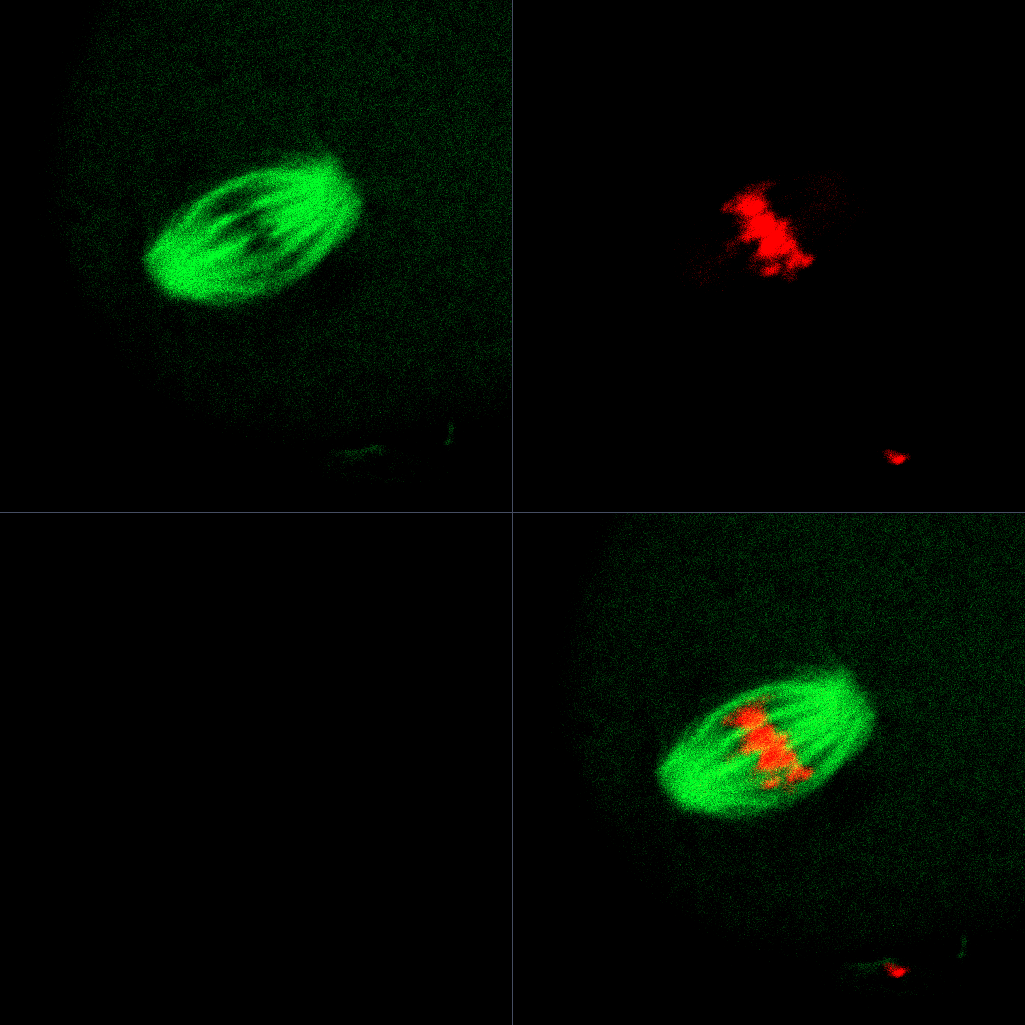

Supplement: Supplementary file 15 — Source data Fig. 7 [file 44321_2026_443_MOESM15_ESM.zip › Figure 7/7G/Tubulin Aged+GLU.tif]

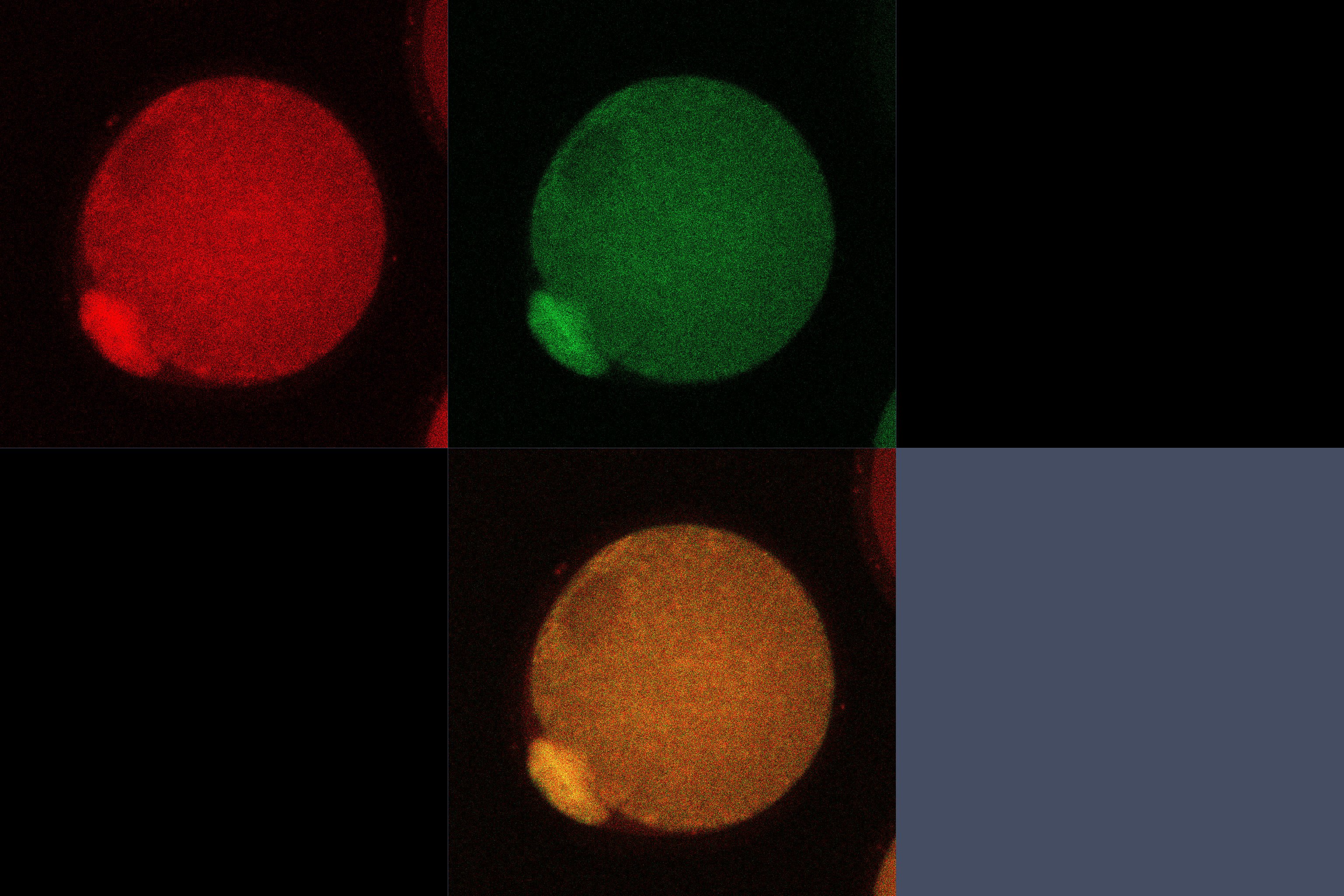

Supplement: Supplementary file 16 — Source data Fig. 8 [file 44321_2026_443_MOESM16_ESM.zip › Figure 8/8I/Young.tif]

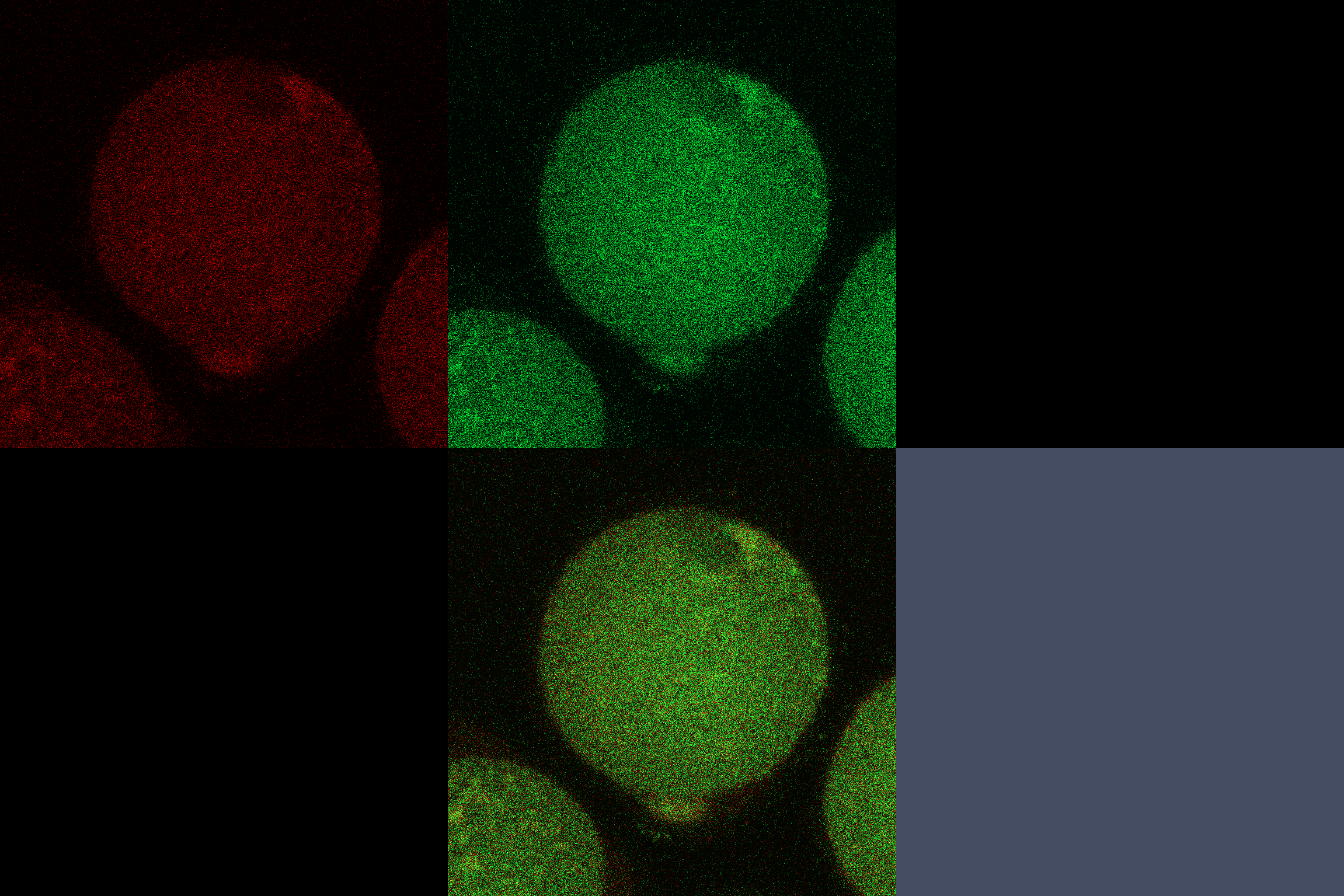

Supplement: Supplementary file 16 — Source data Fig. 8 [file 44321_2026_443_MOESM16_ESM.zip › Figure 8/8I/Aged.tif]

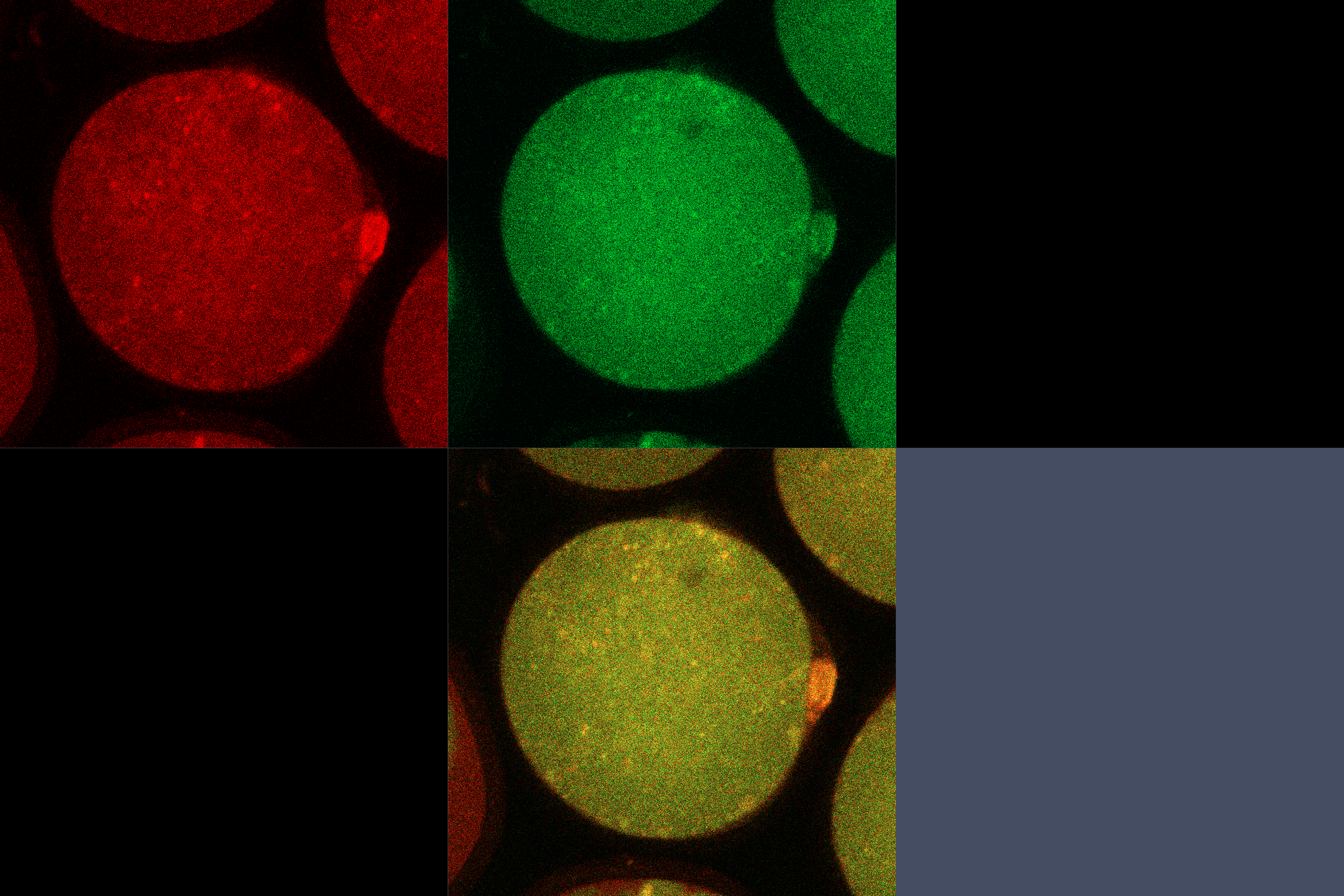

Supplement: Supplementary file 16 — Source data Fig. 8 [file 44321_2026_443_MOESM16_ESM.zip › Figure 8/8I/Aged+Glu.tif]

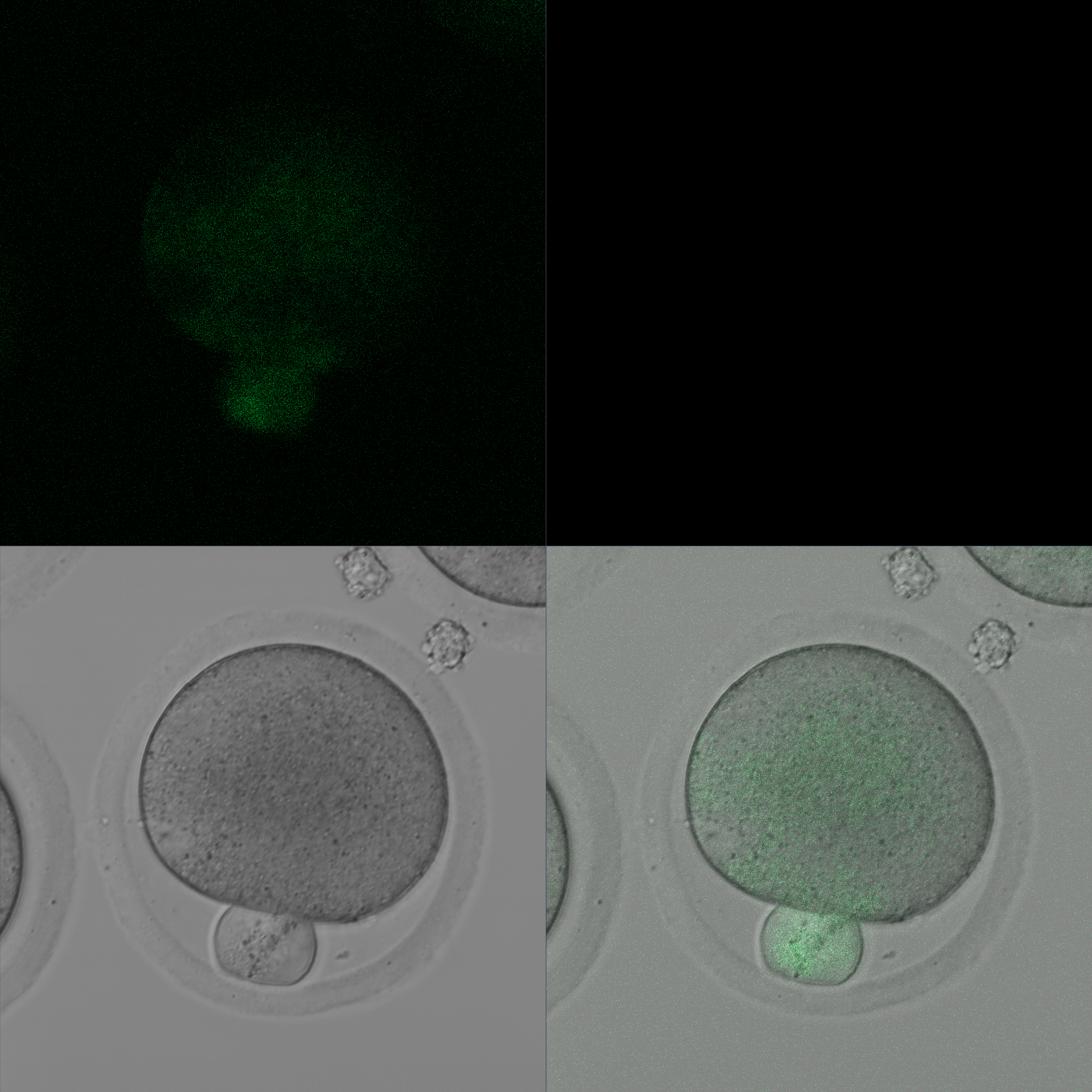

Supplement: Supplementary file 16 — Source data Fig. 8 [file 44321_2026_443_MOESM16_ESM.zip › Figure 8/8K/Young.tif]

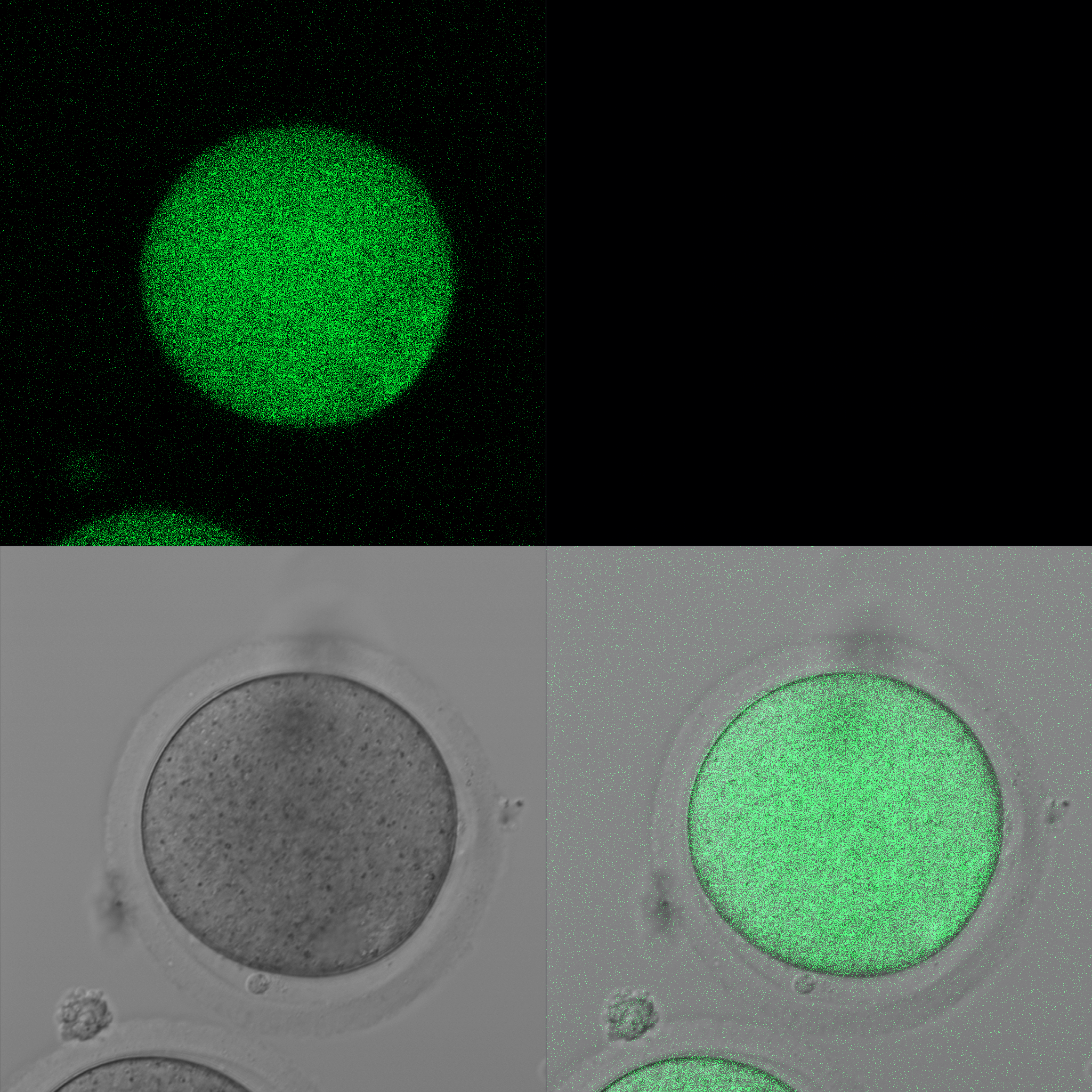

Supplement: Supplementary file 16 — Source data Fig. 8 [file 44321_2026_443_MOESM16_ESM.zip › Figure 8/8K/Aged.tif]

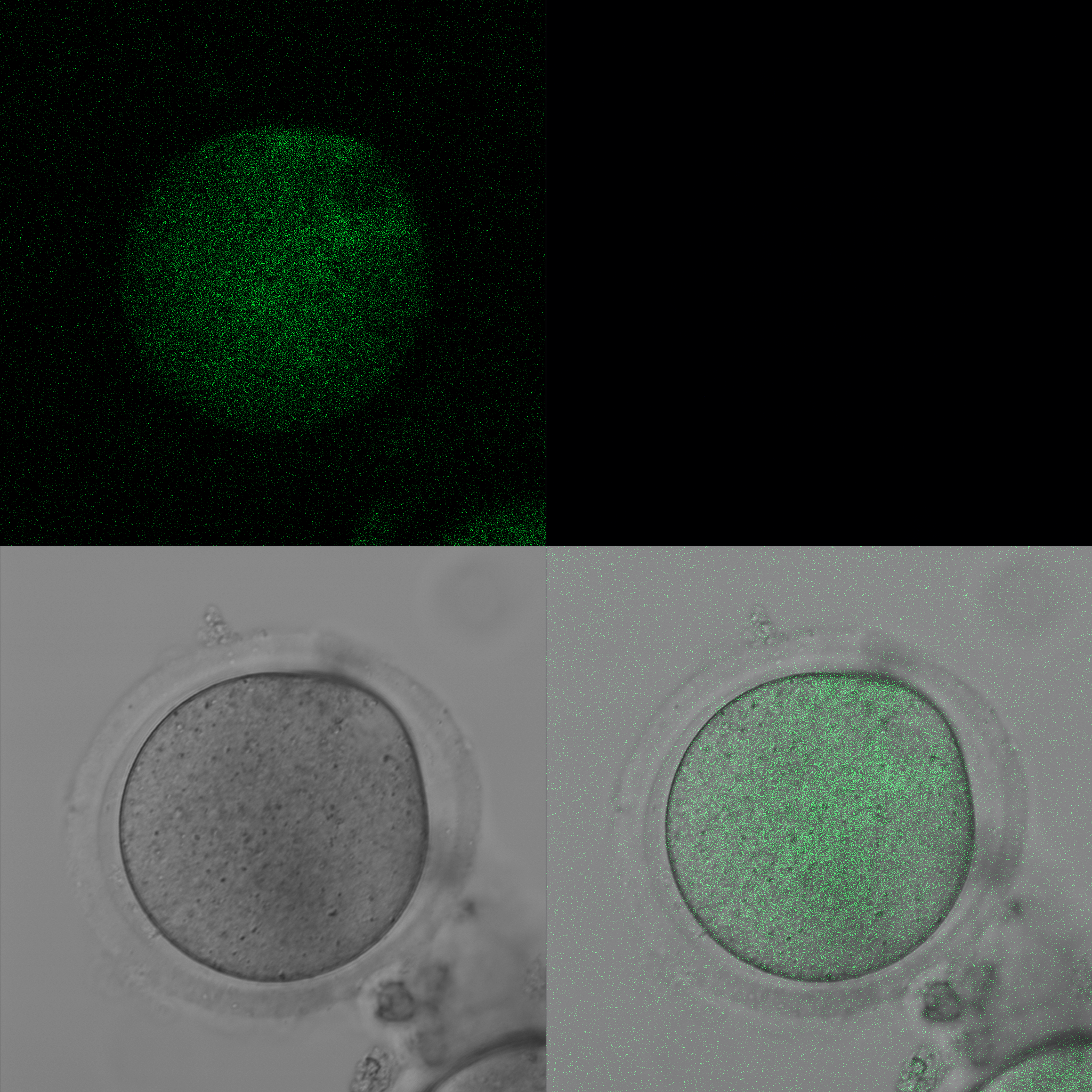

Supplement: Supplementary file 16 — Source data Fig. 8 [file 44321_2026_443_MOESM16_ESM.zip › Figure 8/8K/Aged+GLU.tif]
