# Supplementary material for: Gut microbiota-modulated glutamic acid rejuvenates the quality of oocytes deteriorated by advanced reproductive age
Source: EMBO Mol Med. 2026 May 8;18(6):2404–35. doi: 10.1038/s44321-026-00443-3 (PMC13270145; doi:10.1038/s44321-026-00443-3)
Supplement: Supplementary file 17 — Expanded View Figures [file 44321_2026_443_MOESM17_ESM.pdf]

Expanded View Figures

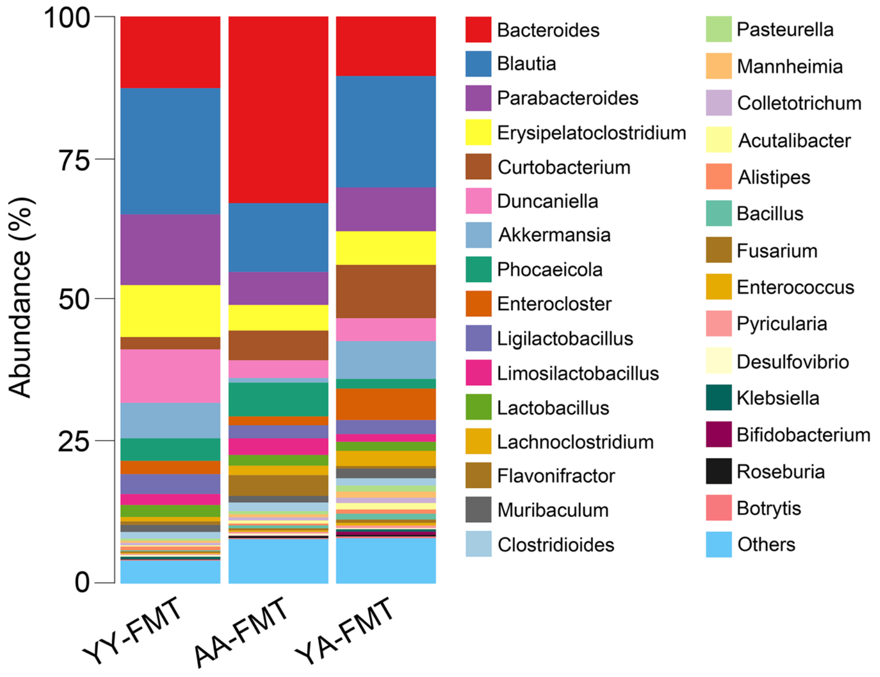

Figure EV1. Effects of FMT on the bacterial abundance in mice.

The degree of bacterial taxonomic similarity of gut microbiota was analyzed in YY-FMT, AA-FMT, and YA-FMT groups at genus level.

 © The Author(s)

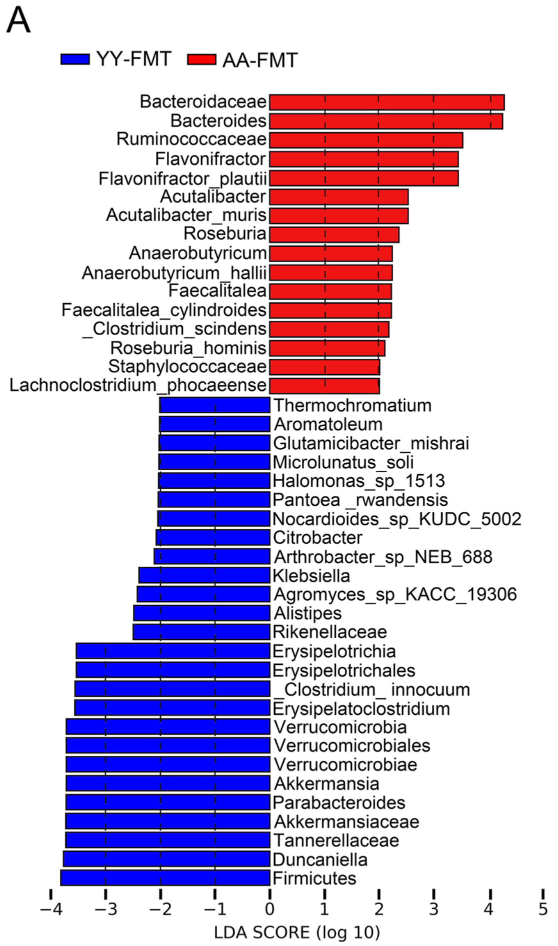

Figure EV2. Effects of FMT on the bacterial taxa in mice.

(**A**) LEfSe showed dominant bacterial taxa in AA-FMT group compared to YY-FMT group. Blue bar represented YY-FMT, and red bar represented AA-FMT. (**B**) LEfSe showed dominant bacterial taxa in YA-FMT group compared to AA-FMT group. Red bar represented AA-FMT, and green bar represented YA-FMT.

© The Author(s)

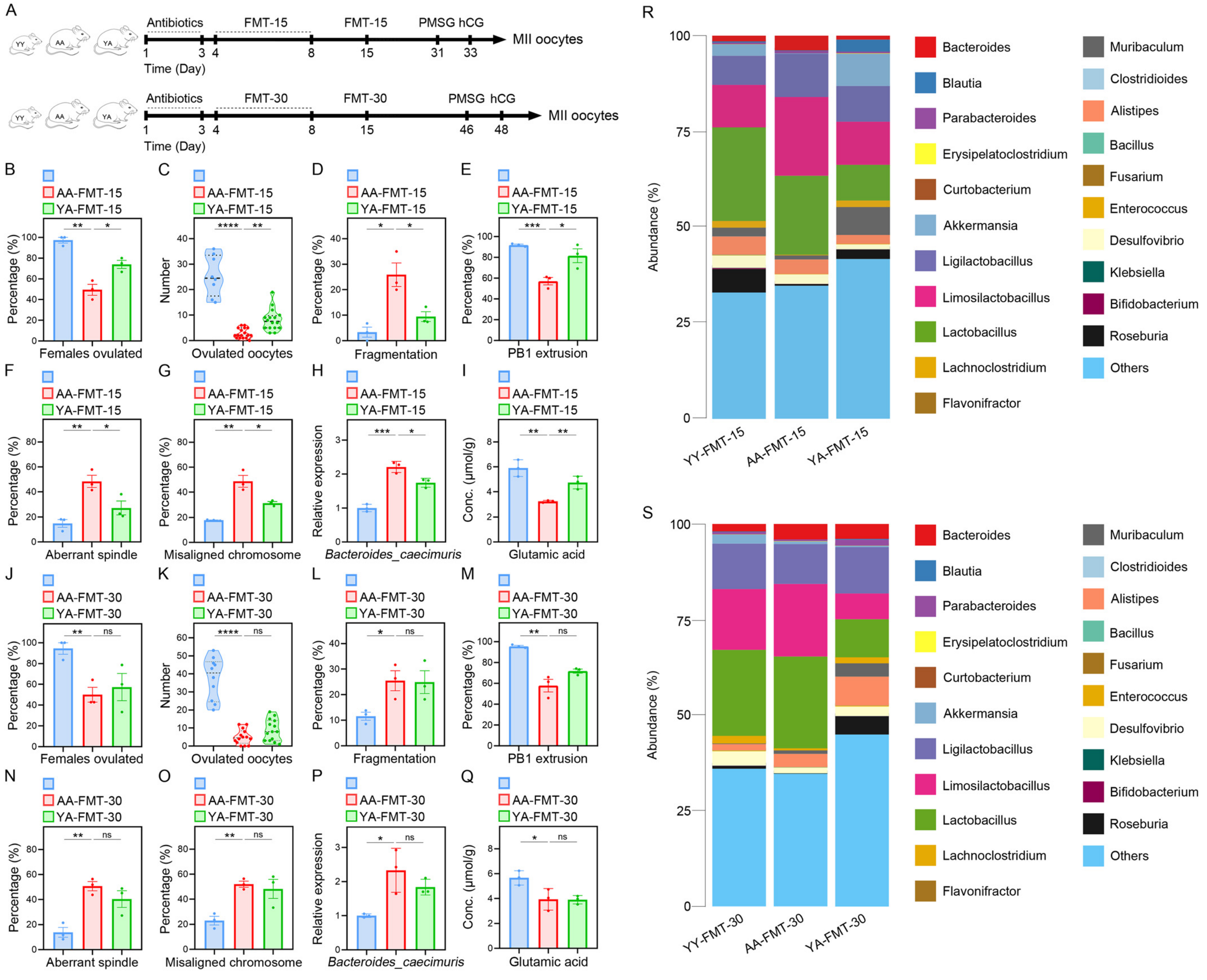

Figure EV3. Effects of young gut microbiota on the quality of aged oocytes on days 15 and 30 after FMT.

(**A**) A timeline scheme for antibiotics treatment, FMT, hormone injection and subsequent analyses. (**B**) The percentage of female mice that were ovulated in YY-FMT-15 ($n = 30$), AA-FMT-15 ($n = 36$), and YA-FMT-15 ($n = 38$) group. **$P = 0.0013$, *$P = 0.0205$. (**C**) The number of ovulated oocytes was counted in YY-FMT-15 ($n = 8$), AA-FMT-15 ($n = 17$), and YA-FMT-15 ($n = 16$) mice. ****$P < 0.0001$, **$P = 0.0032$. (**D**) The proportion of fragmented oocytes was quantified in YY-FMT-15 ($n = 112$), AA-FMT-15 ($n = 67$), and YA-FMT-15 ($n = 96$) mice. *$P = 0.0109$, *$P = 0.0303$. (**E**) The rate of PB1 extrusion was quantified in YY-FMT-15 ($n = 112$), AA-FMT-15 ($n = 67$), and YA-FMT-15 ($n = 96$) oocytes. **$P = 0.0007$, *$P = 0.0312$. (**F**) The rate of aberrant spindles was quantified in YY-FMT-15 ($n = 73$), AA-FMT-15 ($n = 110$), and YA-FMT-15 ($n = 67$) oocytes at MII stage. **$P = 0.0044$, *$P = 0.0445$. (**G**) The rate of misaligned chromosomes was quantified in YY-FMT-15 ($n = 73$), AA-FMT-15 ($n = 110$), and YA-FMT-15 oocytes ($n = 67$) at M II stage. **$P = 0.0029$, *$P = 0.0241$. (**H**) The abundance of *Bacteroides_caecimuris* was determined by qPCR in YY-FMT-15, AA-FMT-15, and YA-FMT-15 mice. ***$P = 0.0005$, *$P = 0.0186$. (**I**) Glutamic acid levels were quantified in YY-FMT-15 ($n = 21$), AA-FMT-15 ($n = 16$), and YA-FMT-15 ($n = 15$) ovaries. **$P = 0.0073$, **$P = 0.0024$. (**J**) The percentage of female mice that were ovulated in YY-FMT-30 ($n = 32$), AA-FMT-30 ($n = 42$), and YA-FMT-30 ($n = 36$) group. **$P = 0.0080$, $P = 0.6508$. (**K**) The number of ovulated oocytes was counted in YY-FMT-30 ($n = 10$), AA-FMT-30 ($n = 14$), and YA-FMT-30 ($n = 14$) mice. ****$P < 0.0001$, $P = 0.0604$. (**L**) The proportion of fragmented oocytes was quantified in YY-FMT-30 ($n = 492$), AA-FMT-30 ($n = 217$), and YA-FMT-30 ($n = 151$) mice. *$P = 0.0299$, $P = 0.9346$. (**M**) The rate of PB1 extrusion was quantified in YY-FMT-30 ($n = 492$), AA-FMT-30 ($n = 217$), and YA-FMT-30 ($n = 151$) oocytes. **$P = 0.0035$, $P = 0.0931$. (**N**) The rate of aberrant spindles was quantified in YY-FMT-30 ($n = 98$), AA-FMT-30 ($n = 114$), and YA-FMT-30 ($n = 107$) oocytes at M II stage. **$P = 0.0024$, $P = 0.2500$. (**O**) The rate of misaligned chromosomes was quantified in YY-FMT-30 ($n = 98$), AA-FMT-30 ($n = 114$), and YA-FMT-30 oocytes ($n = 107$) at MII stage. **$P = 0.0023$, $P = 0.6616$. (**P**) The abundance of *Bacteroides_caecimuris* was determined by qPCR in YY-FMT-30, AA-FMT-30, and YA-FMT-30 mice. *$P = 0.0235$, $P = 0.2804$. (**Q**) Glutamic acid levels were quantified in YY-FMT-30 ($n = 22$), AA-FMT-30 ($n = 16$), and YA-FMT-30 ($n = 16$) ovaries. **$P = 0.0453$, $P = 0.9562$. (**R**) The degree of bacterial taxonomic similarity of gut microbiota was analyzed in YY-FMT-15, AA-FMT-15, and YA-FMT-15 groups at genus level. (**S**) The degree of bacterial taxonomic similarity of gut microbiota was analyzed in YY-FMT-30, AA-FMT-30, and YA-FMT-30 groups at genus level. Data in (**B–Q**) were presented as mean ± SEM or SD of at least three independent experiments. *$P < 0.05$; **$P < 0.01$; ***$P < 0.001$; ****$P < 0.0001$; ns, no significance. Statistical significance was determined by two-tailed unpaired t-test.

 © The Author(s)

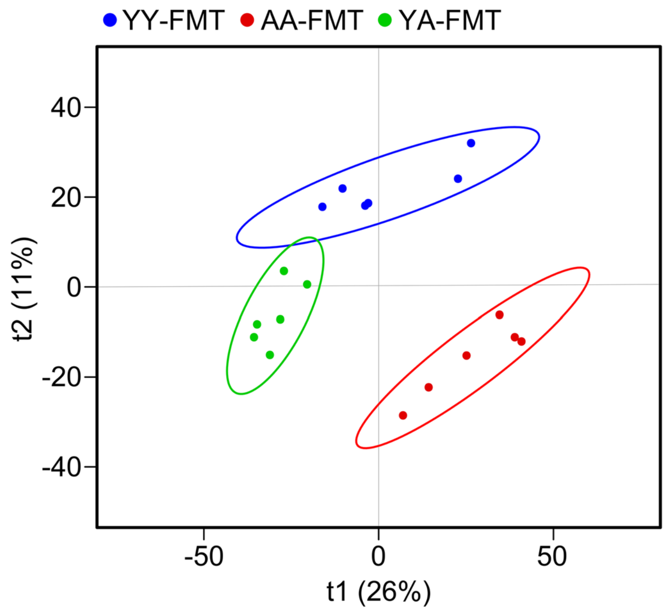

Figure EV4. PLS-DA plot showing the metabolome samples from FMT mice.

Blue circles represented samples from YY-FMT group, red circles represented samples from AA-FMT group, and green circles represented samples from YA-FMT group.

© The Author(s)

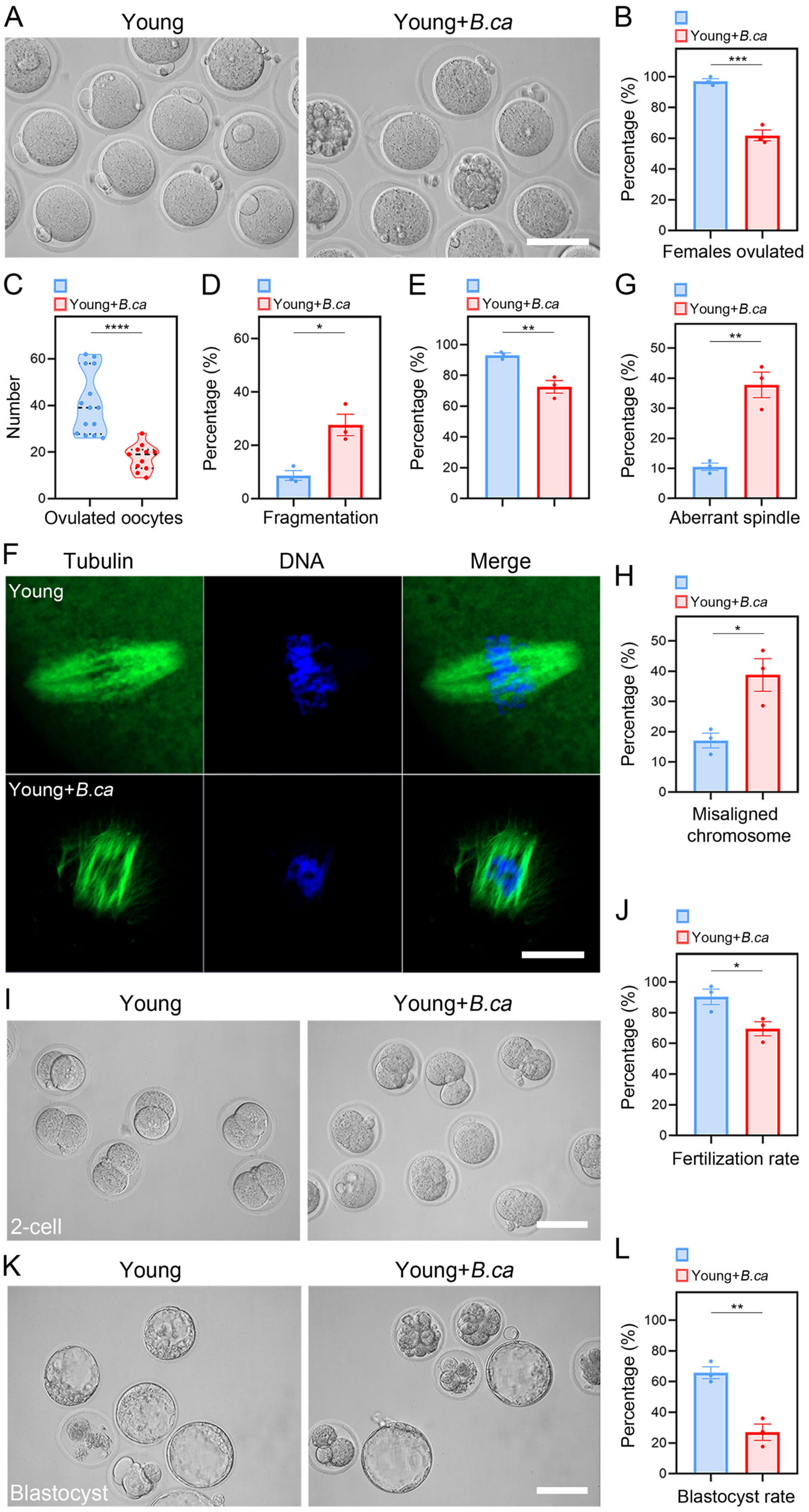
A
Young
Young+B.ca
B
Young+B.ca
***
Percentage (%)
Females ovulated
C
Young+B.ca
****
Number
Ovulated oocytes
D
Young+B.ca
*
Percentage (%)
Fragmentation
E
Young+B.ca
**
Percentage (%)
G
Young+B.ca
**
Percentage (%)
Aberrant spindle
F
Tubulin
DNA
Merge
Young
Young+B.ca
H
Young+B.ca
*
Percentage (%)
Misaligned chromosome
I
Young
Young+B.ca
2-cell
J
Young+B.ca
*
Percentage (%)
Fertilization rate
K
Young
Young+B.ca
Blastocyst
L
Young+B.ca
**
Percentage (%)
Blastocyst rate

 © The Author(s)

Figure EV5. Effects of *Bacteroides_caecimuris* supplementation in young mice on the oocyte quality.

(**A**) Representative images of ovulated oocytes collected from young and young+*B.ca* mice. Scale bar, 80 μm. (**B**) The percentage of female mice that were ovulated in young ($n = 86$) and young+*B.ca* ($n = 118$) group. ***$P = 0.0008$. (**C**) The number of ovulated oocytes was counted in young ($n = 14$) and young+*B.ca* ($n = 11$) mice. ****$P < 0.0001$. (**D**) The proportion of fragmented oocytes was quantified in young ($n = 575$) and young+*B.ca* ($n = 194$) mice. *$P = 0.0126$. (**E**) The rate of PB1 extrusion was quantified in young ($n = 575$) and young+*B.ca* ($n = 194$) oocytes. **$P = 0.0087$. (**F**) Representative images of the spindle morphology and chromosome alignment in young and young+*B.ca* oocytes at M II stage. Scale bar, 20 μm. (**G**) The rate of aberrant spindles was quantified in young ($n = 76$) and young+*B.ca* ($n = 111$) oocytes at M II stage. **$P = 0.0035$. (**H**) The rate of misaligned chromosomes was quantified in young ($n = 76$) and young+*B.ca* ($n = 111$) at M II stage. *$P = 0.0213$. (**I**) Representative images of 2-cell embryos in young and young+*B.ca* groups. Scale bar, 80 μm. (**J**) The fertilization rate was quantified in young ($n = 101$) and young+*B.ca* ($n = 85$) groups. *$P = 0.0373$. (**K**) Representative images of blastocysts in young and young+*B.ca* groups. Scale bar, 80 μm. (**L**) The blastocyst formation rate was quantified in young ($n = 115$) and young+*B.ca* ($n = 84$) groups. **$P = 0.0041$. Data in (**B**), (**C**), (**D**), (**E**), (**G**), (**H**), (**J**), and (**L**) were presented as mean ± SEM or SD of at least three independent experiments. *$P < 0.05$; **$P < 0.01$; ***$P < 0.001$; ****$P < 0.0001$. Statistical significance was determined by two-tailed unpaired t-test.

© The Author(s)

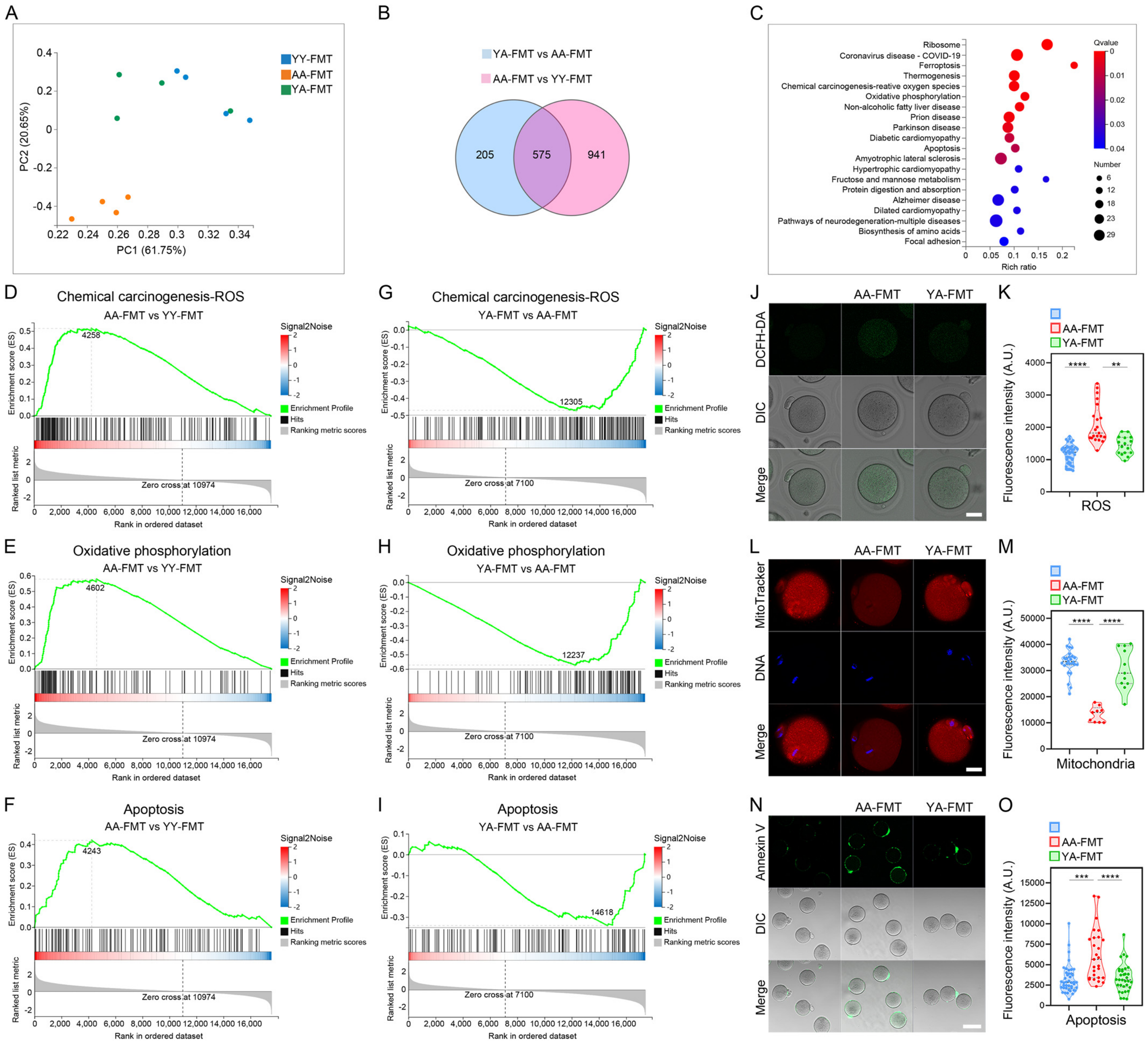

Figure EV6. Supplementary transcriptome data related to Fig. 5.

(**A**) PCA plot displayed the oocyte samples from YY-FMT, AA-FMT, and YA-FMT mice. Blue circles represented samples from YY-FMT group, orange circles represented samples from AA-FMT group, and green circles represented samples from YA-FMT group. (**B**) Venn diagram showed the number of overlapping differential transcripts between AA-FMT compared to YY-FMT group and YA-FMT compared to AA-FMT group. (**C**) KEGG enrichment analysis of overlapping differential transcripts between AA-FMT compared to YY-FMT group and YA-FMT compared to AA-FMT group. (**D**) GSEA plot showed genes involved in the ROS pathway (AA-FMT positively correlated; YY-FMT negatively correlated). (**E**) GSEA plot showed genes involved in the oxidative phosphorylation pathway (AA-FMT positively correlated; YY-FMT negatively correlated). (**F**) GSEA plot showed genes involved in the apoptosis pathway (AA-FMT positively correlated; YY-FMT negatively correlated). (**G**) GSEA plot showed genes involved in the ROS pathway (YA-FMT positively correlated; AA-FMT negatively correlated). (**H**) GSEA plot showed genes involved in the oxidative phosphorylation pathway (YA-FMT positively correlated; AA-FMT negatively correlated). (**I**) GSEA plot showed genes involved in the apoptosis pathway (YA-FMT positively correlated; AA-FMT negatively correlated). (**J**) Representative images of ROS levels as detected by DCFH-DA staining in YY-FMT, AA-FMT, and YA-FMT oocytes. Scale bar, 20 μm. (**K**) The fluorescence intensity of ROS signals was quantified in YY-FMT ($n = 45$), AA-FMT ($n = 21$), and YA-FMT ($n = 16$) oocytes. $^{****}P < 0.0001$, $^{**}P = 0.0034$. (**L**) Representative images of mitochondria as stained with MitoTracker Red in YY-FMT, AA-FMT, and YA-FMT oocytes. Scale bar, 20 μm. (**M**) The fluorescence intensity of mitochondrial signals was measured in YY-FMT ($n = 26$), AA-FMT ($n = 9$), and YA-FMT ($n = 11$) oocytes. $^{****}P < 0.0001$, $^{****}P < 0.0001$. (**N**) Representative images of apoptotic oocytes as stained with Annexin-V in YY-FMT, AA-FMT, and YA-FMT groups. Scale bar, 80 μm. (**O**) The fluorescence intensity of Annexin-V signals on the membrane was quantified in YY-FMT ($n = 42$), AA-FMT ($n = 27$), and YA-FMT ($n = 33$) oocytes. $^{***}P = 0.0003$, $^{****}P < 0.0001$. Data in (**K**), (**M**), and (**O**) were presented as mean ± SD of at least three independent experiments. $^{**}P < 0.01$; $^{***}P < 0.001$; $^{****}P < 0.0001$. Statistical significance was determined by two-tailed unpaired t-test.

 © The Author(s)

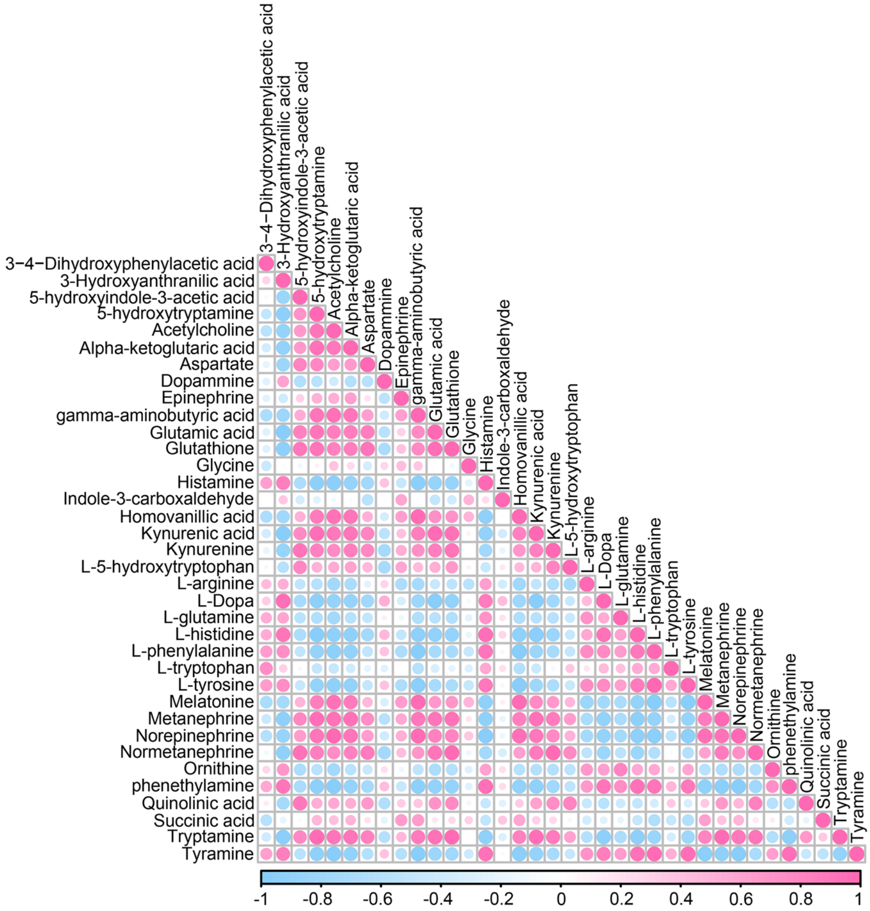

Figure EV7. Spearman's correlation analysis of neurotransmitter metabolites.

Red represented positive correlation, and blue represented negative correlation. The darker the color, the stronger the correlation.

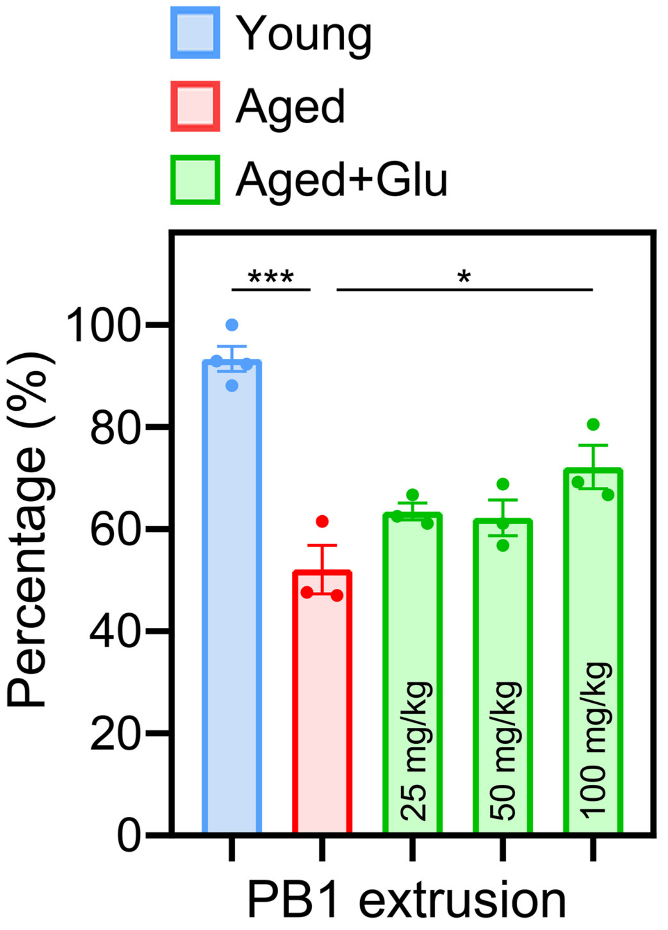

Figure EV8. Effects of different concentrations of glutamic acid on the maturation of aged oocytes.

The percentage of PB1 extrusion was quantified in young ($n = 222$), aged ($n = 51$), and aged+Glu (25 mg/kg: $n = 38$, 50 mg/kg: $n = 71$, 100 mg/kg: $n = 88$) oocytes. ***$P = 0.0004$, *$P = 0.0344$. Data were presented as mean ± SEM of at least three independent experiments. *$P < 0.05$; ***$P < 0.001$. Statistical significance was determined by two-tailed unpaired t-test.

© The Author(s)

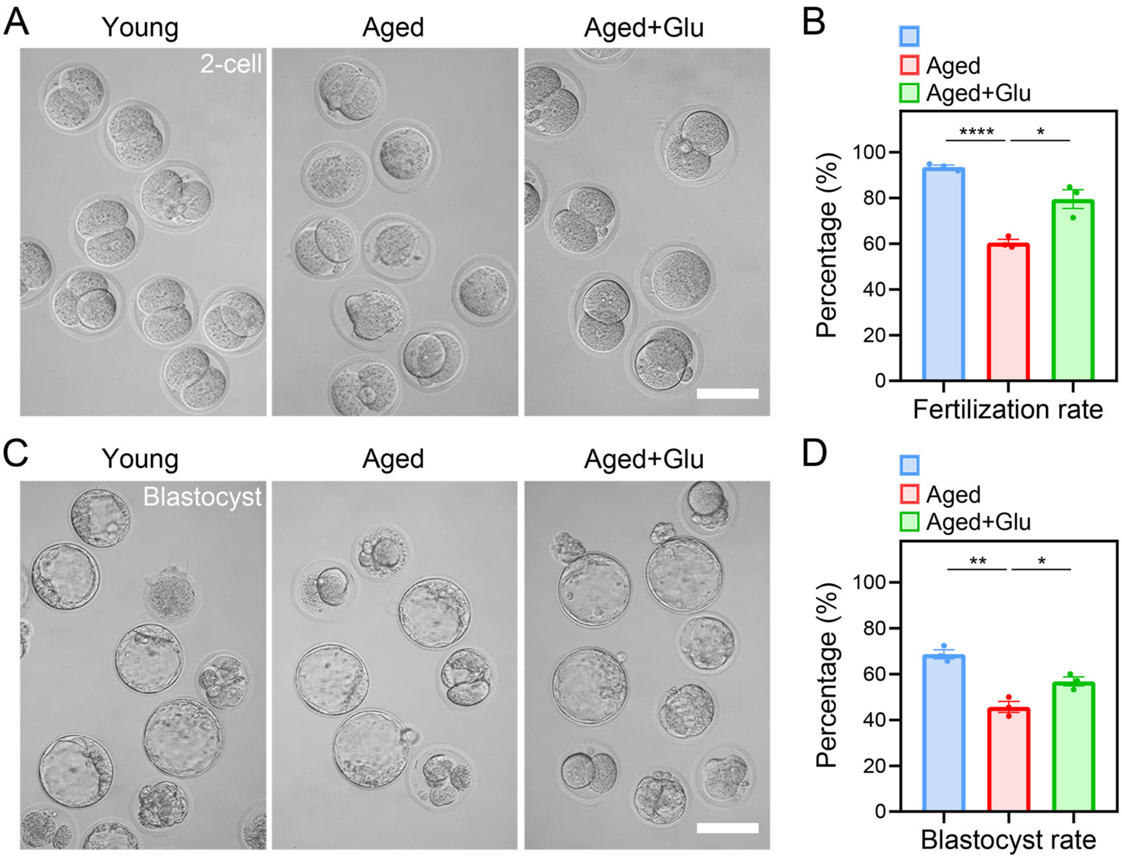

Figure EV9. Effects of glutamic acid supplementation on the fertilization and early embryonic development in aged mice.

(**A**) Representative images of 2-cell embryos in young, aged, and aged+Glu groups. Scale bar, 80 μm. (**B**) The fertilization rate was quantified in young ($n = 128$), aged ($n = 96$), and aged+Glu ($n = 78$) groups. ****$P < 0.0001$, *$P = 0.0120$. (**C**) Representative images of blastocysts in young, aged, and aged+Glu groups. Scale bar, 80 μm. (**D**) The blastocyst formation rate was quantified in young ($n = 143$), aged ($n = 58$), and aged+Glu ($n = 48$) groups. **$P = 0.0018$, *$P = 0.0227$. Data in (**B**) and (**D**) were presented as mean ± SEM of at least three independent experiments. *$P < 0.05$; **$P < 0.01$; ****$P < 0.0001$. Statistical significance was determined by two-tailed unpaired t-test.

© The Author(s)

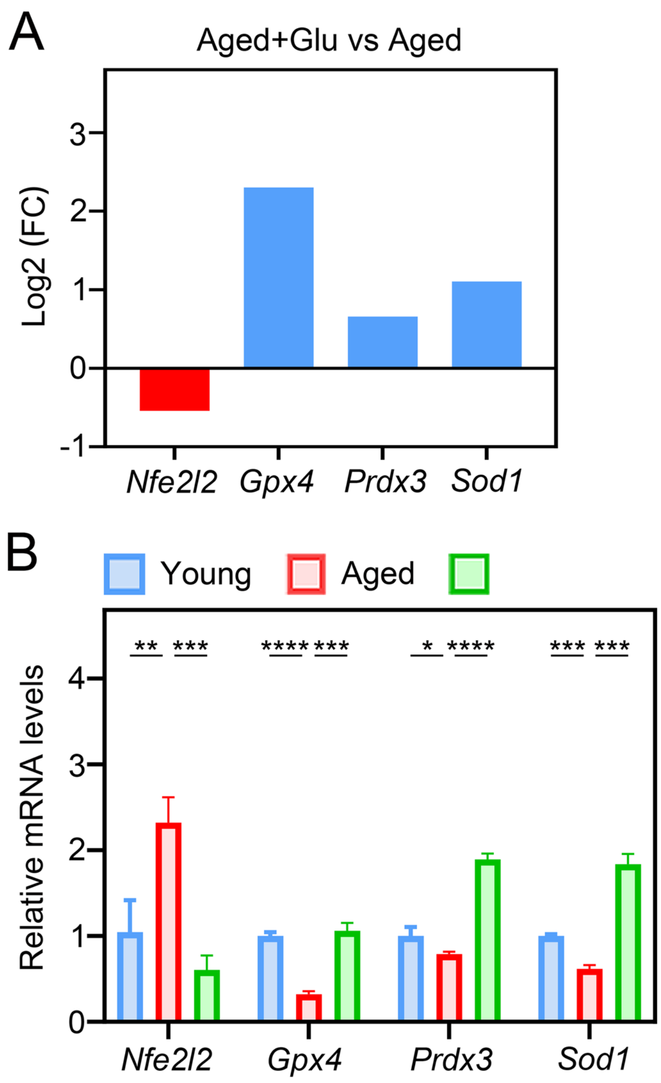

Figure EV10. Effects of glutamic acid supplementation on the transcript levels of some antioxidant genes.

(**A**) RNA-seq results of selected antioxidant genes in aged+Glu oocytes compared to aged ones. (**B**) Validation of RNA-seq data by quantitative RT-PCR in young ($n = 60$), aged ($n = 60$), and aged+Glu ($n = 60$) oocytes. $^{**}P = 0.0065$, $^{***}P = 0.0009$, $^{****}P < 0.0001$, $^{***}P = 0.0002$, $^{*}P = 0.0131$, $^{****}P < 0.0001$, $^{***}P = 0.0002$, $^{***}P = 0.0002$. Data in (**B**) were presented as mean ± SEM of at least three independent experiments. $^{*}P < 0.05$; $^{**}P < 0.01$; $^{***}P < 0.001$; $^{****}P < 0.0001$. Statistical significance was determined by multiple t-test.

 © The Author(s)

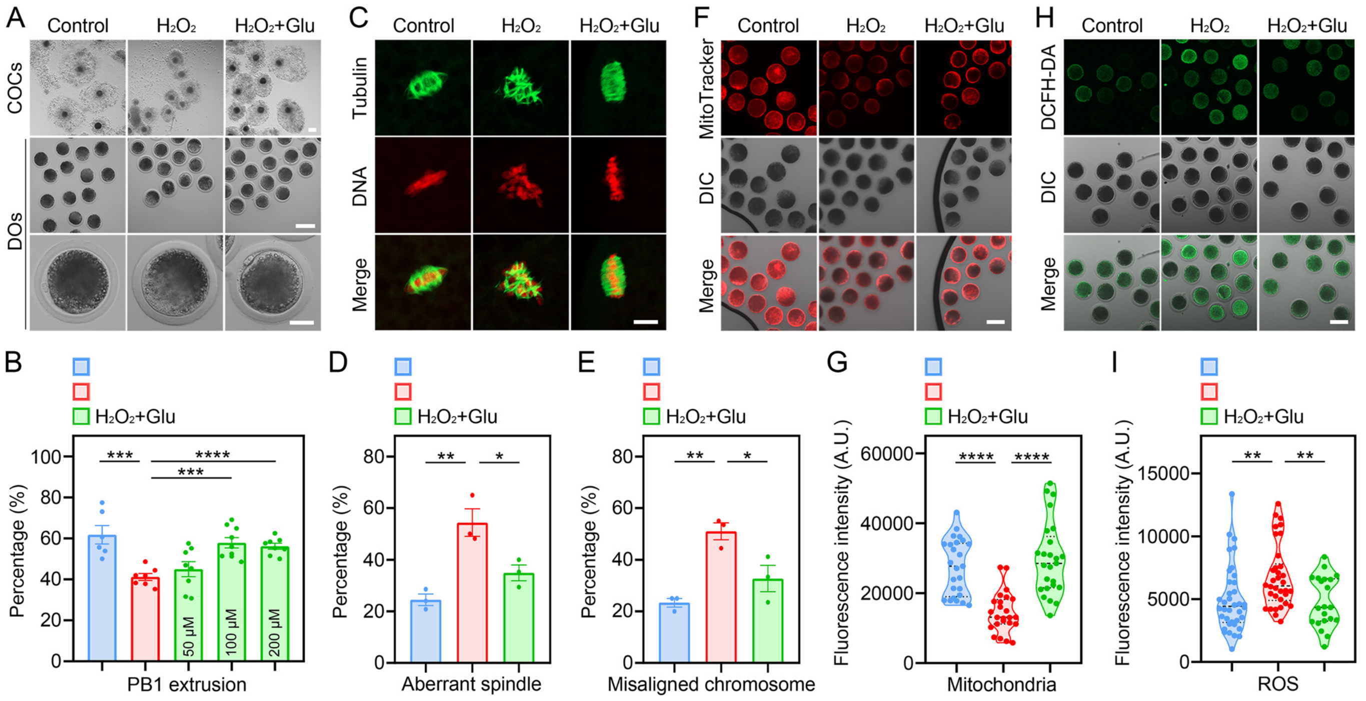

Figure EV11. Effects of glutamic acid supplementation on the quality of porcine oocytes treated with H_2O_2.

(**A**) Representative images of in vitro matured porcine oocytes in control, H_2O_2-treated, and Glu-supplemented groups. Scale bars, 120 μm, 120 μm, 30 μm. GV oocytes were treated with 100 μM H_2O_2 for 45 min and then cultured in the fresh medium with or without glutamic acid for in vitro maturation. (**B**) The rate of PB1 extrusion was recorded in control ($n = 216$), H_2O_2-treated ($n = 351$), and different concentrations of Glu-supplemented groups (50 μM: $n = 365$, 100 μM: $n = 339$; 200 μM: $n = 302$) after in vitro culture for 44 h. 200 μM Glu was used for subsequent analyses. ***$P = 0.0009$, ***$P = 0.0002$, ****$P < 0.0001$. (**C**) Representative images of spindle morphology and chromosome alignment in control, H_2O_2-treated, and Glu-supplemented oocytes. Scale bar, 5 μm. (**D**) The rate of abnormal spindles was measured in control ($n = 77$), H_2O_2-treated ($n = 75$), and Glu-supplemented ($n = 49$) oocytes. **$P = 0.0067$, *$P = 0.0340$. (**E**) The rate of misaligned chromosomes was measured in control ($n = 77$), H_2O_2-treated ($n = 75$), and Glu-supplemented ($n = 49$) oocytes. **$P = 0.0017$, *$P = 0.0395$. (**F**) Representative images of mitochondrial signals as shown by MitoTracker staining in control, H_2O_2-treated, and Glu-supplemented oocytes. Scale bar, 120 μm. (**G**) The fluorescence intensity of mitochondrial signals was measured in control ($n = 25$), H_2O_2-treated ($n = 24$), and Glu-supplemented ($n = 25$) oocytes. ****$P < 0.0001$, ****$P < 0.0001$. (**H**) Representative images of ROS levels as detected by DCFH-DA staining in control, H_2O_2-treated, and Glu-supplemented oocytes. Scale bar, 120 μm. (**I**) The fluorescence intensity of ROS signals was measured in control ($n = 38$), H_2O_2-treated ($n = 34$), and Glu-supplemented ($n = 21$) oocytes. **$P = 0.0033$, **$P = 0.0034$. Data in (**B**), (**D**), (**E**), (**G**), and (**I**) were presented as mean ± SEM or SD of at least three independent experiments. *$P < 0.05$; **$P < 0.01$; ***$P < 0.001$; ****$P < 0.0001$. Statistical significance was determined by two-tailed unpaired t-test.

© The Author(s)

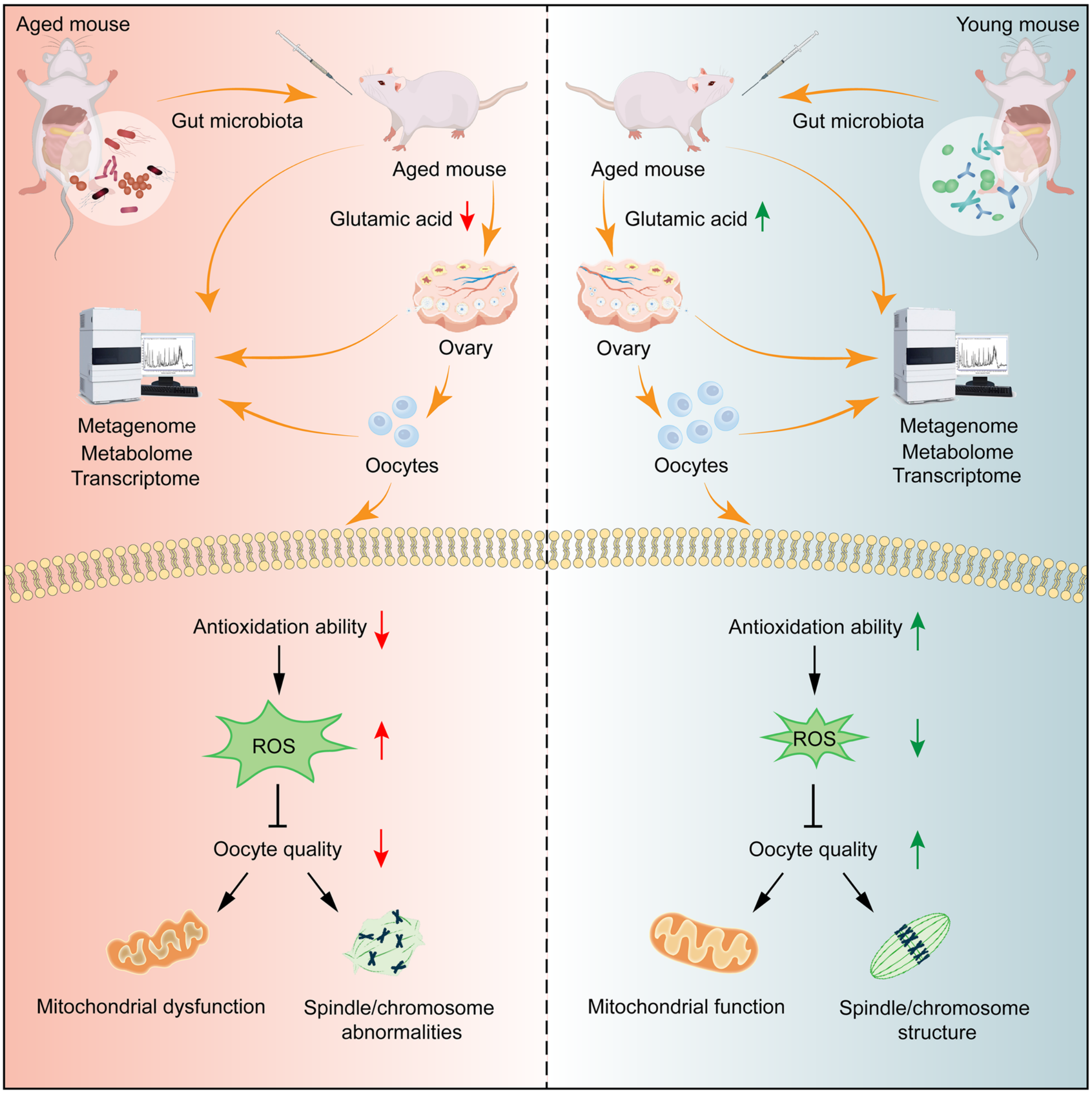

Figure EV12. Working model regarding how young gut microbiota rejuvenate the quality of maternally aged oocytes.

 © The Author(s)
