# Supplementary material for: Gut microbiota-modulated glutamic acid rejuvenates the quality of oocytes deteriorated by advanced reproductive age
Source: EMBO Mol Med. 2026 May 8;18(6):2404–35. doi: 10.1038/s44321-026-00443-3 (PMC13270145; doi:10.1038/s44321-026-00443-3)
Supplement: Supplementary file 2 — Peer Review File [file 44321_2026_443_MOESM2_ESM.pdf]

EMBO Molecular Medicine

Gut microbiota-modulated glutamic acid rejuvenates the quality of oocytes deteriorated by advanced reproductive age

Feixue Wang, Wenjun Zeng, Zihao Zhang, Na Li, Zhaokang Cui, Jie Bai, Jiner Yan, Yu Zhang, Yilong Miao, Ling Gu, and Bo Xiong

Corresponding author(s): Bo Xiong (xiongbo@zju.edu.cn)

Review Timeline:

Submission Date:	18th Sep 25
Editorial Decision:	23rd Oct 25
Revision Received:	18th Mar 26
Editorial Decision:	20th Apr 26
Revision Received:	24th Apr 26
Accepted:	28th Apr 26

Editor: Lise Roth

Transaction Report:

(Note: With the exception of the correction of typographical or spelling errors that could be a source of ambiguity, letters and reports are not edited. Depending on transfer agreements, referee reports obtained elsewhere may or may not be included in this compilation. Referee reports are anonymous unless the Referee chooses to sign their reports.)

23rd Oct 2025

Dear Prof. Xiong,

Thank you for submitting your manuscript to EMBO Molecular Medicine, and please accept my apologies for the delay in getting back to you, as we were waiting for a referee report. Unfortunately, Referee #2 will not be able to provide a report due to unforeseen personal circumstances. However, we have received feedback from Referees #1 and #3, who make similar recommendations. We would therefore like to make a decision now, in order to avoid further delay to the process. As you will see from the reports below, the reviewers recognise the interest of the study and generally support the publication of your work, pending appropriate revisions.

Fully addressing the reviewers' concerns will be necessary for us to consider the manuscript further, and acceptance will entail a second round of review. EMBO Molecular Medicine only encourages a single round of revisions, so acceptance or rejection of the manuscript will depend on how complete your responses are in the final version. To avoid any frustration later on, I would strongly advise against submitting an incomplete revision.

If you would like to discuss further the points raised by the referees, I am available to do so via email or video. Let me know if you are interested in this option.

We are expecting your revised manuscript within three months, if you anticipate any delay, please contact us.

When submitting your revised manuscript, please carefully review the instructions that follow below. We perform an initial quality control of all revised manuscripts before re-review; failure to include requested items will delay the evaluation of your revision.

We require:

1) A .docx formatted version of the manuscript text (including legends for main figures, EV figures and tables). Please make sure that the changes are highlighted to be clearly visible.

2) Individual production quality figure files as .eps, .tif, .jpg (one file per figure). For guidance, download the 'Figure Guide PDF' (https://www.embopress.org/page/journal/17574684/authorguide#figureformat).

3) At EMBO Press we ask authors to provide source data for the main manuscript figures. You will receive a separate email with instructions for providing source data with your revised manuscript, including how to upload and organize the files.

Additional information on source data and instruction on how to label the files are available

4) A .docx formatted letter INCLUDING the reviewers' reports and your detailed point-by-point responses to their comments. As part of the EMBO Press transparent editorial process, the point-by-point response is part of the Review Process File (RPF), which will be published alongside your paper.

5) A complete author checklist, which you can download from our author guidelines (https://www.embopress.org/page/journal/17574684/authorguide#submissionofrevisions). Please insert information in the checklist that is also reflected in the manuscript. The completed author checklist will also be part of the RPF.

6) All Materials and Methods need to be described in the main text using our 'Structured Methods' format. According to this format, the Methods section includes a Reagents and Tools Table (listing key reagents, experimental models, software and relevant equipment and including their sources and relevant identifiers) followed by a Methods and Protocols section describing the methods, ideally using a step-by-step protocol format. The aim is to facilitate adoption of the methodologies across labs. Please download and fill our Reagents and Tools Table template (.docx), which you can find in our author guidelines: https://www.embopress.org/page/journal/14693178/authorguide#structuredmethods.
When submitting your revised manuscript, please do not include the Reagents and Tools Table in the Methods section of the manuscript but upload it as a separate file choosing the file type "Reagent Table".

7) Please note that all corresponding authors are required to supply an ORCID ID for their name upon submission of a revised manuscript.

8) It is mandatory to include a 'Data Availability' section after the Materials and Methods. Before submitting your revision, primary datasets produced in this study need to be deposited in an appropriate public database, and the accession numbers and database listed under 'Data Availability'. Please remember to provide a reviewer password if the datasets are not yet public (see https://www.embopress.org/page/journal/17574684/authorguide#dataavailability).

In case you have no data that requires deposition in a public database, please state so in this section. Note that the Data Availability Section is restricted to new primary data that are part of this study.

9) For data quantification: please specify the name of the statistical test used to generate error bars and P values, the number (n) of independent experiments (specify technical or biological replicates) underlying each data point and the test used to calculate p-values in each figure legend. The figure legends should contain a basic description of n, P and the test applied. Graphs must include a description of the bars and the error bars (s.d., s.e.m.). Please provide exact p values.

10) Our journal encourages inclusion of *data citations in the reference list* to directly cite datasets that were re-used and obtained from public databases. Data citations in the article text are distinct from normal bibliographical citations and should directly link to the database records from which the data can be accessed. In the main text, data citations are formatted as follows: "Data ref: Smith et al, 2001" or "Data ref: NCBI Sequence Read Archive PRJNA342805, 2017". In the Reference list, data citations must be labeled with "[DATASET]". A data reference must provide the database name, accession number/identifiers and a resolvable link to the landing page from which the data can be accessed at the end of the reference. Further instructions are available at .

11) We replaced Supplementary Information with Expanded View (EV) Figures and Tables that are collapsible/expandable online. EV Figures should be cited as 'Figure EV1, Figure EV2" etc... in the text and their respective legends should be included in the main text after the legends of regular figures.
- For the figures that you do NOT wish to display as Expanded View figures, they should be bundled together with their legends in a single PDF file called *Appendix*, which should start with a short Table of Content. Appendix figures should be referred to in the main text as: "Appendix Figure S1, Appendix Figure S2" etc.
- Additional Tables/Datasets should be labeled and referred to as Table EV1, Dataset EV1, etc. Legends have to be provided in a separate tab in case of .xls files. Alternatively, the legend can be supplied as a separate text file (README) and zipped together with the Table/Dataset file.
See detailed instructions here:

12) The paper explained: EMBO Molecular Medicine articles are accompanied by a summary of the articles to emphasize the major findings in the paper and their medical implications for the non-specialist reader. Please provide a draft summary of your article highlighting
- the medical issue you are addressing,
- the results obtained and
- their clinical impact.
This may be edited to ensure that readers understand the significance and context of the research. Please refer to any of our published articles for an example.

13) Author contributions: CRediT has replaced the traditional author contributions section because it offers a systematic machine readable author contributions format that allows for more effective research assessment. Please remove the Authors Contributions from the manuscript and use the free text boxes beneath each contributing author's name in our system to add specific details on the author's contribution. More information is available in our guide to authors.

14) Disclosure statement and competing interests: We updated our journal's competing interests policy in January 2022 and request authors to consider both actual and perceived competing interests. Please review the policy https://www.embopress.org/competing-interests and update your competing interests if necessary.

15) Every published paper now includes a 'Synopsis' to further enhance discoverability. Synopses are displayed on the journal webpage and are freely accessible to all readers. They include a short stand first (maximum of 300 characters, including space) as well as 2-5 one-sentences bullet points that summarizes the paper. Please write the bullet points to summarize the key NEW findings. They should be designed to be complementary to the abstract - i.e. not repeat the same text. We encourage inclusion of key acronyms and quantitative information (maximum of 30 words / bullet point). Please use the passive voice. Please attach these in a separate file or send them by email, we will incorporate them accordingly.

Please also suggest a visual abstract to illustrate your article as a PNG file 550 px wide x 300-600 px high. A cropped portion of this image will serve as thumbnail for the table of content on our webpage.

16) As part of the EMBO Publications transparent editorial process initiative (see our Editorial at http://embomolmed.embopress.org/content/2/9/329), EMBO Molecular Medicine will publish online a Review Process File (RPF) to accompany accepted manuscripts.
In the event of acceptance, this file will be published in conjunction with your paper and will include the anonymous referee reports, your point-by-point response and all pertinent correspondence relating to the manuscript. Let us know whether you agree with the publication of the RPF and as here, if you want to remove or not any figures from it prior to publication.
Please note that the Authors checklist will be published at the end of the RPF.

EMBO Molecular Medicine has a "scooping protection" policy, whereby similar findings that are published by others during review or revision are not a criterion for rejection. Should you decide to submit a revised version, I do ask that you get in touch after three months if you have not completed it, to update us on the status.

I look forward to receiving your revised manuscript.

Yours sincerely,

Lise Roth

Lise Roth, PhD
Senior Editor
EMBO Molecular Medicine

***** Reviewer's comments *****

Referee #1 (Remarks for Author):

In the present maunscript, Wang et al. provided valuable insights into the mechanisms underlying how FMT from young donors restore the quality of oocytes and female fertility in aged mice from microbiota and metabolic aspects. Briefly, by integrating multi-omics including metagenome, untargeted metabolome, targeted metabolome, and micro-transcriptome analyses, they revealed that Bacteroides_caecimuris modulated glutamic acid levels mediated
the favorable impacts of young gut microbiota on the aged oocytes through enhancing the mitochondria function and anti-oxidation system. They also validated that supplementation of glutamic acid phenocopied the FMT effects. Overall, this is an interesting study which significantly enhances our understanding of the importance of gut microbiota in the oocyte aging, and has broader implications for reproductive medicine. However, several additional experiments and a revised discussion are recommended before publication.

Major points:

1. For the action of glutamic acid on the oocytes, authors mentioned that glutamic acid can act as a synthetic precursor of glutathione that reduces oxidative stress and maintains redox balance in cells, but they did not provide data to evidence it. Imonobromobimane (mBBr) staining is encouraged to perform to indicate the glutathione levels in young, aged and aged+Glu oocytes.
2. The micro-transcriptome analysis reveals the differential transcripts between aged and aged+Glu oocytes, with a heatmap displaying the global transcriptomic profile. The authors did not further elaborate on how the transcript levels of specific genes relevant to antioxidation response is altered. Thus, it is recommended to compare the transcript levels of several important anti-oxidation genes from RNA-seq data, and verify them by RT-PCR to determine whether the recovey of aged oocyte qualiy by glutamic acid involves anti-oxidation system.
3. Supplementation with Bacteroides caecimuris reduced ovarian glutamate levels in young mice, supporting a negative association between this strain and glutamate. To enhance the mechanistic understanding, the authors should consider providing additional experiments or citing relevant literature to clarify how B. caecimuris modulates ovarian glutamate levels-whether through the metabolism of glutamate into other compounds, the regulation of key enzymes involved in glutamate synthesis, or other potential pathways. Direct experimental validation or a more comprehensive discussion is necessary to address this point convincingly.
4. The authors report that 30-day FMT in young mice did not improve oocyte quality in aged mice. It is recommended that the authors perform longitudinal gut microbiota sequencing at day 15 and day 30 to analyze dynamic changes in microbial composition, which may help explain the time-limited efficacy. Additionally, the abundance of B. caecimuris and ovarian glutamate levels at day 30 should be examined.

Minor points:

1. In Fig. 7A, the information regarding the timing of hormone injections in the flow chart is suggested to include.

2. In Fig. S3, a clear schematic illustration for the specific experimental timeline is recommended to provide to properly assess the duration of the FMT effects.

3. Please clarify how the precedures used for antibiotic treatment and bacterial colonization are determined in the Materials and Methods section. If this references the previous literature, please cite them.

4. Please include the discussion about the limitation of this study in the Discussion section.

5. In the Materials and Methods section, authors stated that live Bacteroides strains were purchased from GemPharmatech. The more detailed information about this bacterial such as catalog number or strain identifiers are suggested to include.

Referee #3 (Comments on Novelty/Model System for Author):

The study by Wang and Xiong used multi-omics and reproductively aged mouse model to investigate the potential roles of gut microbiota and their metabolites in the maintenance of oocyte quality during maternal aging. Authors showed that fecal microbiota transplantation from young donors could effectively enhance the oocyte quality and fertility of reproductively aged mice. Importantly, by jointly analyzing the metagenome and untargeted metabolome of intestinal digesta, as well as targeted metabolome of ovaries and transcriptome of oocytes, they discovered that Bacteroides_caecimuris-modulated glutamic acid levels might mediate the rejuvenated roles of young gut microbiota on the aged oocytes, and supplementation of glutamic acid also improved the oocyte quality in aged mice. In sum, this is an intriguing and technically well done work which will have a significant translational impact. Thus, in principal, I am strongly in favor of publication.

Referee #3 (Remarks for Author):

The study by Wang and Xiong used multi-omics and reproductively aged mouse model to investigate the potential roles of gut microbiota and their metabolites in the maintenance of oocyte quality during maternal aging. Authors showed that fecal microbiota transplantation from young donors could effectively enhance the oocyte quality and fertility of reproductively aged mice. Importantly, by jointly analyzing the metagenome and untargeted metabolome of intestinal digesta, as well as targeted metabolome of ovaries and transcriptome of oocytes, they discovered that Bacteroides_caecimuris-modulated glutamic acid levels might mediate the rejuvenated roles of young gut microbiota on the aged oocytes, and supplementation of glutamic acid also improved the oocyte quality in aged mice. In sum, this is an intriguing and technically well done work which will have a significant translational impact. Thus, in principal, I am strongly in favor of publication, however the authors should consider the following points:

1. Fig. 2B and 7L, to present fertility data more intuitively, it is suggested to quantify the average number of pups produced per litter in each experimental group for comparison.

2. Fig 5B, C, E, it seems the coordinate axes for these charts were dislocated, please properly correct them to the right place.

3. Fig. 7A, the schematic diagram for glutamic acid supplementation lacks of time points for PMSG and hCG injections.

4. Fig. 8B, it is not clear which color represents the up-regulated transcripts and which represents down-regulated ones in the volcano plot. Please present these data just like Fig. 3B for clarity.

5. Fig. S9A, the embryos in the 'Aged+Glu' groups look bigger than those in other two groups, please adjust them to the same scale.

6. Discussion, authors are suggested to discuss a recent study reporting an important role of microbiota in extending the reproductive lifespan of mice by safeguarding the ovarian reserve (PMID: 41005310).

7. Materials and Methods, it is not clear why authors gavaged the fecal suspension on days 4 to 8, and then gave it again after one week.

8. Figure 5 and 8 legends, in fully-grown mouse oocytes, the transcription is silenced, so the description of DEGs (differentially expressed genes) is not correct here. Please revise them to 'differential transcripts' as described in the text of Results.

Point-by-point response

Referee #1:

In the present manuscript, Wang et al. provided valuable insights into the mechanisms underlying how FMT from young donors restore the quality of oocytes and female fertility in aged mice from microbiota and metabolic aspects. Briefly, by integrating multi-omics including metagenome, untargeted metabolome, targeted metabolome, and micro-transcriptome analyses, they revealed that Bacteroides_caecimuris modulated glutamic acid levels mediated

the favorable impacts of young gut microbiota on the aged oocytes through enhancing the mitochondria function and anti-oxidation system. They also validated that supplementation of glutamic acid phenocopied the FMT effects. Overall, this is an interesting study which significantly enhances our understanding of the importance of gut microbiota in the oocyte aging, and has broader implications for reproductive medicine. However, several additional experiments and a revised discussion are recommended before publication.

Major points:

1. For the action of glutamic acid on the oocytes, authors mentioned that glutamic acid can act as a synthetic precursor of glutathione that reduces oxidative stress and maintains redox balance in cells, but they did not provide data to evidence it. Imonobromobimane (mBBr) staining is encouraged to perform to indicate the glutathione levels in young, aged and aged+Glu oocytes.

Response: We thank the reviewer very much for the constructive suggestions to improve our manuscript. As suggested, we performed mBBr staining to assess intracellular glutathione (GSH) levels in young, aged, and aged+Glu oocytes. The results showed that GSH levels were significantly reduced in aged oocytes compared with young controls, whereas glutamic acid supplementation markedly restored intracellular GSH levels in aged oocytes. These findings further support the hypothesis that glutamic acid improves oocyte quality by enhancing glutathione and maintaining redox balance. The new data have been added in Fig. 8E, F.

2. The micro-transcriptome analysis reveals the differential transcripts between aged and aged+Glu oocytes, with a heatmap displaying the global transcriptomic profile. The authors did not further elaborate on how the transcript levels of specific genes relevant to antioxidation response is altered. Thus, it is recommended to compare the transcript levels of several important anti-oxidation genes from RNA-seq data, and verify them by RT-PCR to determine whether the recovery of aged oocyte quality by glutamic acid involves anti-oxidation system.

Response: We thank the reviewer for this important suggestion. As suggested, we performed RNA-seq analysis followed by qRT-PCR validation, showing that glutamic acid supplementation restored the transcript levels of several key antioxidant genes in aged oocytes. The new data have been added in Fig. S10.

3. Supplementation with Bacteroides caecimuris reduced ovarian glutamate levels in young mice, supporting a negative association between this strain and glutamate. To enhance the mechanistic understanding, the authors should consider providing additional experiments or citing relevant literature to clarify how B. caecimuris modulates ovarian glutamate levels-whether through the metabolism of glutamate into other compounds, the regulation of key enzymes involved in glutamate synthesis, or other potential pathways. Direct experimental validation or a more comprehensive discussion is necessary to address this point convincingly.
Response: We appreciate the reviewer's suggestion to clarify how B. caecimuris modulates ovarian glutamate levels. Through analysis using EnsemblBacteria, we identified that B. caecimuris encodes glutamate decarboxylase (GAD). Moreover, supplementation with this bacterium increased ovarian GAD protein levels, suggesting enhanced glutamate consumption. These findings provide a plausible enzymatic mechanism underlying the reduction in ovarian glutamate levels. The corresponding data have now been included in Fig. 4H-J.

4. The authors report that 30-day FMT in young mice did not improve oocyte quality in aged mice. It is recommended that the authors perform longitudinal gut microbiota sequencing at day 15 and day 30 to analyze dynamic changes in microbial composition, which may help explain the time-limited efficacy. Additionally, the abundance of B. caecimuris and ovarian glutamate levels at day 30 should be examined.
Response: We thank the reviewer for this important suggestion. To address this, we performed longitudinal 16S rRNA sequencing at day 15 and day 30 post-FMT. The results showed that Bacteroides composition in young-to-aged FMT mice at day 30 resembled that of aged mice, whereas at day 15 it had not yet shifted. Furthermore, analysis of B. caecimuris abundance and ovarian glutamate levels at day 30 indicated no significant difference from the aged group. These data suggest that the limited efficacy of 30-day FMT is associated with the incomplete maintenance of young microbiota over time. The new data have now been included in Fig. S3H, I, P, Q, R, S.

Minor points:
1. In Fig. 7A, the information regarding the timing of hormone injections in the flow chart is suggested to include.
Response: As suggested, we have added the timing of hormone injections in the flow chart in Fig. 7A.

2. In Fig. S3, a clear schematic illustration for the specific experimental timeline is recommended to provide to properly assess the duration of the FMT effects.
Response: As suggested, a schematic diagram of the experimental timeline has been provided in the Fig. S3A.

3. Please clarify how the procedures used for antibiotic treatment and bacterial

colonization are determined in the Materials and Methods section. If this references the previous literature, please cite them.
Response: The procedures for antibiotic treatment and bacterial colonization were performed according to the referenced papers and we have provided the corresponding citations as shown in line 459 and 465.

4. Please include the discussion about the limitation of this study in the Discussion section.
Response: We have discussed the limitation of the study in line 431.

5. In the Materials and Methods section, authors stated that live Bacteroides strains were purchased from GemPharmatech. The more detailed information about this bacterial such as catalog number or strain identifiers are suggested to include.
Response: We have added the information in line 502 as suggested.

Referee #3:
The study by Wang and Xiong used multi-omics and reproductively aged mouse model to investigate the potential roles of gut microbiota and their metabolites in the maintenance of oocyte quality during maternal aging. Authors showed that fecal microbiota transplantation from young donors could effectively enhance the oocyte quality and fertility of reproductively aged mice. Importantly, by jointly analyzing the metagenome and untargeted metabolome of intestinal digesta, as well as targeted metabolome of ovaries and transcriptome of oocytes, they discovered that Bacteroides_caecimuris-modulated glutamic acid levels might mediate the rejuvenated roles of young gut microbiota on the aged oocytes, and supplementation of glutamic acid also improved the oocyte quality in aged mice. In sum, this is an intriguing and technically well done work which will have a significant translational impact. Thus, in principal, I am strongly in favor of publication, however the authors should consider the following points:

1. Fig. 2B and 7L, to present fertility data more intuitively, it is suggested to quantify the average number of pups produced per litter in each experimental group for comparison.
Response: We thank the reviewer very much for the constructive suggestions to improve our manuscript. As suggested, we have presented the fertility data by quantifying the average number of pups per litter as shown in Fig. 2B, C and 7L, M.

2. Fig 5B, C, E, it seems the coordinate axes for these charts were dislocated, please properly correct them to the right place.
Response: We thank the reviewer for carefully examining the figures and pointing out this issue. The coordinate axes in Fig. 5B, C, E were inadvertently displaced during figure assembly. We have now corrected the positioning of the axes to ensure proper alignment and clarity in the revised manuscript.

3. Fig. 7A, the schematic diagram for glutamic acid supplementation lacks of time points for PMSG and hCG injections.
Response: As suggested, we have added this information in the Fig. 7A.

4. Fig. 8B, it is not clear which color represents the up-regulated transcripts and which represents down-regulated ones in the volcano plot. Please present these data just like Fig. 3B for clarity.
Response: As suggested, we have now revised Fig. 8B to explicitly indicate which color represents up-regulated transcripts and which represents down-regulated transcripts, consistent with the presentation format used in Fig. 3B.

5. Fig. S9A, the embryos in the 'Aged+Glu' groups look bigger than those in other two groups, please adjust them to the same scale.
Response: As suggested, we have adjusted all panels to the same scale and magnification. The revised figure has been updated accordingly to ensure accurate visual comparison among groups.

6. Discussion, authors are suggested to discuss a recent study reporting an important role of microbiota in extending the reproductive lifespan of mice by safeguarding the ovarian reserve (PMID: 41005310).
Response: As suggested, we have discussed and cited this reference in the Discussion section as shown in line 377.

7. Materials and Methods, it is not clear why authors gavaged the fecal suspension on days 4 to 8, and then gave it again after one week.
Response: The FMT schedule used in our study was designed based on a previously published protocol. To improve clarity, we have now added a statement in the Materials and Methods section explicitly explaining that the FMT schedule was performed according to the referenced protocol and have provided the corresponding citation (line 465).

8. Figure 5 and 8 legends, in fully-grown mouse oocytes, the transcription is silenced, so the description of DEGs (differentially expressed genes) is not correct here. Please revise them to 'differential transcripts' as described in the text of Results.
Response: We have revised them as suggested.

20th Apr 2026

Dear Prof. Xiong,

Thank you for submitting your revised study, and please accept my apologies for the delay in getting back to you. Unfortunately, referee #2 was not available to review your revised manuscript, so I reached out to an advisor who reviewed your responses to this referee's concerns. Both Referee #3 and our adviser are satisfied with the revisions. We will therefore be able to accept your manuscript once you address the following minor editorial matters:

1/ Manuscript text:
- Please remove the red font text and only keep in track changes mode any new modification.
- Please update the headings of the sections in the manuscript text to: Abstract / The Paper Explained / Introduction / Results / Discussion / Methods / Data Availability / Acknowledgements / Disclosure and Competing Interests Statement / References / Figure Legends / Expanded View Figure Legends
- Methods:
o Animals: please provide the origin of the mice.
o Porcine oocyte collection and in vitro maturation: please provide more details, including origin of the animals, housing conditions, permit number, and all relevant information.
o Statistics: please provide a statement on sample size, inclusion/exclusion criteria, blinding and randomization.
- Data Availability: thank you for providing reviewer access for the deposited datasets, however we were not able to access or download the raw data. Please check.
- Acknowledgements: Funding Postgraduate Research & Practice Innovation Program of Jiangsu Province (KYCX25_1007) is missing in our submission system, please correct.
- Author contributions: Please remove from the manuscript text and provide CRediT (Contributor Role Taxonomy) terms in the submission system.
- Please rename "Conflict of interest" to "Disclosure and competing interests statement".

2/ Figures:
- EV figures should also be uploaded as individual, high resolution figure files, and the legends should be added to the manuscript text, after the main figure legends and under the heading "Expanded View Figure Legends".
- Table EV1 should be uploaded as a separate file.
- Legends should be added to the dataset files.
- Please address the queries from our data editors in the figure legends:
1. Please indicate the statistical test used for data analysis in the legends of figures 1F, G; 3B, C, D, E; 5B, C; 8B,
2. Please note that the box plots need to be defined in terms of minima, maxima, centre, bounds of box and whiskers, and percentile in the legend of figure 1D
3. Please note that information related to n is missing in the legends of figures 1D, 3B, C; 4E, F, J; 5B, C; 8B

3/ Please provide 'The paper explained': EMBO Molecular Medicine articles are accompanied by a summary of the articles to emphasize the major findings in the paper and their medical implications for the non-specialist reader. Please provide a draft summary of your article highlighting
- the medical issue you are addressing,
- the results obtained and
- their clinical impact.

This may be edited to ensure that readers understand the significance and context of the research. Please refer to any of our published articles for an example.

4/ Every published paper includes a 'Synopsis' to further enhance discoverability. Synopses are displayed on the journal webpage and are freely accessible to all readers. They include a short stand first (maximum of 300 characters, including space) as well as 2-5 one-sentences bullet points that summarizes the paper. Please write the bullet points to summarize the key NEW findings. They should be designed to be complementary to the abstract - i.e. not repeat the same text. We encourage inclusion of key acronyms and quantitative information (maximum of 30 words / bullet point). Please use the passive voice. Please attach these in a separate file or send them by email, we will incorporate them accordingly.

Please also suggest a striking image or visual abstract to illustrate your article as a PNG file 550 px wide x 300 px high. If you use an image data base for scientific iconography (e.g., BioRender), it should be indicated in the manuscript.

5/ As part of the EMBO Publications transparent editorial process initiative (see our Editorial at http://embomolmed.embopress.org/content/2/9/329), EMBO Molecular Medicine will publish online a Review Process File (RPF) to accompany accepted manuscripts.
This file will be published in conjunction with your paper and will include the anonymous referee reports, your point-by-point

response and all pertinent correspondence relating to the manuscript. Let us know whether you agree with the publication of the RPF.
Please note that the Authors checklist will be published at the end of the RPF.

I look forward to receiving your revised manuscript.

Yours sincerely,

Lise Roth

Lise Roth, PhD
Senior Editor
EMBO Molecular Medicine

***** Reviewer's comments *****

Referee #3 (Comments on Novelty/Model System for Author):

It is an adequate model for gut microbiota and aging study.

Referee #3 (Remarks for Author):

Authors have adequately addressed my previous concerns, and it is now suitable for publication.

Point-by-point response

Editorial matters:

1/ Manuscript text:

- Please remove the red font text and only keep in track changes mode any new modification.

Response: We have removed the red font text and kept in track changes mode for the new modification.

- Please update the headings of the sections in the manuscript text to: Abstract / The Paper Explained / Introduction / Results / Discussion / Methods / Data Availability / Acknowledgements / Disclosure and Competing Interests Statement / References / Figure Legends / Expanded View Figure Legends

Response: We have updated the headings of the sections in the manuscript text as requested.

- Methods:

o Animals: please provide the origin of the mice.

o Porcine oocyte collection and in vitro maturation: please provide more details, including origin of the animals, housing conditions, permit number, and all relevant information.

o Statistics: please provide a statement on sample size, inclusion/exclusion criteria, blinding and randomization.

Response: We have revised all above information in the Methods section as suggested.

- Data Availability: thank you for providing reviewer access for the deposited datasets, however we were not able to access or download the raw data. Please check.

Response: We have checked the links, and now make sure they work.

- Acknowledgements: Funding Postgraduate Research & Practice Innovation Program of Jiangsu Province (KYCX25_1007) is missing in our submission system, please correct.

Response: We have added it in the submission system.

- Author contributions: Please remove from the manuscript text and provide CRediT (Contributor Role Taxonomy) terms in the submission system.

Response: We have removed it as requested.

- Please rename "Conflict of interest" to "Disclosure and competing interests statement".

Response: We have revised it as requested.

2/ Figures:

- EV figures should also be uploaded as individual, high resolution figure files, and the legends should be added to the manuscript text, after the main figure legends and under the heading "Expanded View Figure Legends".
Response: We have uploaded them as individual files and moved the legends to the manuscript text as requested.

- Table EV1 should be uploaded as a separate file.
- Legends should be added to the dataset files.
Response: We have uploaded them as separate files and added the legends in the manuscript text.

- Please address the queries from our data editors in the figure legends:
1. Please indicate the statistical test used for data analysis in the legends of figures 1F, G; 3B, C, D, E; 5B, C; 8B,
2. Please note that the box plots need to be defined in terms of minima, maxima, centre, bounds of box and whiskers, and percentile in the legend of figure 1D
3. Please note that information related to n is missing in the legends of figures 1D, 3B, C; 4E, F, J; 5B, C; 8B
Response: We have provided all above information in the figure legends as requested.

3/ Please provide 'The paper explained': EMBO Molecular Medicine articles are accompanied by a summary of the articles to emphasize the major findings in the paper and their medical implications for the non-specialist reader. Please provide a draft summary of your article highlighting
- the medical issue you are addressing,
- the results obtained and
- their clinical impact.

This may be edited to ensure that readers understand the significance and context of the research. Please refer to any of our published articles for an example.
Response: We have included the 'The paper explained' in the manuscript text.

4/ Every published paper includes a 'Synopsis' to further enhance discoverability. Synopses are displayed on the journal webpage and are freely accessible to all readers. They include a short stand first (maximum of 300 characters, including space) as well as 2-5 one-sentences bullet points that summarizes the paper. Please write the bullet points to summarize the key NEW findings. They should be designed to be complementary to the abstract - i.e. not repeat the same text. We encourage inclusion of key acronyms and quantitative information (maximum of 30 words / bullet point). Please use the passive voice. Please attach these in a separate file or send them by email, we will incorporate them accordingly.
Please also suggest a striking image or visual abstract to illustrate your article as a PNG file 550 px wide x 300 px high. If you use an image data base for scientific

iconography (e.g., BioRender), it should be indicated in the manuscript.
Response: We have uploaded the 'Synopsis' and 'Visual abstract' as requested.

5/ As part of the EMBO Publications transparent editorial process initiative (see our Editorial at http://embomolmed.embopress.org/content/2/9/329), EMBO Molecular Medicine will publish online a Review Process File (RPF) to accompany accepted manuscripts.
This file will be published in conjunction with your paper and will include the anonymous referee reports, your point-by-point response and all pertinent correspondence relating to the manuscript. Let us know whether you agree with the publication of the RPF.
Please note that the Authors checklist will be published at the end of the RPF.
Response: We agree with publication of the RPF.

28th Apr 2026

Dear Prof. Xiong,

Thank you for submitting your revised files. I am pleased to inform you that your manuscript is accepted for publication and is now being sent to our publisher to be included in the next available issue of EMBO Molecular Medicine.

Your manuscript will be processed for publication by EMBO Press. It will be copy edited and you will receive page proofs prior to publication. Please note that you will be contacted by Springer Nature Author Services to complete licensing and payment information.

You may qualify for financial assistance for your publication charges - either via a Springer Nature fully open access agreement or an EMBO initiative. Check your eligibility: https://link.springer.com/journal/44321/how-to-publish-with-us

Should you be planning a Press Release on your article, please get in contact with embo_production@springernature.com as early as possible in order to coordinate publication and release dates.

If you have any questions, please do not hesitate to contact the Editorial Office. Thank you for your contribution to EMBO Molecular Medicine.

Yours sincerely,

Lise Roth

Lise Roth, Ph.D
Senior Editor
EMBO Molecular Medicine

>>> Please note that it is EMBO Molecular Medicine policy for the transcript of the editorial process (containing referee reports and your response letter) to be published as an online supplement to each paper. If you do NOT want this, you will need to inform the Editorial Office via email immediately. More information is available here: https://link.springer.com/partners/embo-press/editorial-policies#Peer%20review

EMBO Press Author Checklist

Corresponding Author Name: Bo Xiong
Journal Submitted to: EMBO Molecular Medicine
Manuscript Number: EMM-2025-22647

USEFUL LINKS FOR COMPLETING THIS FORM
The EMBO Journal - Author Guidelines
EMBO Reports - Author Guidelines
Molecular Systems Biology - Author Guidelines
EMBO Molecular Medicine - Author Guidelines

Reporting Checklist for Life Science Articles (updated January
This checklist is adapted from Materials Design Analysis Reporting (MDAR) Checklist for Authors. MDAR establishes a minimum set of requirements in transparent reporting in the life sciences (see Statement of Task: 10.31222/osf.io/9sm4x). Please follow the journal's guidelines in preparing your
Please note that a copy of this checklist will be published alongside your article.

Abridged guidelines for figures
1. Data
The data shown in figures should satisfy the following conditions:
- the data were obtained and processed according to the field's best practice and are presented to reflect the results of the experiments in an accurate and unbiased manner.
- ideally, figure panels should include only measurements that are directly comparable to each other and obtained with the same assay.
- plots include clearly labeled error bars for independent experiments and sample sizes. Unless justified, error bars should not be shown for technical
- if n<5, the individual data points from each experiment should be plotted. Any statistical test employed should be justified.
- Source Data should be included to report the data underlying figures according to the guidelines set out in the authorship guidelines on Data

2. Captions
Each figure caption should contain the following information, for each panel where they are relevant:
- a specification of the experimental system investigated (eg cell line, species name).
- the assay(s) and method(s) used to carry out the reported observations and measurements.
- an explicit mention of the biological and chemical entity(ies) that are being measured.
- an explicit mention of the biological and chemical entity(ies) that are altered/varied/perturbed in a controlled manner.
- the exact sample size (n) for each experimental group/condition, given as a number, not a range;
- a description of the sample collection allowing the reader to understand whether the samples represent technical or biological replicates (including how many animals, litters, cultures, etc.).
- a statement of how many times the experiment shown was independently replicated in the laboratory.
- definitions of statistical methods and measures:
  - common tests, such as t-test (please specify whether paired vs. unpaired), simple χ2 tests, Wilcoxon and Mann-Whitney tests, can be unambiguously identified by name only, but more complex techniques should be described in the methods section;
  - are tests one-sided or two-sided?
  - are there adjustments for multiple comparisons?
  - exact statistical test results, e.g., P values = x but not P values < x;
  - definition of 'center values' as median or average;
  - definition of error bars as s.d. or s.e.m.

Please complete ALL of the questions below.
Select "Not Applicable" only when the requested information is not relevant for your study.

Materials

Newly Created Materials	Information included in the manuscript?	In which section is the information available? (Reagents and Tools Table, Materials and Methods, Figures, Data Availability Section)
New materials and reagents need to be available; do any restrictions apply?	Not Applicable	

Antibodies	Information included in the manuscript?	In which section is the information available? (Reagents and Tools Table, Materials and Methods, Figures, Data Availability Section)
For **antibodies** provide the following information:<br>- Commercial antibodies: RRID (if possible) or supplier name, catalogue number and or/clone number<br>- Non-commercial: RRID or citation	Yes	Reagents and Tools Table

DNA and RNA sequences	Information included in the manuscript?	In which section is the information available? (Reagents and Tools Table, Materials and Methods, Figures, Data Availability Section)
**Short novel DNA or RNA including primers, probes**: provide the sequences.	Yes	Materials and Methods

Cell materials	Information included in the manuscript?	In which section is the information available? (Reagents and Tools Table, Materials and Methods, Figures, Data Availability Section)
**Cell lines:** Provide species information, strain. Provide accession number in repository **OR** supplier name, catalog number, clone number, and/**OR** RRID.	Not Applicable	
**Primary cultures:** Provide species, strain, sex of origin, genetic modification status.	Not Applicable	
Report if the cell lines were recently **authenticated** (e.g., by STR profiling) and tested for mycoplasma contamination.	Not Applicable	

Experimental animals	Information included in the manuscript?	In which section is the information available? (Reagents and Tools Table, Materials and Methods, Figures, Data Availability Section)
**Laboratory animals or Model organisms:** Provide species, strain, sex, age, genetic modification status. Provide accession number in repository **OR** supplier name, catalog number, clone number, **OR** RRID.	Yes	Reagents and Tools Table
**Animal observed in or captured from the field:** Provide species, sex, and age where possible.	Not Applicable	
Please detail **housing and husbandry conditions**.	Yes	Materials and Methods

Plants and microbes	Information included in the manuscript?	In which section is the information available? (Reagents and Tools Table, Materials and Methods, Figures, Data Availability Section)
**Plants:** provide species and strain, ecotype and cultivar where relevant, unique accession number if available, and source (including location for collected wild specimens).	Not Applicable	
**Microbes:** provide species and strain, unique accession number if available, and source.	Yes	Reagents and Tools Table

Human research participants	Information included in the manuscript?	In which section is the information available? (Reagents and Tools Table, Materials and Methods, Figures, Data Availability Section)
If collected and within the bounds of privacy constraints report on age, sex and gender or ethnicity for all study participants.	Not Applicable	

Core facilities	Information included in the manuscript?	In which section is the information available? (Reagents and Tools Table, Materials and Methods, Figures, Data Availability Section)
If your work benefited from core facilities, was their service mentioned in the acknowledgments section?	Not Applicable	

Design

Study protocol	Information included in the manuscript?	In which section is the information available? (Reagents and Tools Table, Materials and Methods, Figures, Data Availability Section)
If study protocol has been **pre-registered, provide DOI in the manuscript**. For clinical trials, provide the trial registration number **OR** cite DOI.	Not Applicable	
Report the **clinical trial registration number** (at ClinicalTrials.gov or equivalent), where applicable.	Not Applicable	

Laboratory protocol	Information included in the manuscript?	In which section is the information available? (Reagents and Tools Table, Materials and Methods, Figures, Data Availability Section)
Provide DOI OR other citation details if **external detailed step-by-step protocols** are available.	Yes	Materials and Methods

Experimental study design and statistics	Information included in the manuscript?	In which section is the information available? (Reagents and Tools Table, Materials and Methods, Figures, Data Availability Section)
Include a statement about **sample size** estimate even if no statistical methods were used.	Yes	Figures
Were any steps taken to minimize the effects of subjective bias when allocating animals/samples to treatment (e.g. **randomization procedure**)? If yes, have they been described?	Yes	Materials and Methods
Include a statement about **blinding** even if no blinding was done.	Yes	Materials and Methods
Describe **inclusion/exclusion criteria** if samples or animals were excluded from the analysis. Were the criteria pre-established?<br><br>If sample or data points were omitted from analysis, report if this was due to attrition or intentional exclusion and provide justification.	Not Applicable	
For every figure, are **statistical tests** justified as appropriate? Do the data meet the assumptions of the tests (e.g., normal distribution)? Describe any methods used to assess it. Is there an estimate of variation within each group of data? Is the variance similar between the groups that are being statistically compared?	Yes	Figures

Sample definition and in-laboratory replication	Information included in the manuscript?	In which section is the information available? (Reagents and Tools Table, Materials and Methods, Figures, Data Availability Section)
In the figure legends: state number of times the experiment was **replicated** in laboratory.	Yes	Figures
In the figure legends: define whether data describe **technical or biological replicates**.	Yes	Figures

Ethics

Ethics	Information included in the manuscript?	In which section is the information available? (Reagents and Tools Table, Materials and Methods, Figures, Data Availability Section)
Studies involving **human participants**: State details of **authority granting ethics approval** (IRB or equivalent committee(s), provide reference number for approval.	Not Applicable	
Studies involving **human participants**: Include a statement confirming that **informed consent** was obtained from all subjects and that the experiments conformed to the principles set out in the WMA Declaration of Helsinki and the Department of Health and Human Services Belmont Report.	Not Applicable	
Studies involving **human participants:** For publication of **patient photos**, include a statement confirming that consent to publish was obtained.	Not Applicable	
Studies involving experimental **animals**: State details of **authority granting ethics approval** (IRB or equivalent committee(s), provide reference number for approval. Include a statement of compliance with ethical regulations.	Yes	Materials and Methods
Studies involving **specimen and field samples:** State if relevant **permits** obtained, provide details of authority approving study; if none were required, explain why.	Not Applicable	

Dual Use Research of Concern (DURC)	Information included in the manuscript?	In which section is the information available? (Reagents and Tools Table, Materials and Methods, Figures, Data Availability Section)
Could your study fall under dual use research restrictions? Please check biosecurity documents and list of **select agents and toxins** (CDC): https://www.selectagents.gov/sat/list.htm	Not Applicable	
If you used a select agent, is the security level of the lab appropriate and reported in the manuscript?	Not Applicable	
If a study is subject to dual use research of concern regulations, is the name of the **authority granting approval and reference number** for the regulatory approval provided in the manuscript?	Not Applicable	

Reporting

The MDAR framework recommends adoption of discipline-specific guidelines, established and endorsed through community initiatives. Journals have their own policy about requiring specific guidelines and recommendations to complement MDAR.

Adherence to community standards	Information included in the manuscript?	In which section is the information available? (Reagents and Tools Table, Materials and Methods, Figures, Data Availability Section)
State if relevant guidelines or checklists (e.g., **ICMJE, MIBBI, ARRIVE, PRISMA**) have been followed or provided.	Not Applicable	
For **tumor marker prognostic studies,** we recommend that you follow the **REMARK** reporting guidelines (see link list at top right). See author guidelines, under 'Reporting Guidelines'. Please confirm you have followed these guidelines.	Not Applicable	
For **phase II and III randomized controlled trials**, please refer to the **CONSORT** flow diagram (see link list at top right) and submit the CONSORT checklist (see link list at top right) with your submission. See author guidelines, under 'Reporting Guidelines'. Please confirm you have submitted this list.	Not Applicable	

Data Availability

Data availability	Information included in the manuscript?	In which section is the information available? (Reagents and Tools Table, Materials and Methods, Figures, Data Availability Section)
Have **primary datasets** been deposited according to the journal's guidelines (see 'Data Deposition' section) and the respective accession numbers provided in the Data Availability Section?	Yes	Data Availability Section
Were **human clinical and genomic datasets** deposited in a public access-controlled repository in accordance to ethical obligations to the patients and to the applicable consent agreement?	Not Applicable	
Are **computational models** that are central and integral to a study available without restrictions in a machine-readable form? Were the relevant accession numbers or links provided?	Not Applicable	
If publicly available data were reused, provide the respective **data citations in the reference list**.	Not Applicable	
